# Multimodal Irregular Self-Selection in Chinese Postgraduate English as a Foreign Language Learners’ Conversation: When, How, and Why

**DOI:** 10.3389/fpsyg.2022.788438

**Published:** 2022-03-25

**Authors:** Mengmeng Ji, Huiping Zhang

**Affiliations:** School of Foreign Languages, Northeast Normal University, Changchun, China

**Keywords:** irregular self-selection, Chinese postgraduate EFL learners, descriptive statistics, multimodal conversation analysis, detailed process

## Abstract

Irregular self-selection is a demonstration of active involvement in interaction. English as a foreign language (EFL) learners’ talk-in-interaction is one of such cases. Yet, little research has explored when, how, and why learners implement this action. The aim of this article is to address these issues in Chinese postgraduate EFL learners’ conversations from the perspective of multimodal interaction. To this end, we provide descriptive statistics and use multimodal conversation analysis to investigate the detailed process of irregular self-selection. The results show the interactional sensitivity of learners, and all the successful irregular self-selection can be divided into the following three types: turn interruption (TI), turn competition TC), and turn holding abortion (THA). Learners implement this action by using multimodal resources, including lexis, syntax, pitch reset, intensity enhancement, gaze, and so forth. However, their body movements lack diversity, causing behaviors to be constrained and inactive. The main purpose of irregular self-selection is to provide knowledge that contributes to topical development. This study reveals that Chinese postgraduate EFL learners are interactionally competent members. They are able to achieve communicative goals but in a low diversity of body movements. The findings help to understand the detailed process of speakership claiming in EFL learners’ conversations.

## Introduction

Interaction is one of the core matrixes for human social life. A mechanism for coordinating interaction is a turn-taking system that regulates who is to speak and when (Sacks et al., 1974). Until now, a substantial amount of research has been conducted concerning how the system works either in ordinary or institutional conversation (e.g., Mondada, 2007, 2013; Stivers and Rossano, 2010; Weiß, 2018; Yang, 2019; Auer, 2021). In studies of turn-taking practices, conversation analysis (CA), which adopts a participant-relevant emic perspective, offers a valid tool for observing and analyzing the dynamic details of turn and sequence organization in talk-in-interaction. Recently, how multimodal resources work together in the construction and organization of turns has received much attention (Mondada, 2007, 2013; Streeck, 2009; Yang, 2011; Dressel, 2020). In line with this research, multimodal CA has largely remained a framework used by and shared with scholars in the social sciences.

However, mainstream multimodal CA research predominantly investigates first language interactions, and thus research on foreign language conversation is still marginalized in the literature (Pietikainen, 2018). In a few studies of non-native discourse, attention often turns to language classrooms since this is one of the main locations where foreign language learners have the opportunity and need to use the language they are learning (Waring, 2011). Yet, as noted by Kasper and Wagner (2011), language classrooms offer a limited range of interaction. Obviously, turn-taking in the classroom is under the guidance of the teacher, who has a high knowledge status and dominates the class interaction. Because of the low knowledge status and unequal relationship between teachers and students, self-selection is quite challenging and unusual in the teacher-student interaction context (Takahashi, 2018).

In contrast to it, peer interaction involves students who have equal identity status and similar knowledge status. In such a context, there is a tendency of English as a foreign language (EFL) learners to implement self-selection in an irregular turn-taking way, that is, irregular self-selection (Almoaily, 2020). However, by referring to the previous literature, relatively little is known about this action in EFL learners’ conversation from the perspective of multimodal interaction. To fill this gap, the present article aims to examine irregular self-selection in Chinese postgraduate EFL learners’ conversations by mainly using a multimodal conversation analytic approach. It tries to reveal the nature and essence of this action from when, how, and why to some extent. This article is expected to gain a deeper and more detailed understanding of Chinese postgraduate EFL learners’ irregular self-selection and add to the research on multimodal turn-taking practices of EFL learners. In all, we seek to address the following research questions:

(1) When does irregular self-selection occur (i.e., type)?
(2) How does the hearer mobilize multimodal resources (in verbal, vocal, and non-verbal aspects) to achieve irregular self-selection?
(3) Why do they implement irregular self-selection (i.e., purpose)?

## Literature Review

### Turn-Taking System and Irregular Self-Selection

A major feature of conversation is that people overwhelmingly talk in turns. The conversation analytic account of how turn-taking is managed has been noted by Sacks et al. (1974). They claim that the speech-exchange system consists of turn-constructional components and turn-taking rules. The characteristics of the turn-taking system can be summarized in three points as follows:

(1) Turn is made up of turn-constructional units (TCUs). These units can be lexical, phrasal, clausal, or sentential, used to complete a communicative act. The possible completion point (PCP) at the end of each TCU may become a place for speaker transition, called a transition relevance place (TRP), which is the appropriate position for turn-taking.
(2) An essential feature of TCU is its projectability, which allows participants to project where a turn will reach possible completion. A range of resources is used to project the possible completion of a TCU, including syntax, intonation, and pragmatics (Ford and Thompson, 1996). That is, a TCU has “syntactic, intonational, semantic, and/or pragmatic status as potentially complete” (Lazaraton, 2002, p. 32). To be specific, an utterance is grammatically complete if it could be interpreted as a complete clause in its discourse context. This utterance can be a word, a phrase, or a sentence. Intonation completion refers to a point at which a rising or falling intonation can be clearly heard as a final intonation. An utterance is pragmatically complete when it can be heard as a complete conversational action within its discourse context. When grammatical, intonational, and pragmatic completions of a TCU converge, a complex transition relevance place (CTRP) occurs (Ford and Thompson, 1996). CTRP is also where actual turn transitions are most likely to occur. Moreover, a substantial body of scholars notes that non-verbal conducts also figure in projecting TCU completion, such as eye gaze, open hand palm up (OHPU), pointing gesture, and so on (Kendon, 1967; Goodwin, 1980; Mondada, 2007; Streeck, 2009; Li, 2014).
(3) The organization of turn-taking obeys three rules. Rule 1: Current speaker selects the next; Rule 2: Other participants self-select for the next speaker; Rule 3: If no hearer takes the turn, the current speaker should continue the turn until the next speaker takes it. These “gross observations” are critical in understanding how turn-taking is managed and “when a turn is complete from the participants’ perspective” (Greer and Potter, 2008, p. 299). By adhering to turn-taking rules, the ideal condition of turn-taking is featured as only one party speaking at a time, avoidance of overlapping talk, and the minimization of gaps and silences between turns.

Self-selection is one of the efficient ways to participate in a conversation and gain the speakership actively. The basic principle for the next speaker’s self-selection is to “start as early as possible at the earliest transition relevance place” (Sacks et al., 1974, p. 719). However, because of the dynamic and unpredictable features of interaction, participants do not always adhere to the turn-taking system to participate in a conversation. Thus, the ideal condition of turn-taking cannot be guaranteed, “turn-taking irregularities” (Almoaily, 2020, p. 188) are of frequent occurrence. Violating the turn-taking system, irregular self-selection is mainly characterized as a form of interruption or overlap, which occurs in the following two circumstances: C1. The hearer may self-select at non-TRPs to achieve a certain communicative goal, especially when the speaker is still speaking or has no intention to give up the speaking turn (i.e., not project the turn completion). This kind of behavior results in an interruption in a conversation. C2. The hearer may self-select at a possible TRP (rule 2), which simultaneously occurs with the current speaker’s application of rule 3 (i.e., the current speaker’s continuation) (Konakahara, 2020). This kind of behavior results in overlap in a conversation. All of them reflect the dynamic and unpredictable nature of interactions and are vital to the understanding and study of irregular self-selection.

### Multimodality of Self-Selection

In earlier CA research, embodied actions are considered to have a subsidiary role in interaction as “a hand-maiden to speech” (Streeck, 2009, p. 26), but more recent work has started to consider bodily actions to be as important as talk (Streeck et al., 2011). A substantial body of scholars have confirmed that a large range of interactional resources is relevant to the organization of turn-taking, encompassing linguistic resources such as lexis, grammar, prosody (Ford and Thompson, 1996), as well as embodied multimodal resources such as eye gaze (e.g., Kendon, 1967; Goodwin, 1980; Goodwin and Goodwin, 1986; Rossano, 2012), gesture (Mondada, 2007; Streeck, 2009; Yang, 2010; Li, 2014), head movement (Markaki and Mondada, 2012; Li, 2019), and body posture (Mondada, 2013; Li, 2014).

With regard to self-selection, “a possible next speaker may start gearing up for his or her turn before the current speaker’s turn completion” (Lee, 2017, p. 672), and the participants use multimodal resources to implement this action in various contexts. For example, in a multi-party conversation, the linguistic resources “*I’m sorry (to interrupt)*” are used as self-selection devices to obtain the speakership (Park and Duey, 2020). The pointing gesture of the hearer also severs the action-projecting function to “self-selection for would be next speakers” (Mondada, 2007, p. 207). In a teacher-fronted classroom (Sahlström, 2002; Lauzon and Berger, 2015; Takahashi, 2018), Sahlström (2002) reported that students used hand raising to self-select as the next speaker. In ordinary conversation (Streeck and Hartge, 1992; Iwasaki, 2009), Streeck and Hartge (1992) observed that facial configurations display the speakers’ intent. For example, facial expression (a) was used as a self-selection device among Ilokano speakers in their interactions.

Although most of the studies focus on self-selection actions in first language conversations, the remaining non-native speakers’ interactions are somewhat marginalized. It must be emphasized that EFL learners, “despite their limited proficiency in the target language, are interactionally competent members who manage to participate in discussions” (Lee, 2017, p. 673). For instance, Carroll (2004) observed that Japanese novice speakers of English used recycled turn beginnings (words) in ways similar to those of native speakers of English as a self-selection device. Moreover, non-verbal resources such as gestures, gaze orientation, and posture are also used by learners to show participating interests in conversation (Olsher, 2004; Konakahara, 2015, 2020; Taleghani-Nikazm, 2015; Lee, 2017; Majlesi and Markee, 2018). For instance, Lee (2017) found that learners used to gaze and gesture to prepare for self-selection.

In line with the multimodality of self-selection, irregular self-selection has the same nature. Moreover, this action has been reported as a demonstration of active involvement in interactions, with EFL learners’ talk-in-interaction being one of such cases (Cogo and Dewey, 2012; Konakahara, 2015). Explorations of irregular self-selection could enrich multimodal CA-based turn-taking studies. However, relevant research is still scarce in this field, especially in EFL learners’ conversations. Thus, more investigations are needed.

### Irregular Self-Selection: When, How, and Why

To begin with, scholars have conducted a few studies on when, how, and why learners implement self-selection. They are referential to the study of irregular self-selection. Orletti (1981) reported that self-selection occurs during a pause or after another speaker has completed the previous turn. Referring to how, Richard and Nunan (1990) demonstrated that self-selection could be achieved linguistically, non-verbally, pragmatically, and tactically. As for why, Waring (2011) reported three types of self-selections: to initiate a sequence, to volunteer response, and to proceed with the agenda. Furthermore, Garton (2012) found that confirmation checks, clarification requests, and information requests were the three most common uses of self-selection. The existing findings are beneficial to understanding the nature of participants’ self-selection.

Recently, scholars have been interested in how learners engage embodied resources in self-selection. For instance, Lee (2017) investigated the multimodal resources used by EFL learners to gain primary speakership within their peer group discussions. This study showed that the hearer actively moved into the primary speaker position by utilizing an ensemble of talk, gaze, gesture, and bodily orientation. For instance, learners used to gaze and gesture to claim for the speakership and to prepare for self-selection and used touch to interrupt the ongoing talk to join the conversation. It must be emphasized that both regular and irregular self-selection are a crucial part of the learning process because they both allow learners to claim speakership for the exchange of views, analyses, and opinions. However, the analysis of irregular self-selection is scarce, and only a few studies have explored it in learners’ conversations (e.g., Guillot, 2009, 2012; Konakahara, 2015, 2020; Lee, 2017). For example, Konakahara (2015) examined the interactional environment in which overlapping questions occur (i.e., when) and the interactional functions they serve (i.e., why). He found that this kind of irregular self-selection results from the simultaneous application of a next speaker’s self-selects, and the current speaker continues turn-taking. Moreover, without clinging to the overlap, participants cooperatively moved the talk forward. Konakahara (2020) further reported two kinds of irregular self-selections (i.e., floor-taking overlap and floor-attempting overlap) from when and how, but this study did not consider why. Lee (2017) investigated how learners used touch to interrupt the ongoing talk to join the multi-party interaction. However, when and why has not been concluded in the study.

Previous literature reveals that irregular self-selection research is still insufficient. Thus, the aim of the present study is to enrich the research of this action. We focus on relatively naturally occurring peer conversations among Chinese postgraduate EFL learners, to illustrate their irregular self-selection. This study provides overall descriptive statistics of the number, type, and purpose of this action, and then uses single-case analysis and a multimodal conversation analytic approach. It exemplifies the process of irregular self-selection from when, how, and why in detail by analyzing their turn construction and sequence organization. The study contributes to the growing body of knowledge of the multimodal nature of EFL learners’ interaction. It also helps to understand what they actually do to achieve successful outcomes in different interactional contexts.

## Materials and Methods

### Participants

This study involved 40 Chinese postgraduate EFL learners (4 men and 36 women), who were in the first year of their master’s program in September 2020. Their average age was 23 years (*SD* = 1.48; range 21–27). They generally shared the same first language background (Chinese) and had studied English for about 13.6 years on average (*SD* = 2.23; range 10–18). Their overall English language proficiency can be characterized as high, because they had passed the Test for English Major-8 (TEM-8) with 70.5 points out of 100 on average (*SD* = 4.92; range 65–80), and those who can reach 60 points are identified as advanced EFL learners in China. Before recording, informed consent was obtained from the participants at the time of the recruitment, and they volunteered to participate with great zeal.

### Data Collection

Before collecting data, participants were not informed of the general study purpose. We only informed them of the video recording and required them to carry on an ordinary, casual, and natural conversation as much as possible. The data are at best characterized as “non-pedagogic casual talk” (Carroll, 2004, p. 203), because it was collected after class and was chatted among friends who are familiar with each other. In dyadic dialogue, the participants finally formed 20 peer-to-peer conversation groups by adopting a free combination at their own will. Each group chose one of the 10 topics to discuss, such as “friends,” “travel,” and “traditional Chinese festival,” which had been delivered to them in advance for preparation. The topics were slightly general to give the participants enough “chat space” to show their interactional ability.

The data was collected in a quiet room which is commonly used by these participants. They were requested to sit comfortably close to each other, with the video camera placed about 1 m away on a tripod in front of them, and a voice recorder placed behind them. The conversational interactions among participants were recorded by the current first researcher utilizing “non-participant observations” (Davies, 2007, p. 174). That is, the researcher does not participate in the discussion, but instead sits in a corner hidden from the participants’ view, to observe their behaviors without interference. In the study of multimodal interaction, facial expressions, gestures, head movements, and body movements of the participants are all important information. Therefore, their upper bodies were mainly captured by the closed-set-up video camera. Furthermore, the sound was also recorded by the high-quality voice recorder used to conduct prosody analysis. For each recording, the researcher started the video recording, checked the audio, and then sat. The participants could freely begin and end their conversations without a time limit. In all, the duration time of each conversation varied from 8 to 23 min, and the total communication time was 295 min, with 31,759 words.

### Single-Case Analysis and Multimodal Conversation Analysis

A single-case analysis means “the techniques of seeing significant interactional detail in the ongoing production of singular sequences of talk-in-interaction” (Hutchby and Wooffitt, 2008, p. 113). Its goal is to explain a single complex phenomenon of interest. This approach can be enhanced further by combining with multimodal CA (Mondada, 2018). As Mondada (2018, p. 86) puts it, multimodal CA pays “careful and precise attention […] to temporally and sequentially organized details of actions that account for how co-participants orient to each other’s conduct and assemble it in meaningful ways, moment by moment.” Multimodal CA allows analysts to detailly identify a range of interactional resources that interactants utilize and organize to achieve communicative goals in the extended sequences of talk, from participant-relevant emic and multimodal perspectives. Overall, the combined approach can help us to enrich our understanding of the learners’ irregular self-selection deeply and detailly to some extent, especially the interplay of verbal and non-verbal resources in specific interactional contexts.

### Data Analysis

To address the research questions, four analytical procedures were followed:

(1) The simplified Jeffersonian convention (Jefferson, 2004) was used to transcribe verbal behavior in the data by the first author with help of Transcriber software (Boudahmane et al., 2022) (see Appendix). To improve the accuracy and reliability of the transcribed data, the two researchers worked together to check it over. Then, we conducted a line-by-line analysis, in a larger sequence closely examining what and when the participants said. We narrowed the focus down to sequences in which the participants implemented irregular self-selection to speak next and gathered a collection of all such turns (i.e., number).
(2) With reference to the two aforementioned circumstances of irregular self-selection, we carefully analyzed and coded each case within the collection. This round of analysis yielded findings of the types. We counted the frequency of each type and recorded it in an Excel sheet. Adhering to the purposes of self-selection classified by Waring (2011) and Garton (2012), we conducted another round of analysis and coding of the successful irregular self-selection, guided by the question: “Why that now?” (Schegloff and Sacks, 1973, p. 299). Through classification, comparison, and modification, this round of analysis yielded findings of the purposes. We counted the frequency of each purpose and recorded it in the Excel sheet. During the process of analysis, the inter-rater agreement of the two researchers was over 80%.
(3) To further explore the issues of when, how, and why, the single-case analysis combined with multimodal CA was used to carefully examine three excerpts of irregular self-selection representing the types, respectively. A slightly modified version of Mondada’s (2016) annotation was used to describe the embodied actions within interactions (see Appendix). Details about participants’ body movements were noted on a separate line above the verbal line in the transcript. Screenshots were also used to show the participants’ body movements capturing the moment of when and how, and their specific occurrences were noted with a “#” in the transcript.
(4) To analyze the prosodic features of irregular self-selection, particularly focused on pitch and intensity, we used *Praat* software, a combination of auditory and acoustic analysis (Boersma and Weenink, 2021). The form of spectrogram, waveforms, pitch traces, and intensity traces were all analyzed by this software. They are the compelling evidence that self-selector claims for turn space (Schegloff, 2000).

## Results

In this part, we first presented the overall descriptive statistics of number, type, and purpose of irregular self-selection. Then, three representative excerpts were analyzed in detail using multimodal CA to further address the issues of when, how, and why.

### Descriptive Statistics

#### Number

Through repeated line-by-line observation and analysis of the transcribed data, this study yielded 152 cases of irregular self-selection. As Table 1 showed, in a total of 20 groups, seventeen groups contained irregular self-selection, ranging from 1 to 38 cases in each conversation. According to the number of cases, the participation model of seventeen groups could be characterized as conventional, active, and highly active. Among them, five groups contained irregular self-selection in less than 5 cases, indicating that turn-taking devices in these groups were more conventional because they tended to obey turn-taking rules and used less irregular self-selection devices to take turns. The groups containing cases in 5–9 were the most, including eight groups. These groups were active because more interruptions or overlaps occurred in their conversations, indicating that the participants more actively joined in the discussion. Finally, four groups had cases over 10, and the most were 38 cases, indicating the highly active participation of the learners in the interaction. This means that hearers in these groups were more eager to obtain the speakership. The uneven distribution of irregular self-selection among different groups reflected the group or individual discrepancy of participation in interaction. In summary, seeing from the overall data, the Chinese postgraduate EFL learners were relatively active in participating in peer conversations.

**TABLE 1:** Number of irregular self-selection in each group.

Group (G)	G1	G2	G3	G4	G5	G6	G7	G8	G9
Number	9	18	38	1	6	2	12	1	7

**Group (G)**	**G10**	**G11**	**G12**	**G13**	**G14**	**G15**	**G16**	**G17**	

Number	5	9	8	3	5	6	4	18	

#### Type

According to the two aforementioned circumstances of irregular self-selection in the literature review, we divided 152 cases into four types (see Table 2). The frequency of each type was reported in Table 3.

**TABLE 2:** Types of irregular self-selection.

Type	Meaning	Example
Turn interruption (TI)	The current speaker’s turn is at a non-TRP, signified by a uncomplete TCU, the hearer interrupts to obtain the speakership. (C1).	A: “Where are you come from, that’s” B: “Or what’s your name”
Turn competition (TC)	The current speaker’s turn reaches the TRP. Then, the two participants speak simultaneously to compete for speakership. It is the hearer who wins the competition for the turn space here. This kind of situation is a result of application of rule 2 and rule 3 as described in C2.	A: “So have you some uh did you have some maybe some hum example teacher [in your]” B: “[uh]You know yes here is one.”
Turn holding abortion (THA)	The current speaker’s turn reaches TRP. However, the speaker has no intention of giving up the speakership by using non-lexical words, such as hum/uh/mm. At this time, the hearer chooses to speak to abort this turn holding process to obtain speakership. (C1).	A: “I can hold parties many times hum” B: “There must be a garden in your house”
Self-selection failed (TF)	The hearer fails to gain the speakership when he/she self-selects. (C1 or C2).	A: “I hope all of us can” B: “Can” A: “Find a Mr. Right.”

*All the examples in Tables 2, 4 are real cases that occurred in the participants’ conversations.*

**TABLE 3:** Frequency of types.

Type	TI	TC	THA	TF	Total
Number	96	37	10	9	152
Percentage	63.4%	24.2%	6.5%	5.9%	100%

As shown in Table 2, successful irregular self-selections mainly occurred in three interactional contexts: (1) when the speaker’s turn was at a non-TRP, the hearer interrupted to obtain the speakership; (2) the two participants competed for the speakership; and (3) when the speaker was holding the current turn, the hearer chose to speak to abort this turn holding process, and then gain the speakership. The detailed multimodal process of those irregular self-selections will be analyzed in the subsequent section.

Table 3 revealed various types of irregular self-selection. The most frequent type was TI, consisting of 96 cases (63.4%) of all the irregular self-selection. TC came next, with 37 cases (24.2%). THA came third, with only 10 cases (6.5%). Because of the small percentage of the last type, TF, where hearer failed to gain the speakership, we excluded them from the analysis.

#### Purpose

With reference to the classifications of Waring (2011) and Garton (2012), and combining them with the actual cases that occurred in the present data, we divided the purposes of irregular self-selection into six types, as shown in Table 4. The frequency of each type was reported in Table 5.

**TABLE 4:** Purposes of irregular self-selection.

Purpose	Meaning	Example
Aiding	To help the current speaker when he/she faces some expression difficulties (e.g., disfluency or pause)	A: “Hum, I think the challenges means more. (2.3)” B: “Means more chances”
Cooperative completion	To cooperatively complete the following turn content with the current speaker based on contextual information	A: “The character acted by the by [Zhou Dong Yu]” B: “[Zhou Dong Yu]”
Displaying knowledge	To display own thoughts and views or providing comments and new information contributing to the topical development	A: “You can also listen some informal materials such as the Allen Show or Friends [this]” B: “[Yeah] they are popular.”
Agreement	To express agreement and support of the current speaker’s speech	A: “He usually she usually do some small punishment to us uh then” B: “Yes I agree with you”
Clarification	To request the current speaker to clarify some vague information in the previous turn in order to reach a mutual understanding, usually by using some lexical bundles like *you mean X*	A: “So he (0.5) [pro-]” B: “[You mean] leave her family a big fortune?”
Information request	To elicit further information that relates to the ongoing topic based on the previous utterance/sequence, usually by using interrogative sentence	A: “We waited a very long time, very very very long, so [in that]” B: “[Is in] midnight?”

**TABLE 5:** Frequency of purposes.

Purpose	Aiding	Cooperative completion	Displaying knowledge	Agreement	Clarification	Information request	Total
Number	18	11	76	15	7	16	143
Percentage	12.6%	7.70%	53.1%	10.5%	4.90%	11.2%	100%

In accordance with Table 4, we calculated the frequency of each type in participants’ conversations, and the result was reported in Table 5.

As can be seen in Table 5, the most important purpose of irregular self-selection was to display knowledge, which had 76 cases (53.1%). It indicated that irregular self-selection was used by the hearer to show understanding of and interest in what had been said, and then displayed their own thoughts and views or provided comments and new information. All of them can contribute to topical development. The purposes of aiding and cooperative completion had 18 cases (12.6%) and 11 cases (7.7%), respectively. They both indicated high participation of the hearer in the conversation to help or cooperate with the speaker to complete their current turn. The information request purpose had 16 cases (11.2%). By asking a question, the hearer attempted to show interest in what has been said and tried to elicit further information relating to the ongoing topic from the speaker. The agreement purpose had 15 cases (10.5%), which showed the hearer’s attentive listening and understanding of what had been said. The last one, clarification purpose, only had seven cases (4.9%), indicating the negotiation of information between participants to achieve mutual understanding.

Having shown the overall number, type, and purpose of irregular self-selection, the next section will analyze three representative excerpts of successful irregular self-selection in detail to further illustrate when, how, and why the self-selectors implement them.

### Case Analysis of Irregular Self-Selection Using Multimodal Conversation Analysis Method

The focal episodes of the analysis centered on the following three types: turn interruption (TI), turn competition (TC), and turn holding abortion (THA). In what follows, by focusing on three representative episodes in as much detailed as possible, we showcased when, how, and why self-selectors accomplished self-selection in the peer-to-peer conversation by using the multimodal CA method.

#### Turn Interruption

This subsection shows the analysis of the first type of irregular self-selection, that is, TI, one of the practices frequently used by self-selectors. TI means that the hearer implements self-selection when the speaker’s turn is at non-TRP. More specifically, when the speaker is still in the state of event narrating or storytelling, the obvious sign is that the TCU is incomplete. However, at this time, the hearer self-selects to speak, resulting in a TCU being interrupted before it has reached a point of possible completion, as shown in excerpt 1.

Excerpt 1. Rubbish Sorting
01 W U:m I have heard that like Beijing and Shanghai
02 um they have put forward the (.) project
Hand_L_ -.-.-.-.-.
Torso_L_ F—-
03 like #hum

Gaze mutual gaze
Hand_L_^****^
Torso_L_ H—-
04 L #Ah
05 W [rubbish classification]
06 L [rubbish classification]
07 W yeah
08 L yeah

a. When

**FIGURE 1:**
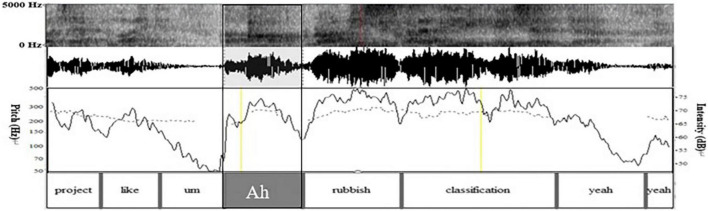
Spectrogram, waveform, pitch trace (dotted line), and intensity trace (solid line) of lines 02–08 in excerpt 1.

**FIGURE 2:**
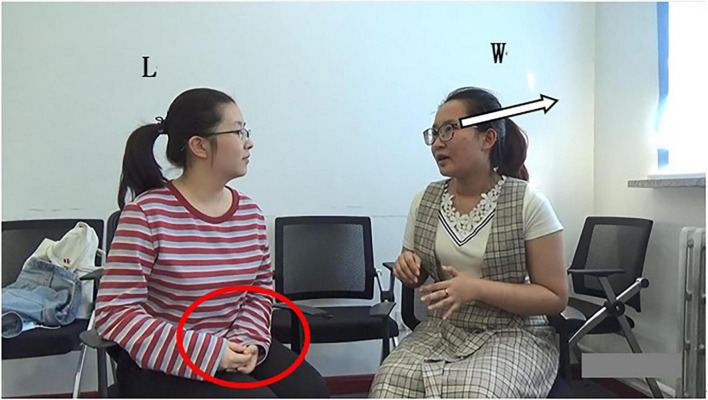
Bodily movement of W in line 03.

In lines 01–02, W describes the garbage management scheme proposed by the environmental institutions in Beijing and Shanghai. She makes a concrete elaboration of the program in line 03. Grammatically, W uses preposition “*like*,” but due to the lack of object, it does not constitute the complete preposition phrase; Prosodically (Figure 1), as shown in pitch trace, the word “*like*” is in flat intonation, which generally indicates the maintaining of the turn and shows that this turn has not ended (Ford and Thompson, 1996); Pragmatically, W does not elaborate the specific scheme, and thus the statement is incomplete. Non-verbally, W’s gesture is still away from the “home position” (Sacks and Schegloff, 2002), and she does not gaze at the recipient L at the end of her turn (Figure 2). The above multimodal resources show that W’s current turn is not complete in terms of syntax, intonation, pragmatic behavior, and body movement. So, the turn does not reach TRP at the current moment, implying that turn-taking does not occur here.

However, hearer L gets the thought W wants to express based on the contextual information, and then she makes her own choice to interrupt L’s speech and self-select to gain the speakership (lines 04–06).

b. How

**FIGURE 3:**
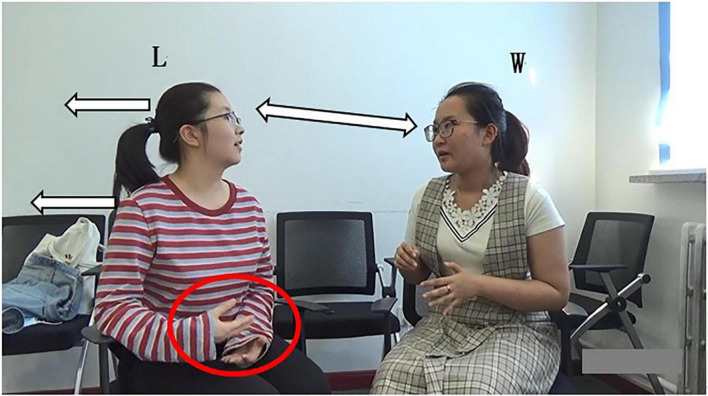
Bodily movement of L in self-selection in line 04.

(1) Grammatically, L uses an exclamatory word plus noun phrase, which is “*Ah*” and “*rubbish classification*” as self-selection words. First, she produces the non-lexical token “*Ah.*” On the one hand, “*Ah*” is used to show a change of state indicating she has known what W wants to say. On the other hand, it is used to attract W’s attention and show her interest and attentive listening to the current conversation. Moreover, *Ah* can be seen as a kind of self-selection signal showing her follow-up participation. Then, the noun phrase “*rubbish classification*” overlaps with W’s words, that is, they speak it simultaneously; (2) Prosodically (Figure 1), as analyzed by Praat, L makes a pitch reset and an intensity enhancement. First, through pitch reset, the pitch of self-selection words becomes higher. As can be seen in pitch trace that the peak pitch at “*Ah*” is 270 Hz, which is higher than the pitch at the previous word “*hum*” (207 Hz). Second, through the intensity enhancement, the speech loudness increases; namely, the sound becomes louder. Intensity trace suggests that the peak intensity at “*Ah*” is 75 dB, which is louder than that at *hum* (50 dB). Finally, the color of the spectrogram at “*Ah*” becomes darker and acoustic amplitude becomes larger. They indicate that the energy value at this place becomes larger, which supports the above findings. (3) Non-verbally, L uses gaze, gesture, body posture, and head movement when she prepares for self-selection. Her constructions of complex multimodal “gestalt” (Li, 2014, p. 7) are assembled simultaneously. Specifically, L and W at mutual gaze status, her gesture gradually deviates from home position (Figure 3), and upper body posture and head position change from leaning forward to relaxed position (Figures 2, 3). The changes in body posture and head position, simultaneously with the self-selection signal “*Ah*,” indicate that she has recognized the thought W wants to express. Overall, L uses lexical phrases, pitch reset, intensity enhancement, and non-verbal resources including gaze, gesture, body posture, and head movement to project and achieve self-selection.

c. Why

As can be seen in conversation sequences (lines 01–06), L’s self-selection purpose is to cooperate with W to complete the specific rubbish management scheme mentioned in the previous turn, namely rubbish classification (lines 05–06). After this, L’s self-selection words get W’s approval by using the acknowledgment token “*yeah*” (line 07). In all, the self-selection does not cause trouble to the current conversation, rather than show the interests and active participation of L. It also demonstrates her interactional sensitivity, turn-monitoring awareness, and participation ability in the conversation. Moreover, the overlapped noun phrase “*rubbish classification*” is recognitional overlap, which is a type of overlap that occurs when a potential next speaker recognizes the “thrust or upshot” of the prior talk (Konakahara, 2015, p. 39). It is usually considered legitimate or non-intrusive within the turn-taking system.

#### Turn Competition

The second type of self-selection is TC, causing overlap. It occurs as a result of the hearer’s application of rule 2 at a possible TRP (i.e., self-selection), simultaneously occurring with the speaker’s application of rule 3 (i.e., the current speaker’s continuation). Moreover, sometimes it occurs with the speaker’s multimodal resources “divergence” from each other (Li, 2014, p. 205). That syntax, prosody, pragmatic behavior, and gaze resources project the end of the turn, indicating the occurrence of TRP and turn-taking. However, divergence from turn-ending projection, gesture projects the turn holding, implying that the current speaker is not ready to transfer the turn, as shown in excerpt 2.

Excerpt 2. School Bullying
01 J I also think hum our country should carry out some relevant laws
02 to about this kind hum event
03 J so that our teenagers and even (.)
Gaze_J_ _away | at_
Hand_J_^***********************************^
04 college students can be protected hum #well(0.3)

Gaze mutual gaze
Hand_J_^*********^| -.-.-.
05 Y [yes I think #so]
Hand_J_^*********^| -.-.-.-.–.-.-.-.-.-.–.-.-.-.-.-.-.
06 J [that’s my #per°sonal°] understanding
07 Y and I think the students should stand up
08 to stop this phenomenon

a. When

**FIGURE 4:**
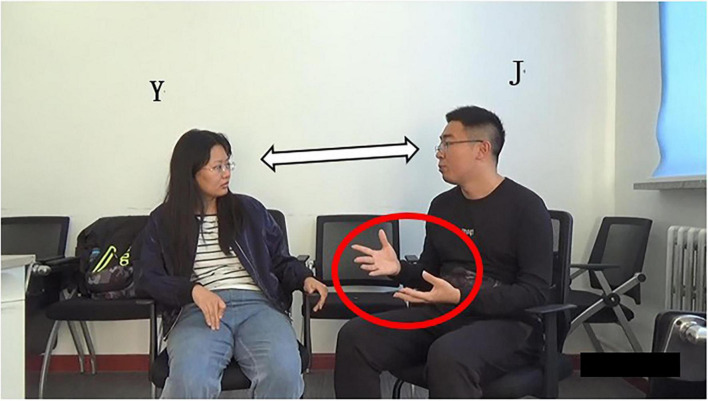
Bodily movements of Y and J in lines 04–05.

In lines 01–04, J states that the Chinese government must promulgate laws and policies to protect teenagers from school bullying, including college students. Grammatically, it is an adverbial clause directed by “*so that*,” with complete syntactic structure; Prosodically, the sentence can be judged to be in falling intonation by combining with listening and discrimination. In addition, a pause of 0.3 s following “*well*” suggests that turn-taking may occur (Ford and Thompson, 1996); Pragmatically, J’s declarative statement is complete and expresses his point of view; Non-verbally, J looks at Y at the end of the turn (the word “well”), forming a mutual gaze with Yang at the same time (Figure 4), which projects possible end of the turn (Kendon, 1967). In all, the above four multimodal resources indicate that J’s turn may end and arrive at TRP at this moment and turn-taking may occur.

**FIGURE 5:**
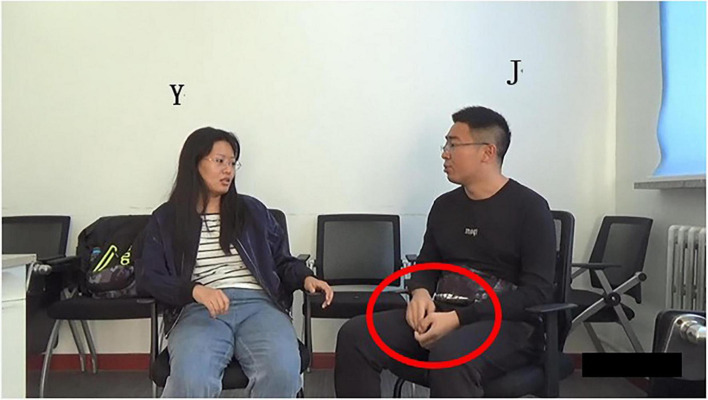
Gesture of J after vertical bar in lines 05–06.

However, J’s gesture does not return to the home position but still keeps the “open hand palm up (OHPU)” (Li, 2014, p. 219; Figure 4), implying turn holding. It can be seen that the gesture resource of J is in conflict with the aforementioned four kinds of multimodal resources. All the other resources indicate turn completion and occurrence of turn-taking, while gesture indicates turn holding; that is, J still wants to talk and has no intention of giving up the speaking turn. A noteworthy observation is that J’s OHPU gesture in the current turn lasts about 15 s in the video, almost throughout his whole turn. It means from the perspective of J’s gesture using habit, he prefers it. Previous studies have shown that the OHPU gesture usually appears at the possible end of a turn, indicating the yielding of that turn (Streeck, 2009). However, in this case, the OHPU gesture is J’s habitual practice; hence, it does not indicate the end of the turn but implies the holding of this turn. The intention can also be seen in J’s following actions. In lines 05–06, J’s turn overlaps with Y’s. They compete for the speakership, but Y wins the competition for the turning space here. In addition, J returns his gesture to the home position at the word “*so*” and *“personal*” in lines 05 and 06 (Figure 5) and completes the turn of himself after overlapping resolution (line 06). Thus, it shows that J wants to continue his turn, and only the return of gesture indicates the possible end of the turn (Li, 2014). This excerpt reflects whether noticing the individual discrepancy is an essential factor in deciding self-selection time. It is more unpredictable and needs the hearer to monitor the turn momentarily. Just as in this case, Y ignores the diverging of gesture and thus implements self-selection to show her understanding of and interests in what has been said by J.

b. How

**FIGURE 6:**
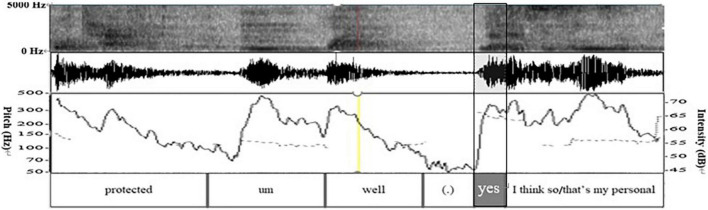
Spectrogram, waveform, pitch trace (dotted line), and intensity trace (solid line) in lines 03–05 in excerpt 2.

(1) Grammatically, Y uses the acknowledgment token “*yes*” to express her approval of J’s statement, followed by the sentence “*I think so.*” (2) Prosodically (Figure 6), the software Praat shows that Y makes a pitch reset but hardly makes an intensity enhancement. First, pitch trace shows that the pitch at the end of J’s turn is about 124 Hz, while the pitch at *yes* is 176 Hz (300 min 124 Hz), which is 52 Hz higher than that at the end of J’s turn. Second, as can be seen in the intensity trace, the intensity at *yes* is about 27 dB (72 min 45 dB), which is lower than 45 dB at the end of J’s turn. Last, the color of the spectrogram at *yes* becomes darker, and the amplitude of the sound wave in the acoustic map becomes larger. They indicate that the energy value at *yes* becomes larger, which supports the occurrence of the overlapped speech of Y and J here. (3) Non-verbally, Y implements self-selection while at a mutual gaze state with J (Figure 4). In all, Y uses lexis, syntax, pitch reset, and gaze to achieve self-selection.

c. Why

The conversation sequence (lines 03–04) shows that the purpose of Y’s self-selection is to express her agreement with J’s statement by using the word “*yes*” and the sentence “*I think so.*” It shows her active participation in the current topic.

#### Turn Holding Abortion

The usage frequency of the third type of irregular self-selection is less than the aforementioned two ones, but it is still a way for the hearer to gain the speakership. This type is THA, wherein when a speaker uses some multimodal resources to indicate the continuity of speakership, the hearer implements self-selection to obtain the speakership. The continuation of talk is represented by the use of the non-lexical word “*hum*,” gaze shift, and the holding of “thinking face” (Goodwin and Goodwin, 1986, p. 57). As illustrated in excerpt 3.

Excerpt 3. House
01 M I will buy hum a very very big house
02 and I can hold (.) many parties.
Gaze_M_ away
03 #hum

Gaze mutual gaze
04 N #there must be a garden in your (.) home(.)
05 M yes

a. When

**FIGURE 7:**
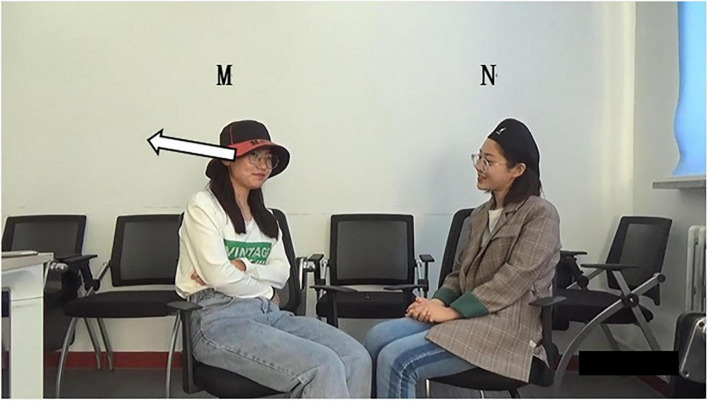
Gaze of M in line 03.

In lines 01–02, M states that she wants to buy a big house in the future so that many parties can be held in it. Grammatically, this sentence is a compound sentence combined by a connective *and*, with complete syntactic structure. Prosodically, the point number shows that the second part of the sentence is in falling intonation, indicating the possible end of the sentence (Ford and Thompson, 1996). Pragmatically, M’s declarative statement is complete, and she finishes her opinion of the house. At this time, her turn is complete in grammar, prosodic, and pragmatic behavior. The above multimodal resources indicate a high possibility of turn completion and turn-taking. However, instead of ending the turn here, M uses several turn holding strategies to indicate the continuity of the current turn, including non-lexical word “*hum*,” gaze shift, and holding of a thinking face (Figure 7). To facilitate the back-and-forth flow of a natural conversation, N participates in the conversation actively to self-select and to gain the speakership, which shows her turn controlling awareness (line 04). Moreover, since the decision-making of self-selection lies with the hearer, the timing of it is related to participation state, knowledge, or emotional status (Heritage, 2013).

b. How

**FIGURE 8:**
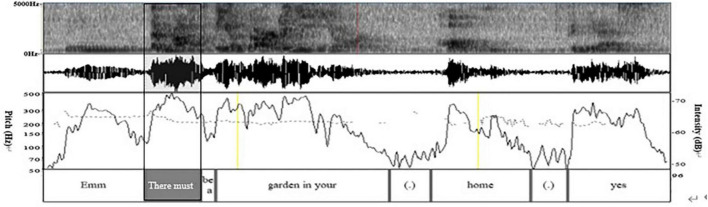
Spectrogram, waveform, pitch trace (dotted line), and intensity trace (solid line) in lines 03–05 in excerpt 3.

**FIGURE 9:**
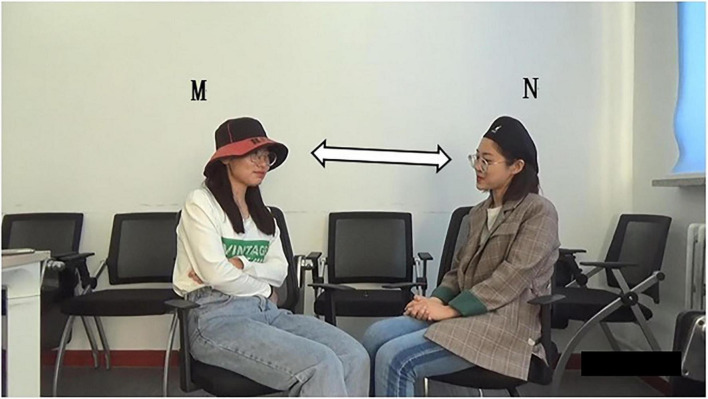
Bodily movement of N in self-selection in line 04.

(1) Grammatically, N uses the existential sentence guided by *“there be.*” (2) Prosodically, in Figure 8, the pitch trace shows that N does not reset the pitch but maintains the same pitch range just as M has (about 250 Hz), so the pitch changes slightly. However, N enhances the intensity of the words “*there must*” to draw the attention of speaker M. In Figure 8, the peak value of the intensity at “*there must*” is 71 dB, higher than that in *hum* at the end of the M’s turn (60 dB). In addition, the color of the spectrum at “*there must*” becomes deeper and the amplitude of the sound wave in the acoustic map becomes larger. They indicate the increment of the energy value, which are proofs of the above findings; (3) Non-verbally, N and M form mutual gaze when she implements self-selection (Figure 9). In all, N uses syntax, intensity reset, and mutual gaze to achieve self-selection.

c. Why

Conversation sequence (lines 01–04) reveals that the self-selection sentence “*There must be a garden in your house*” is used for displaying knowledge and functions as a supplement of new information to M’s speaking content. It can promote topical development (line 04). M then acknowledges this with acknowledgment token *yes* (line 05).

## Discussion

As an important turn-taking method and conversation monitoring strategy, the implementation of irregular self-selection reflects certain interactional features of Chinese postgraduate EFL learners. We explore it by providing overall descriptive statistics of this action and then conducting a detailed analysis (i.e., multimodal CA) of three representative examples of irregular self-selection from when, how, and why. The results are of great significance to enrich the existing research on turn-taking practices of EFL learners from the perspective of multimodal interaction.

First of all, regarding when to self-selection, as shown in the number of irregular self-selections of each group, in a total of twenty groups, seventeen groups contain irregular self-selections, varying from 1 to 38 cases in each conversation. The participation mode of seventeen groups can be characterized as conventional (five groups), active (eight groups), and highly active (four groups). To summarize, 85% of groups contain irregular self-selections, and 60% of groups are active in implementing such actions to participate in the conversation. The results are in line with the evidence that EFL learners are interactionally competent members to participate in a conversation (see Carroll, 2004; Firth and Wagner, 2007; Lee, 2017; Konakahara, 2020). For example, Lee (2017) found that learners actively interrupted the ongoing talk to move into the primary speaker position. They are able to achieve certain communicative goals despite their limited proficiency in the target language. Just as Carroll (2004) observed that Japanese novice speakers of English used similar ways to self-select as those of native speakers of English.

Based on an analysis of the 152 cases, we find three types of successful irregular self-selection in learners’ interactions: TI, TC, and THA. The TI occupies the most of them (63.4%), reflecting that Chinese postgraduate EFL learners are used to interrupt to obtain the speakership in conversations. Park and Duey (2020) also found that in multi-party workplace meetings, the self-selector interrupted to gain the speakership. This indicates that interruption is an important device for both native and non-native speakers to participate in conversations. The TC occupies 24.2%, which is the second most frequent way of irregular self-selection mentioned as “floor-taking overlap” and studied by Konakahara (2015, 2020) in casual ELF conversations. The result reveals that turn competition is also a way of active involvement in non-native speakers’ conversation. The third one is THA, which only occupies 6.5%, but it also implies that learners are eager to participate in interaction by aborting the holding of speakers’ turn. They aim to claim the speakership and boost conversational development.

Referring to the underlying reasons for the initiation of irregular self-selection, we think it may be because learners in peer interaction are naturally in an equal position; thus, they will initiate and participate in a conversation more actively. Moreover, when hearers have relevant conversational knowledge, they will initiate irregular self-selection in different interactional contexts to show their “knowledge status” (Heritage, 2013, p. 376) and willingness to express opinions concerning a certain domain of knowledge. The findings of this study show that after a long period of English learning, about 10 years, Chinese postgraduate EFL learners have got certain self-selection capabilities and turn controlling awareness to obtain speakership, as shown in the aforementioned three cases. However, the initiation of irregular self-selection is also based on the flow of conversation, being context-dependent and unpredictable. On the one hand, the initiation of it is context-dependent (Lerner, 2003), which requires both participants to construct the context together. They work to promote the flow of conversation and to provide a self-selection context at the same time. For example, in excerpt 1, L can implement irregular self-selection because of the existence of a “rubbish sorting” context. On the other hand, the initiation of this kind of action is unpredictable, and the decision-making is in the hands of the hearer. For example, hearers’ participation state, knowledge state, emotional state, and individual differences will all affect their implementation of self-selection actions. Thus, only when learners have a certain awareness of turn monitoring can they initiate this action in a conversation.

Second, regarding how to self-select, by detailed analysis of the three cases, we find that (1) in lexical and syntactic dimension (i.e., verbal aspect), learners can provide appropriate language resources to participate in conversation according to the flow of conversation. (2) in prosodic dimension (i.e., vocal aspect), pitch reset and intensity enhancement are used by learners to different degrees. Some of them use both ways to increase the volume of their self-selection words, for example in excerpt 1, while others only use pitch reset or intensity enhancement to achieve self-selection, as shown in excerpts 2 and 3. (3) in non-verbal dimension or aspect, except in excerpt 1, L uses four kinds of non-verbal resources in her irregular self-selection, including gaze, gesture, head movement, and body posture. The other two hearers only use gaze to predict and achieve self-selection. From the use of multimodal resources, it can be seen that the learners in these cases use at least three kinds of resources to implement irregular self-selection, which shows their ability to use multimodal resources to some extent.

However, through the overall investigation of irregular self-selection that occurred in our data, we find that about 80% of the self-selectors only use one or two kinds of body movements to project or implement irregular self-selection, such as gaze, gesture, or head movement. However, other body movement resources rarely occur, such as facial expression and body posture. Lee (2017) found that learners utilized an ensemble of talk, gaze, gesture, and bodily orientation to gain the speakership. Konakahara (2020) also found that in overlap sequences, interactants collaboratively exploited multiple non-verbal resources, such as gaze, posture, and gesture, for organizing turn-taking and conveying meaning. Compared with these two findings, the overall modal complexity and diversity of Chinese postgraduate EFL learners are low, causing their behaviors to be restrained and inactive. This phenomenon may be related to the Chinese culture emphasizing introversion and restraint of conversation participation. It reflects that culture has a profound influence on one’s behavior, even when they use other languages to communicate and participate in the interaction. However, some studies show that in Mandarin Chinese talk-in-interaction, participants will use plenty of multimodal resources to take turns or manage their affiliation (Yang, 2007, 2011). For example, Yang (2007) found that Chinese speakers used non-verbal resources to manage turns, such as hand drop, gaze, non-gaze, touch, thinking face, and finger count. The result was not the same as found in the present study. Maybe another possible reason for the low diversity of body movements in this study is that learners are aware that they and their conversations are being recorded. Thus, they cannot behave naturally when using the English language to talk and tend to control and restrain their behaviors to some extent.

Last, regarding why to self-selection, irregular self-selection can be divided into six types: displaying knowledge (53.1%), aiding (12.6%), information request (11.2%), agreement (10.5%), cooperative completion (7.7%), and clarification (4.9%). It can be seen that the main purpose of irregular self-selection is to display knowledge, also mentioned as one of the self-selection purposes by Waring (2011). The result indicates that the hearer contributes to topical development by displaying his/her own thoughts and views or providing comments and new information (for example, in excerpt 3). Then, the purposes of aiding, agreement, and cooperative completion occupy 29.4%, used to support the current speaker in the meaning-making process. They also help to maintain the rhythm or pace of the conversation by showing listenership, understanding, active participation, and agreement (see Murata, 1994; Lerner, 2002) (for example in excerpts 1 and 2). Finally, the purposes of information request and clarification occupy 16.1%, used to interact with the speaker of vague information and to elicit further information. They also serve to show high interactional sensitivity and active participation (Konakahara, 2020).

Although the purposes of irregular self-selection are various, showing different communication intentions of the learner, the common characteristic of them is that they reveal the learners’ active involvement in interaction (Cogo and Dewey, 2012), topical development, and interactive sensitivity of conversation. Moreover, with irregular self-selection, the participants cooperatively move the talk forward, reflecting their cooperative communication intention. Konakahara (2015) obtained the same finding in casual ELF conversations of the overlapping questions. Consequently, non-native speakers are successful in “achieving mutual understanding and developing interpersonal relationships” (Konakahara, 2015, p. 37).

## Conclusion

This study investigated when, how, and why Chinese postgraduate EFL learners implement irregular self-selection from the multimodal interaction perspective. By providing descriptive statistics and using a multimodal conversation analytic approach to examine three excerpts in detail, the results show that learners are interactionally competent members to participate in the conversation. They are able to achieve communicative goals, but their body movements lack diversity as compared with other non-native English speakers, causing behaviors to be constrained and inactive.

Based on the findings, this study provides some implications for EFL learners, especially other East Asian EFL learners who are commonly characterized as silent, reserved, and inactive during discussions, particularly in the classroom. This study shows that EFL learners with high language proficiency will benefit from peer interaction to develop their interactional competence, as evidenced by the initiation of irregular self-selection and active involvement in participation. Thus, in oral English learning and teaching, more high-level peer interaction without teacher involvement should be carried out. Although irregular self-selection violates the turn-taking system, it is harmless to the topical development. Therefore, learners should be encouraged to use this kind of turn-taking way to participate in the conversation, making their interaction more natural and vivid. However, when participating in interactions, EFL learners need to pay much attention to the use of multimodal resources, especially a variety of body movements, such as facial expression, gesture, head movement, and body posture. The use of these resources can improve the diversity of body movements and enhance interactional ability with native or other non-native English speakers.

This study adds to the scarce research on EFL learners’ irregular turn-taking practices and the growing literature on the use of multimodal resources in their interactions. At the same time, it verifies the applicability of the multimodal CA approach to the studies of learners’ conversation again. It is of significance in the detailed investigation of the learners’ turn management and their embodied participation in the conversation. It helps to understand the visible processes through which learners positively claim the speakership to participate in the conversation and build a cooperative relationship. It has also provided new empirical evidence to confirm the fact that EFL learners are interactionally competent members to successfully participate in the interaction, although with limited proficiency in the target language.

Despite its significance, the potential limitation of a single-case analysis is that it can only be representative of the analyzed phenomenon. To gain a richer and more comprehensive understanding of the phenomenon of interest, more investigations are needed. Our study also suggests directions for future research. Although topic discussion is one of the most efficient and natural ways to collect participants’ interactional data, it would be beneficial for future research to investigate irregular self-selection in varied tasks, for instance, role-play games, jigsaw puzzles, quiz games, and so on. Moreover, as the conversations of the present study were collected between friends, it is worth exploring whether the observation also applies to conversations between participants who are not familiar with each other or participants of unequal power relations.

## Data Availability Statement

The original contributions presented in the study are included in the article/Supplementary Material, further inquiries can be directed to the corresponding author/s.

## Ethics Statement

The studies involving human participants were reviewed and approved by the Professor Committee of School of Foreign Languages, Northeast Normal University. The patients/participants provided their written informed consent to participate in this study. Written informed consent was obtained from the individual(s) for the publication of any potentially identifiable images or data included in this article.

## Author Contributions

MMJ contributed to the conception and design of the study, data collection, data analysis and interpretation, writing, and developing the manuscript. HPZ was responsible for data analysis and interpretation, manuscript development, writing, and editing. Both authors contributed to the article and approved the submitted version.

## Conflict of Interest

The authors declare that the research was conducted in the absence of any commercial or financial relationships that could be construed as a potential conflict of interest.

## Publisher’s Note

All claims expressed in this article are solely those of the authors and do not necessarily represent those of their affiliated organizations, or those of the publisher, the editors and the reviewers. Any product that may be evaluated in this article, or claim that may be made by its manufacturer, is not guaranteed or endorsed by the publisher.

## Appendix

### Transcription Conventions

(.) micro pause (0.3) pauses of 0.3 s [] overlap
: prolongation or stretching of the sound = latching
°° the word is markedly quiet or soft . falling intonation
away gaze away at gaze at
* stroke of gesticulation -. recovery of gesticulation
F forward movement H home position
----- close dashes indicate the holding of the body movements
