# Supplementary material for: Multimodal Irregular Self-Selection in Chinese Postgraduate English as a Foreign Language Learners’ Conversation: When, How, and Why
Source: Front Psychol. 2022 Mar 25;13:788438. doi: 10.3389/fpsyg.2022.788438 (PMC8990892; doi:10.3389/fpsyg.2022.788438)
Supplement: Supplementary file 3 [file Data_Sheet_1.zip › Transcribed data/Group 1.docx]

***Supplementary Material***

**speaker# Li**

- Hum(1.5)when uh it comes to the future(0.5) there are many changes(0.5) there are both good and bad sides(1.7) the weather become colder[hum] and Changchun will be heated(crack). I think at that time the air condition is worse than that of now(1.5), do you think(0.5)uh what the weather problem in the future(1.2)[hum?] and all what are the changes.

**speaker# Wang + speaker# Li**

- **1:** (1.0)Yes there are many environmental problems, uh(0.4)and now we are facing many(0.5)you know problem [of pollution]
  **2:** [of pollution]yes

**speaker# Wang**

- Such as i see the rain pollution and climate change, you know climate change is a serious problem we are facing now,uh cause if we don’t take many effective measures, uh it will cause disastrous consequences(0.9), you know the melting glaciers will cause the sea level to rise and the residents have to be accept the relocation and it also will cause more uh() on government, and the climate change also(0.6)have many great effects on hum animals[yes], uh it will cause many animals to be homeless.

**speaker# Li**

- Fortunately that the international community has has taken many effective measures to address this problem except the US, and we know(0.6)it has exceeded from environmental environmental protection organizations and(0.8)uh the weather's become colder it painful for me to get early in the morning to have class.

**speaker# Wang**

- Me too

**speaker# Li**

- (0.8)It's will be nice to have class at home

**speaker# Wang**

- (1.3)of course I agree with you. uh I really like to study at home you know with the development of science and technology, having classes at home became right more popular and I believe that one day uh students can have breakfast at home and listen their teacher at the same time. uh But you know this kind of classes maybe not effective, cause I don't like this kind of class, because I can(0.3) get distracted occasionally, but i think if we can work at home in the future , It maybe I will maybe feel more comfortable uh cause if there is leader standing there supervise me, it will I think I will be not uh productive(0.7),hum but uh you know actually uh if we can work at home maybe we will be more comfortable(0.7). and you know this morning I read a news about that there's a general election will be have in the united stated, so if do you think the uh trump will be the president in the future?

**speaker# Li**

- (0.4)I think Donald Trump have good chance of being elected I think he is not as simple as we thought hum he has done many ridiculous ridiculous things, we always read some interesting news about him, and he is reknowed as Dongwang in Chinese[yes]. actually I think he is very uh ambitious, uh for example he uh called on the American enterprises(0.9)uh go to go back to the United States which is the unemployment and in his recent campaign speech, he he spoke spoke of China for(0.5) more than ten times[yes], as we know with the development of China, the USA the people in the USA become more hostile to ourselves, I think what uh what he do is more than to hum more to(0.6) make American people(0.8)to focus on on us[hum yes]. never mind[hum], we don't talk about these national affairs, let talk about hum talk about ourselves[ok], what do you want to do in the future?

**speaker# Wang**

- (0.6)hum about the future I want to be an interpreter. uh cause when uh when I was a child, my father uh took me to a restaurant, and there a(0.6) group of Russian tourists hum I found several boys and girls, uh they invite me, uh so I think that's will be a good chance for me to practice my spoken English, uh so I use some English sentences, you know how old are you(laughter), or hum where you from come from that's

**speaker# Li**

- or what's your name

**speaker# Wang**

- Yes it's very simple sentences but it give me a good opportunity, so I can know how to use the correct English sentences or to uh chat with other foreigners. and after that, the restaurant staff praise me uh she said, wow, you are a little girl, but you have a very good uh spoken English, and i can see you are so brave and from that, I think it's my maybe it's my dream to be an interpreter and in the college, I choose uh English as my major uh cause I think if I can have a systematic uh study in the university maybe I will become it will help me to become a qualified interpreter, but I know there is a long way to go before being a qualified interpreter. I will try my best and work hard and never give up so how about you? what you want to be in the future.

**speaker# Li**

- You are very social and good at interpreting[yes yes]. I think you are suitable for being an interpreter, and I think NENU will provide a good platform[yes] to be an interpreter, for me actually I uh I think become a teacher

**speaker# Wang**

- Wow that's a great job

**speaker# Li**

- Because my grand grand father is a teacher, my grand father is a teacher, my uncle is a teacher, they always tell me about uh about their students in the uh in the schools, uh I think they are very funny and the things are very interesting. hum besides I really really like the long vacations in winter and summer I think

**speaker# Wang**

- Yes you can enjoy yourself

**speaker# Li**

- Yes I can do anything I like, but you wonder you wonder that why I choose the interpreting as my major in my postgraduate period uh actually, I think teacher and teaching students is my career choice, but interpreting is my dream(1.1). I will use my spare time to practice my interpreting(0.9),hum one day, if I think I'm qualified as the interpreter I will take interpreting uh interpreter as my lifelong career

**speaker# Wang**

- (0.9)wow that's wonderful

**speaker# Li**

- Do you have any uh expectations in the future?

**speaker# Wang**

- (0.7)uh yes I hope that one day, I can buy a house by my effort and hum my parents would not worry about me and I can also hum take they take them to uh where ever they want I will you know creat a lovely family, I will have a(0.9)have handsome husband and a lovely child(0.9) may be two or three children[yes], and hum I think hum I won't to be worry about the money you know maybe I have a(0.5)high uh I will have many money(laughter)I think so and I can hum go where I want eat what I want and get what I want hum may be my future(0.4)uh

**speaker# Li + speaker# Wang**

- **1:** will be very bri [brillant]
  **2:** [brillant]

**speaker# Wang**

- Yes

**speaker# Li**

- Brillant

**speaker# Wang**

- I will so expect and you how about you, what is your expectations in the future?

**speaker# Li**

- what I want very very simple, I just want to have a big house[wow], a beautiful car and a handsome husband.

**speaker# Wang**

- I want to be your neighborhood

**speaker# Li**

- hum and I think uh the(1.0)hum and I think(1.0)the real state prices in our country will be lower, it's too hard for us to buy a house[yes], hum and if one day the real state price(1.2) will uh will very very inexpensive[yes], I will buy(0.8)hum a very very big house and at that house I I can always hold very(0.8)I can hold many party parties[yes]many times(1.0)hum

**speaker# Wang**

- There must be a garden in your(0.4)home.

**speaker# Li**

- Hum Yes I will buy a big castle

**speaker# Wang**

- Yes(3.1)and uh(laughter) actually uh I want to know uh if you will have a pet in the future, you know we are student and we don't have uh time or money hum to have our own pets, whatever a dog, a cat or pig, you know[hum], so do you want to have a pet in the future.

**speaker# Li**

- yes I think I will have a pet maybe a dog. I think it is very honest to us, uh it help me do many things, if I uh if I have a pet I have a dog in the in a very large physical form(0.7),hum I can let him to help me to carry somethings heavily

**speaker# Wang**

- (0.7)Wow hum and uh what's the name will you give to that pet

**speaker# Li**

- Maybe Jack(laughter)

**speaker# Wang**

- Jack It's a good name(laughter). Actually about me I want to uh have a cat you know cat it's uh do not need me to bring it outside, uh it can stay at home very uh(0.8)uh quite and uh uh but you know I need to take care of it, uh I hope that I will have time to take care of it cause uh it's not a cat for me uh maybe uh I need a friend, you know, uh in the future, maybe I uh will spend a lot of time on my work, and social with other people, you know people is very(0.5)(crack) complex, and maybe I will have the uh caurage to uh talk someone else uh we are not familiar with that uh uh what happened to me maybe someday I will be very sad, I need to talk someone, but I do not know uh strangers will have the patient to listen too me what happened, uh so maybe cat will give me uh

**speaker# Li**

- A sense of safety

**speaker# Wang**

- Safety Yes and I can talk to uh with it, uh never mind uh if it uh it can't speak to me I know but uh I will really happy uh it can accompany with me.

**speaker# Li**

- (0.9)hum Do you have anything to say to(0.7)uh to you in the future(0.4)ten years?

**speaker# Wang + speaker# Li**

- **1:** (0.7)Wow Ten years is a long term and uh maybe I will become a(0.6)33 woman[woman]
  **2:** [you'll]became an excellent interpreter maybe

**speaker# Wang**

- Maybe it's my dream, I really hope that will become true. uh but uh now I think ten years past(0.3), I will(0.9) become an independent woman[hum], I may have some uh some money, and I have uh I have been to many famous cities uh like uh(0.4) France, or England or New York hum(0.3)every uh many many places, and uh I will(0.5)uh I hope that I can uh spend time with my parents, you know ten after ten years, they maybe uh very old and I hope that they uh we can have time to sit there to chat each other, and know what happened to them, and they know what happened to me(0.4), and I hope that uh we can not(0.3)uh(0.6) worry about our house, you know house is a very important to us. I hope my parents will be uh will be uh very uh healthy and so that I can bring them uh take them to many places they want, and eat what they want. maybe ten years ah is a long term, I hope hum what I want would come true. so how about you.

**speaker# Li + speaker# Wang**

- **1:** hum I think I will run(0.4)I will run an interpreting company, I want to(0.3)cultivate hum(1.1) many qualified interpreter(1.3), also I think I'm not qualified(1.7)[hum]
  **2:** [but]after ten years will be(laughter). You'll be a qualified interpreter

**speaker# Li**

- Thank you
