# Supplementary material for: Multimodal Irregular Self-Selection in Chinese Postgraduate English as a Foreign Language Learners’ Conversation: When, How, and Why
Source: Front Psychol. 2022 Mar 25;13:788438. doi: 10.3389/fpsyg.2022.788438 (PMC8990892; doi:10.3389/fpsyg.2022.788438)
Supplement: Supplementary file 3 [file Data_Sheet_1.zip › Transcribed data/Group 10.docx]

***Supplementary Material***

**speaker# Fu**

- I am so excited

**speaker# Luo**

- (0.9)hum why are you so excited?

**speaker# Fu**

- Because the national day and the mid-autumn festival are around the corner. That means we are going to have holidays.

**speaker# Luo**

- Absolutely. hum they are both of them are Chinese festivals and Which traditional Chinese festivals do you like best?

**speaker# Fu**

- Well in my opinion, traditional Chinese festivals are significant for all Chinese people. As for me, I like spring festival best.

**speaker# Luo**

- Why do you like spring festival.

**speaker# Fu**

- There are many reasons first(2.1) I like spring festival because hum there are many uh interesting celebrations. And it's also a time for family reunion[yes].No matter where you are and what you do, you are expected to return home to spend time with your family members, and I like spending time with my families.

**speaker# Luo**

- hum Yes and do you know some other celebrations of spring festival?

**speaker# Fu**

- Yeah people uh people will set firecrackers and fireworks on the spring festival to drive evil spirits away.

**speaker# Luo**

- Yeah that’s right(0.5)and hum people will also pay visits to their relatives.

**speaker# Fu**

- Very true. uh It is called paying new year visit. hum And people will take the opportunity to exchange good wishes to their families, friends, (relatives) and teachers[hum]. and Like the wishes include wishing your work achieve smooth progress, wish you prosperity, hum whish you uh everything goes well and so on.

**speaker# Luo + speaker# Fu**

- **1:** Yes[and]
  **2:** [and]yes hum and hum people will also send their wishes from internet

**speaker# Luo**

- (0.6)Yes that’s right. And do you know some other customes of spring festival?

**speaker# Fu**

- Yeah like the elder will give red envelop to the children and it is called the luck money. The money will be wrapped in a red envelope.

**speaker# Luo**

- Yes. And hum Spring festival is almost the most important festival to our Chinese people, and we will get one year older hum after the spring festival.

**speaker# Fu**

- I deeply agree. And people attach great importance to this festival. hum For instance people will paste the spring couplets on the both sides of door and people will also paste a reversed Chinese character “福” mean symbolizing the coming of happiness and auspiciousness. What’s more, people will eat dumplings in the northern china.

**speaker# Luo**

- Yes and there are some differences between the south and the north.

**speaker# Fu**

- Absolutely in the northern china, people eat dumplings, while in the southern china, people eat niangao[hum].

**speaker# Luo**

- Yes and hum it is a uh it is a very important festival and it is also the beginning of the new year.

**speaker# Fu**

- I deeply agree. uh I think almost all Chinese people are expected the arrival of the spring festival. It means a lot to Chinese people.

**speaker# Luo**

- Yes

**speaker# Fu**

- hum What’s your favorite traditional Chinese festival？

**speaker# Luo**

- hum My favorite festival is the mid-autumn festival.

**speaker# Fu**

- Why do you like the mid-autumn festival?

**speaker# Luo**

- Because I think the mooncakes are delicious.

**speaker# Fu**

- (0.4)I deeply agree. I like to eat mooncake uh I also like to eat mooncakes I think they are really delicious.

**speaker# Luo**

- Yes And There are also some hum customs. hum For example, people will hum watch the moon because on that day, the moon is very big and round.

**speaker# Fu**

- I agree the shape of the moon is like the shape of mooncake.

**speaker# Luo**

- Yes absolutely. And hum on the mid On the mid-autumn day, people will people will also uh get together it is a festival for family reunion. And they will have a big feast.

**speaker# Fu**

- Definitely. people Families will sit together to have a family reunion feast to celebrate the festival.

**speaker# Luo**

- Yes

**speaker# Fu**

- And I think people will appreciate the moon while eating mooncakes.

**speaker# Luo**

- Yes it's very nice.

**speaker# Fu**

- I think so hum So Can you tell me what’s the legend about the festival I want to know.

**speaker# Luo**

- Yes. hum I know one version, it is related to(0.5)Change and Houyi.hum Change is uh the wife of Houyi[uh]

**speaker# Fu**

- [They are]a couple

**speaker# Fu**

- I truly like this story, it sad but uh fascinating I think.

**speaker# Luo**

- Yes and I think the mid-autumn festival is also very special. hum because some In ancient times, some poets will uh some poets wrote some uh poems related to the moon.

**speaker# Fu**

- I agree. I I know a poet named sushi, she uh he is from song dynasty.

**speaker# Luo**

- Yes(0.8)yes and uh the poems related to the moon hum are uh most of them are used to express the their miss to their families.

**speaker# Fu**

- (0.8)hum Yes

**speaker# Luo**

- Yes and which festivals do you uh and what festivals do you like(0.9)besides the spring festival.

**speaker# Fu**

- Except the spring festival, I think I like the dragon-boat festival, it is also called double fifth festival.

**speaker# Luo**

- oh I know I know it but why do you like the dragon-boat festival?

**speaker# Fu**

- (0.7)uh personally I think uh(0.8)dragon-boat festival uh provide us with an opportunity to eat glutinous rice, I think it’s delicious.

**speaker# Luo**

- (0.4)hum Yes and it is often wrapped uh by the bamboo leaves. And do you know some other customs of the dragon-boat festival?

**speaker# Fu**

- Yeah I think people will hold uh the dragon boat race on the festival to celebrate it.

**speaker# Luo**

- Yes uh hum but in northern china, people will not have such a race, so I haven’t seen it yet.

**speaker# Luo**

- hum do you know some hum do you know the legend of the dragon-boat festival?

**speaker# Fu**

- (1.4)the It is said that the origin of the dragon-boat festival is to commemorate the patriotic poet Quyun. Quyun is from chu people(1.3). He is noble and kind(0.6)and he was jealous(1.6) by others.

**speaker# Luo**

- hum Yes I know this story we have learned it in the textbook. hum the monarch didn't trust Quyuan[hum],uh so he was very disappointed so he committed a suicide. And uh He wrote the hum great work Lisao hum to express his feeling.

**speaker# Fu**

- (0.6)Yes I think Quyuan is really a great poet in Chinese history, he uh committed suicide to testify his innocence.

**speaker# Luo**

- Yes that’s right[hum] and I think people all loved him very much.

**speaker# Fu**

- hum Do you like the dragon-boat festival?

**speaker# Luo**

- Yes I like it very much.

**speaker# Fu**

- Why

**speaker# Luo**

- hum Because uh I like the uh food(0.4)I think

**speaker# Fu**

- you mean uh you mean the glutinous rice

**speaker# Luo**

- Yes I think all these traditional food are very delicious. And I was moved by the story that people just hum threw food into the river to keep fish from eating Quyuan’s body.

**speaker# Fu**

- Yes hum Quyaun was uh Quyaun was appreciated by the local people. So[/yes] people wanted to protect him.

**speaker# Luo**

- hum Yes that’s right[hum]

**speaker# Fu**

- What other festivals do you like?

**speaker# Luo**

- hum I like the double seventh festival. It’s also called “QI Xi” festival in Chinese.

**speaker# Fu**

- Oh I see. Why do you like it?

**speaker# Luo**

- hum I was uh because I uh like the story related to it.

**speaker# Fu**

- What story

**speaker# Luo**

- Yes It's about niulang and zhinv uh

**speaker# Fu**

- Can you tell me who is Niu Lang and who is Zhi Nv

**speaker# Luo**

- Ok. hum I’d like to. hum Zhinv is the daughter of the queen in the heaven, and hum niulang is a cowerherd. hum but He is just a normal people hum So her parents don't agree with this marriage, so they just called back Zhi Nv into the heaven so they two were seperated. But They have uh they only they can meet each other only once a year. it is on the seventh day of the seventh lunar month. So that’s the myth of double seven festival hum

**speaker# Fu**

- uh That’s is truly a fascinating story.

**speaker# Luo**

- Yes hum and people uh it is called the Chinese valentine’s day. and I think uh it is also uh meaningful because we have our own traditional festivals and we shouldn't uh abandon this festival.

**speaker# Fu**

- (1.3)I think so. I agree with you.

**speaker# Luo**

- (2.3)hum yes and hum I today I'm very happy to talk with you[we]

**speaker# Fu**

- [Me] too. I think I have learned a lot from you. I know the traditions uh I know the customs and origins of the double seventh festival and mid-autumn festival. and I think today I learned a lot.

**speaker# Luo**

- Yes I also learned something about the spring festival and as well as the dragon boat festival. and I hope we can spend these festivals together in the next three years.

**speaker# Fu**

- Good idea. I anticipate it.
