# Supplementary material for: Multimodal Irregular Self-Selection in Chinese Postgraduate English as a Foreign Language Learners’ Conversation: When, How, and Why
Source: Front Psychol. 2022 Mar 25;13:788438. doi: 10.3389/fpsyg.2022.788438 (PMC8990892; doi:10.3389/fpsyg.2022.788438)
Supplement: Supplementary file 3 [file Data_Sheet_1.zip › Transcribed data/Group 11.docx]

***Supplementary Material***

**speaker# Wang**

- How is it going?

**speaker# Xu**

- (0.3)hum Recently I have been confused(1.2)yeah I'm confused.

**speaker# Wang**

- Confused? what[/yes] are you confused about?

**speaker# Xu**

- (0.6)hum I'm a little confused about my(0.5)employment.

**speaker# Wang**

- You mean your future job?

**speaker# Xu**

- Yes[hum]hum although we are in the first year of post graduate hum you know time will(1.6)[hum]time will

**speaker# Wang**

- Yeah time really flies.

**speaker# Xu**

- Yes so uh I think it is essential to consider on employment.

**speaker# Wang**

- hum

**speaker# Xu**

- hum but before that topic I want to know why hum why you want to uh(0.9)go on uh

**speaker# Wang**

- Yeah go on my acdemic study? here?(0.6)Ok hum(0.6)I mean when I was a freshman[yes]in the college I decided to be a postgraduate. hum I don't know, just when I was in high school my teacher just tell us that uh if you have the opportunity to further your education in in the future, you need to seize the opportunity and continue your study. uh So just in during my high school, I know that uh if I have the ability to be a postgraduate, I should try I should have a try[yeah]. So when I enter in the college, uh I just study hard, and uh everything I do is kind of for my uh further education. hum Now I'm a postgraduate and I think it's a good choice uh[/yes] to further my education[yeah]. yeah and What about you?

**speaker# Xu**

- hum My story is a little funny. Because in my uh first third year I never consider to receive further education.

**speaker# Wang**

- You never? [you never]

**speaker# Xu**

- [yes]yes in the last year suddenly I naturally prepare for my postgraduate examination.

**speaker# Wang**

- I mean Without any reason? without any force? you just you[/yeah] suddenly?

**speaker# Xu**

- (0.4)Yeah maybe in order to escape employment[hum] at that time I have no idea what should I[/hum] hum I'm busy on which kind of career[hum]I don't know hum I like uh which kind of career[hum] So hum I started to prepare for my entrance exam[hum]yes it's so ridicoulars so I think life is full of unpredicable choics[hum]yes.

**speaker# Wang**

- unpredicable Ok[yeah]hum I mean you mean your you suddenly thought about uh maybe it's a good choice to be a postgraduate[yeah]instead of uh finding a job, right?

**speaker# Xu**

- Yes

**speaker# Wang**

- uh actually I don't know. we nowadays college students are uh it is very hard for college students to find a I mean satisfying job[yeah]. hum I When I graduate from my college, uh I have uh I have enter I have attended a interview uh for a primary school teacher, hum I mean actually I wanted to be a maybe high school teacher or a jounior high school teacher but actually I mean the employers they think that I'm just qualified to be a primary school teacher[yes], not qualified to be a high school teacher. uh it's kind of frustrating, uh so I know that hum a higher dgree is very important, especially in China. in There are so times uh there are so many people with higher degrees[yes]. yeah so if I want to be uh maybe a higher a higher

**speaker# Xu**

- Higher skilled

**speaker# Wang**

- Yeah yeah highly-skilled teacher maybe I uh I have to(0.6)

**speaker# Xu**

- Work hard

**speaker# Wang**

- Yeah for futher my education to get a higher degree.

**speaker# Xu**

- Yeah So you are sure you will be a teachet in the future?

**speaker# Wang**

- uh truly

**speaker# Xu**

- You have made up your mind.

**speaker# Wang**

- uh It's hard to say that. hum I mean uh my family or uh people around me[yeah] uh they suggests that to be a teacher is a good choice for a I mean for a girl. uh it is stable it is

**speaker# Xu**

- Yes the same as my parents.

**speaker# Wang**

- Yeah uh especially I mean in Shandong Province[yeah], uh parents uh think that to be a teacher is a good choice[yeah], hum yeah they prefer a stable job[yeah]. hum [I]

**speaker# Xu**

- In the career is more suitable for girls that's right?

**speaker# Wang**

- Yeah this is as pation or to be a teacher is is a stable job and it is also uh I mean respected by the whole society[yes]. They I mean You will never lose your job as education is I mean education is forever.

**speaker# Xu**

- Yes[/yeah]and this position has many hum holidays.

**speaker# Wang**

- Holiday[yeah]

**speaker# Xu**

- [Summer] holiday and winter holiday.

**speaker# Wang**

- hum Winter holidays[yes]Yeah hum actually when I hum when I was a little girl[yeah]uh you know we usually play this game of role-play, I tend to play as a teacher, and I I mean it’s kind of imitation[yeah]. I just imitate my teacher to have class to teach(0.8)other younger kids than me. hum when I was a little girl I kind of enjoyed it(0.7), uh the feeling of be becoming a teacher. I think I mean I can uh ask them to do a lot of homework without any reason.

**speaker# Wang**

- So hum you mean What’s in your mind? Do you want to be teacher or another job?

**speaker# Xu**

- Teacher is not a is a not bad choice[hum].hum But I think in fact our major[hum]hum doesn't offer us so much choice. Maybe we can uh after graduation we can be a teacher[hum] or a translator[hum] interpreter[hum]. And maybe You can enter a company to uh engage with foreigners and connect with them[hum] to deal with international trade. But I think it uh a little challenging, because my spoken English is not good. So(0.8)there uh so Entering a company hum is not my is not what I want to pursue in the future. hum As far uh as I'm concerned, hum the teacher is so good but hum is not so interesting. and I pusue this position I think I don't have much expectations for my future.

**speaker# Wang**

- Hum I got it.

**speaker# Xu**

- Yes yes I think it's a little just a little hum boring. So(0.7)[hum]so I have no idea what I will do in the future. So I'm a little confused(0.6) yes

**speaker# Wang**

- hum Ok hum I mean I'm also(0.4)kind of aimless I don’t know what will I do in the future, maybe a teacher or hopefully, a translator in a(1.7)foreign company, but who knows, I mean uh just a few days ago[hum], I had a discussion with my tutor[yeah], she said that the only thing I need to do now uh is to seize today. seize today To do what uh to do the homework today, uh to learn as much as you can and to I mean to in the end you can write[/yes] a qualified thesis. uh So that you can uh graduate as a postgraduate with a higher degree. and She suggested that I should not choose to be I mean humIt is hard for me I mean I have little opportunity to choose what I uh I can be[yes] in fact, it’s the employers who choose us uh they think if we’re qualified to be a teacher, or to be an interpreter. uh So I after that I was clear of,hum kind of I suddenly understand that it is to early to think about my future career the only thing I need to do now is to study hard[yes], to seize today hum maybe in the future, I am qualified enough to be a translator or an interpreter. hum If there’s any opportunity I can seize it. uh but If unfortunately I'm not so qualified to be an interpreter oy translator uh maybe it is a good choice for me[/yes] to be an English teacher(0.8).uh I mean(0.7)uh We need to make a living yeah[yes], we need to make money uh so maybe it is a good choice.

**speaker# Xu**

- Yeah[hum]you know I think my biggest problem is to[hum]to think more but do less. That's my biggest problem[hum]. and I think hum we also need to study the professional skills[hum], meanwhile we should have a general plan[hum].I think if I have a general plan for future yeah I believe I have confidence to be qualified for hum what I uh want to pursue[hum]. Yes I think that will be good yeah

**speaker# Wang**

- hum Yeah I believe that uh if you you don't know what you want to be[yeah]in the future now just do what you need to do uh to maybe now I need to do my homework to tranlate uh to translate 500 I mean uh 500 words long texts[yes]hum Don’t you think that to be a translator is kind of difficult even though we are I mean we are MTI(0.8)[but]

**speaker# Xu**

- [There]are a few of English majors hum are hum can have their ability to pursue their position both translators and interpreters both difficult for us a little difficulte so if we want to be pusue this position we have the hum we need have more time to study and study hard.

**speaker# Wang**

- Yeah to study hard So maybe uh I mean sometimes I'm kind of uh worried uh about future, I mean although I work so hard maybe to be a qualified translator, but maybe in the end, I can’t. even pass The exam of uh catti level 2, I’m yeah when I though about it I was kind of uh frustrated but hum no matter what happened in the[/yes] end, I want to do my best for now[yeah] and just like during the preparation of the uh entrance exam for post graduate I you know it's a long process I

**speaker# Xu**

- Long process

**speaker# Wang**

- Yeah uh have you ever thought that you can(1.0)you can be admitted into this university?

**speaker# Xu**

- (1.5)Yes maybe may be

**speaker# Wang**

- May be uh I'm not so 100 percent sure uh so during that process I just(1.2)uh(0.7)I just told myself that to do what whatever I can do. hum if fortunately you can do it uh and now I make it I'm also a graduate now uh so I believe that maybe in the future I can there is a chance that I can be a translator uh but who knows.

**speaker# Xu**

- I believe you can.

**speaker# Wang**

- Well but actually it is hum it is difficut difficut[yeah]much more difficut than that I imagine yeah

**speaker# Xu**

- That's isn'y easier[hum] and After chatting with you[hum]my heart and my mood have lighten up. Thank you.

**speaker# Wang**

- Ok
