# Supplementary material for: Multimodal Irregular Self-Selection in Chinese Postgraduate English as a Foreign Language Learners’ Conversation: When, How, and Why
Source: Front Psychol. 2022 Mar 25;13:788438. doi: 10.3389/fpsyg.2022.788438 (PMC8990892; doi:10.3389/fpsyg.2022.788438)
Supplement: Supplementary file 3 [file Data_Sheet_1.zip › Transcribed data/Group 12.docx]

***Supplementary Material***

**speaker# Liu**

- So today our topic is how video games is affecting our our lives.

**speaker# Su**

- Yes

**speaker# Liu**

- (0.7)So I think(1.1)we are the generation that(1.6)we have the most to speak of when we come to this this topic. because We are like the first generation of fans of video game In China.

**speaker# Su**

- (1.1)Yes at first Can I ask you a question?

**speaker# Liu**

- Ah yes

**speaker# Su**

- Yeah Do you like play video games?

**speaker# Liu**

- Yes of course(0.3)What about you?

**speaker# Su**

- What what games

**speaker# Liu**

- (0.6)I play Dota for I play Dota for like ten years uh I’m still playing now. I know I know you play The Honor of Kings I don't know[how to say it]

**speaker# Su**

- [Yeah](1.3)why[/so] are you interesting I think it's a good game

**speaker# Liu**

- (1.0)Yeah of course it's interesting. I think the best part of it(0.5)is that you can compete with each others and it grants you some kind of fulfillment when you are trying to win especially when you are working as a team with your teammates.

**speaker# Su**

- Yes I think according to this game, we can form a good friendship with our hum game players[game player]

**speaker# Liu**

- [Yeah]Because after like uh so many years of you know schooling and education experience, you will find out that what matters the most are those guys that you played video games with many years ago. they are You know it’s a way of(0.6) developing friendship.

**speaker# Su**

- Yes(0.9)so[/uh] I think we don't we don't hum play these games often.

**speaker# Liu**

- Too often?

**speaker# Su**

- hum it has some bad effects to our life(1.5) [do you think so]

**speaker# Liu**

- [I think](0.6)You you mean the bad influence of video games that our parents told us I yes?

**speaker# Su**

- (0.6)hum Also it has some bad influence to our life.

**speaker# Liu**

- I think(0.9) In my opinion you know everything have a bad influence if you do it too much.

**speaker# Su**

- Yes

**speaker# Liu**

- Not Including video games. but I think uh we the education we received in our early age that our parents told us that uh video games were like monsters.

**speaker# Su**

- Yes

**speaker# Liu**

- Like monsters It was an slogan yeiled out by our government. I think you mean the influence of video games is exceedingly relative concept because uh it's like it was like just the policy in early age, when we were entering the WTO, and the Japanese group didn’t want us to be in that organization because we do a lot of copies of you know the discs of SONY company. That harmed their business interests so they refused us to enter the WTO. so As our response to this kind of uh political(0.8) bad will, we returned them with you know educating our kind that video games are monsters so we don’t buy their games. It was an you know prominent business for Japan so they lost uh Japanese they lost a lot of interests in Chinese market so they have to vote yes for China entering the WTO. So that’s the story.

**speaker# Su**

- I I think Although there are many restrictions hum for the teenagers but I think those restrictions hum doesn’t work very well(0.7)to you know for our teenagers

**speaker# Liu**

- You mean it's necessary you mean it's unnecessary you know

**speaker# Su**

- No I think it's necessary=

**speaker# Liu**

- =It's useless

**speaker# Su**

- hum yes but it's doesn’t work very well.

**speaker# Liu**

- I I think it doesn't have to exist(0.9). because everything has a reason. If your kind is fascinated by the video games, just let them be! I don't know what I don't know what's bad about it. because If he doesn’t play video games or something else, he will be addicted to something else something a lot worse something like drugs you know for American teenagers, mariguana a lot of things that have worse influence than video game. so I think these restrictions are necessary.(1.3)[It's why they are useless]

**speaker# Su**

- [Just because]Just because they are very young, so they don’t know how to control themselves that’s a[yeah] big problem.

**speaker# Liu**

- But you know if you lack if you are lack of self-restriction, you can make a lot of worse decisions than playing video games. So uh maybe maybe I think being addicted to video games is kind of a good choice for teenagers(1.0). That’s[/Ok] my real opinion.

**speaker# Su**

- Ok

**speaker# Liu**

- (1.4)you know After witnessing too many bad(0.7) bad kid like(0.9) they will(0.7) they will have sex everywhere. They will do drugs and bully[/yes] other people. so Comparing to these guys, I think those who spend their time in front of computers, in front of their phones they are relatively good.

**speaker# Su**

- Yes I I really agree with that part. With the popularity of video games, it has also created many new jobs for this society.

**speaker# Liu**

- Yeah

**speaker# Su**

- (0.4)[hum such as[/yeah] the many game anchors(1.3)主播. yes（1.3）they do some hum teenagers who are bad on therir studying(1.5).hum and they Because of this game, they have a job for their lives.

**speaker# Liu**

- (1.5)Yeah that’s a point.

**speaker# Su**

- Yes

**speaker# Liu**

- (0.9)hum You know somebody has to be good on something(1.1). You can’t be a all-time loser for your life. You must be good at one thing or two thing. Maybe they just find their own thing. you know video[games]

**speaker# Su**

- [yes]So the interesting is very(1.0)is very important

**speaker# Liu**

- You mean the interest in [video games]

**speaker# Su**

- [interest yes]

**speaker# Liu**

- (0.8)hum Maybe if they get in touch with some you know basketball players or uh other kind of influence, maybe they would end up playing basketball or soccer[/yes](1.0).Maybe this because(1.1)you know The computers are becoming common in Chinese homes and maybe this is like an easy access for the teenagers to get in touch with the Internet or computer games. so Maybe that’s why[hum]they got into the business.

**speaker# Su**

- I think some parents they don’t want their children to play with computer games just because hum they are very bad for their health the children's health but we if we are addicted to playing basketball that's different.

**speaker# Liu**

- (1.3)(laughter)but I think I am like a real-life example for what's you just said. I used to be addicted to basketball and I played it all the time. But look what basketball got me?(laughter) Bad knees. I can barely use bicycles and I can and I hurt my knees every time when I go up stairs or down stairs. so I don't think if you mean bad health you say you mean if they spend too much time watching the screens of computers or phones, they will have bad eyes . I think comparing to bad knees, I prefer bad eyes. Maybe if I got in touch with computers earlier in my age, that I didn't get to uh you know experience basketball hum maybe I will have a happier life(laughter).

**speaker# Su**

- Yes I Actually actually everything has these bad and good influences.

**speaker# Liu**

- (1.1)hum just like I said It’s a exceedingly relative[yeah]. You don’t say something is you know simply bad to someone. You just say under this kind of scenario specific scenario this can be(0.7)bad yeah.

**speaker# Su**

- (0.7)Yes I think so

**speaker# Liu**

- All right
