# Supplementary material for: Multimodal Irregular Self-Selection in Chinese Postgraduate English as a Foreign Language Learners’ Conversation: When, How, and Why
Source: Front Psychol. 2022 Mar 25;13:788438. doi: 10.3389/fpsyg.2022.788438 (PMC8990892; doi:10.3389/fpsyg.2022.788438)
Supplement: Supplementary file 3 [file Data_Sheet_1.zip › Transcribed data/Group 13.docx]

***Supplementary Material***

**speaker# Ma**

- Hum The topic we're going to talk about is teachers. Yes It's a(0.9) occupation that has highly respected by the people since ocean time ancient times. hum There are a lot of phrases for this occupation like hiding, integrating makes normal. And moral integrating makes a teacher. So hum what is there any teachers you like that has important influence on you?

**speaker# Xie**

- hum I want to talk about my uh Chinese teacher in my elementary school. uh He is a uh amicable but uh restrict a restrictive person. uh I first I want to talk about his amicable. uh She is usually care. She usually take good care of our students. uh When they go to see it. We usually can, uh he usually uh she usually uh take a good care of us. uh And if some students have their clothes broken uh he also neet the close for us. I think hum for the warm action that he converted to us uh had a important significant influence to me, uh so I uh also choose to be a teacher in my in future. And when talk about his strict, the aspect of strict, I think he usually is strict with our homework. and If we don't uh perform well he usually she usually do some small punishment to us uh then

**speaker# Ma**

- Yes I agree with you yes hum my(0.5)elementary school and in my high school, they are the Chinese teacher and the political teacher. yeah They have the same characteristics as you as a elementary school school's Chinese teacher, I think(0.7)they are both strict and amicable to us. hum During the class, we are very afraid of my teachers[yes], yeah he is very strict with our work, our performance on the class. hum So we have to(0.5)pay(0.3)all the attention to their class, able to perform well and get good grades. But after class, my teacher will accompany us to go back to my home and buy a lot of uh food and snacks for us to eat. I think that's really amicable and very makes me feel very comfortable[hum]. yeah hum So from this teachers what qualities do you think Should the teacher have?

**speaker# Xie**

- hum I think the teacher is a uh occupation. hum I think they have three qualities. I think it is more the most important. First is uh Professional knowledge. uh so the teacher act as the uh tutor of a student, he hum impart knowledge to the student. So hum the teacher must be specialized at what they're teaching. hum And the student can have a better understanding about the knowledge what they have learned. uh Second is the uh strong sense of responsibility. In school I think the teacher is not only act as the teacher, he you usually hum play a role in the student after class activities. hum They often take care uh every sides about students their life. So that must they should have a strong sense of responsibility. And the third uh the last quality I think they should have is the hum dedicated spirit. uh The teachers' hum work I think it is not only teaching. uh They also have some other uh attacks tasks, uh such as care for the student psychological uh health. hum When uh students have conflicts uh when students have conflicts with their classmates. So the teacher usually, hum the teacher should also uh act as a counselor to help them resolve the problem. hum That's all.

**speaker# Ma**

- Yeah hum I do think the teachers should have the following countries. First he or she should be very professional in the subject He or she is teaching. Because hum the children is It's like a white paper[hum], yeah what you keep what the teacher teaches him them is very important if the knowledge is wrong, yeah they are learning the false knowledge about the teacher.hum I think hum it's very important for teacher to master the professional knowledge. And secondly, I want to say yeah high sense of responsibility. the teacher are not teaching the students knowledge, but also teach them how to be a person also, how to cultivate their personalities how to deal with life outside the school yeah. And thirdly I think(0.3) the teacher must be patient because hum students are ridiculous, yeah they don't know how to control themselves. Sometimes they make mistakes Yes. hum as the teacher takes a very important part in their life almost hum(0.7)5 days a week in the school. So teachers(0.7) just like their mother their father. So we must have the patience like a parent(0.8) to help them solve their problems to correct their mistakes. Yeah That's that's hum just a three three aspects of counties[I think].

**speaker# Xie**

- [hum I think] the possible[yeah]their personality is far from what we have talked.

**speaker# Ma**

- Yeah yeah yeah hum(0.9)What we have talked about is very limited.

**speaker# Xie**

- hum Yeah

**speaker# Ma**

- (0.5)hum so(1.1)hum you know in the(0.6)news report there are many reports about(0.5)the teachers. Some of them are bad and some of them good. For example there are reports about the corporal

**speaker# Xie**

- (0.4) punishment

**speaker# Ma**

- punishment about uh to the students yeah that's really horrible I think. And there's some good news about teachers like Zhi Yue ying. yeah She's a teacher who have worked in a small village for 13 years to teach the students there[hum]. And what do you think of these teachers?

**speaker# Xie**

- wow I think this teacher played an example for us. and The teacher is very dedicated to his uh occupation and devoted his own time to educate the student. I think this is the this spirit uh require of us to learn. uh But when to talk about the corporate punishment, uh I think uh recent in just uh a few days ago I have read a news. hum The teacher find the student play card in the classroom, so after class the teacher called his mother to uh(0.5)hum educate the child. uh The children is in a middle school a middle school student hum(0.9)hum The mother after his mother come to the door of the come to the classroom, his mother claimed the boy hum(1.3)on his classmates(0.5).hum(0.6) the These conflicts last a few minutes. uh However the boy uh choose to plunge into the ground(0.8).hum(0.8)and uh Before he plunge into ground, he hesitated a few of(0.5)minutes. hum But uh what uh regret to us is it he died and hum after he was sending to the hospital, he uh didn't saved by the doctor. I think this is a sad story. uh I'm very heart broking. hum So I think this is also a lesson to us when the teacher uh dealt with the problems we should hum reflect reflect reflect.

**speaker# Ma**

- Yes I agree with you. I really shudder at this sad news Yeah. hum How terrible yeah teachers are important to students how can they do corporal punishment to the students. And(0.7)it makes me to think of the(1.0)current education system. yeah The colleges and universities should not only teach the students(0.9) their students the knowledge, but also(0.4)to help them in their morality yeah they should(0.5)let the university students to be an example in the occupation not a bad one(0.4). and I think I'm very pleased with the hum this kind of news like Zhi Yueyin yeah.hum the society needs more teachers like her yeah I think the country can improve our education system to enroll more(0.6)teachers with high quality to(1.5) enter the teaching system and maybe we can improve that pay and improve the threshold for the teachers[hum].yeah It's a very important point, because many people think the pay is very low for teachers. so high content and with high learning, person don't want to enter into the teaching system. We need to(1.9)change that yeah[/hum]the country should do actions to change that[hum]it's very important.hum Do you have any other place about this?

**speaker# Xie**

- (3.0)hum I want to talk about a story about my classmates. uh I graduated from a normal university this year, and I find my most of my classmates are going to uh going to be a teacher. hum and I I think I think uh one of my classmate perform well. uh I want to take him as an example. He also uh from Hunan[yeah] Hunan Huaihua but now he is a hum elementary school uh He is a math teacher in elementary school. hum And I uh I have watched an open class from his uh wechat moment. and Today I also uh find he post at exam examination papers that his students had made good grades[hum] after his only 1 month teaching work. I think he do really do an excellent hum he's very excellent.

**speaker# Ma**

- Yeah yeah hum That's ery fantastic[hum]. I hope your student your classmates can be a good teacher in the future[hum]. Then we talk about our impression on the occupation of teacher and talk about what qualities should teach have and our complains about the current(0.9)news[hum] on teachers I think is very meaningful[hum]. And it's a very precious talk with you

**speaker# Xie**

- hum We have a nice talking.

**speaker# Ma**

- Yeah[ok]. That's all. By
