# Supplementary material for: Multimodal Irregular Self-Selection in Chinese Postgraduate English as a Foreign Language Learners’ Conversation: When, How, and Why
Source: Front Psychol. 2022 Mar 25;13:788438. doi: 10.3389/fpsyg.2022.788438 (PMC8990892; doi:10.3389/fpsyg.2022.788438)
Supplement: Supplementary file 3 [file Data_Sheet_1.zip › Transcribed data/Group 14.docx]

***Supplementary Material***

**speaker# Zuo**

- hum Today uh I would like to uh give you uh some topics on the English learning, so I would like to ask you that if you can share some your experience on your English learning?

**speaker# Shao**

- (1.1)OK I can I can give you hum as a novice interpreter in Nenu and uh and it's my first time to be a post graduate, It's very new for me to uh experience the new life, and especially for my English Learning. hum First of all, I want to introduce my uh study experience on listening. hum as for me uh listening is not uh I'm not very good at listening hum so hum as a major in English interpreting, the most important part is to practice a lot in our listening, especially those note-taking skills, and short memory, and attention splitting[yeah] they're very important. hum But sometimes uh especially in our interpreters training campain uh camp. hum There is a hard time for me uh especially, when we do some on live interpretation and most time, uh sometimes I can not catch all the words for one time listening. hum so what about your uh listening study experience

**speaker# Zuo + speaker# Shao**

- **1:** (0.4)hum I think although my listening is not so good, but I think I can have some advises to you. hum I think just like my teacher said, as an interpreter, you have to have the habit of listening to uh various of news every day[yeah], such as VOA, BBC[yeah] or etc. and(0.6)uh besides the formal materials, you can also listen some informal materials such as the Allen Show[yeah] or Friends[this]
  **2:** [yeah]they are popular.

**speaker# Zuo**

- Yeah[yes] And hum apart from this, hum(0.7)I think the listening is an accumulating process[hum], you have uh to listening more, and keep it as a habit regularly, and so that you can hum sometimes accumulating, and form and it can release the power when you uh do interpreting tests, so hum although my English is not so good, because uh when I encounter some pronounce such as something about the astrology or some very unfamiliar words for me, I will also be confused, hum but I think for most of my interpreting materials hum as long as you keep up with the uh English news everyday, hum it’s not a big deal. uh so hum But my writing is really poor, so I would like to ask you some advice on writing learning.

**speaker# Shao**

- Ok hum Thank you for your advices on my listening, and I will follow it and try try to do something. and As for the writing, how to improve our writing ability and skills. hum First of all, I think hum input of some useful materials is really essential for us, hum because uh Chinese and English is really, are total different two languages, and when writing English passages or some kind of reports, we may find that's difficult. and At the moment, we should hum find some, uh borrow some books and materials from our laboratory and look into some useful materials, uh such as Business English, and some uh and some other special English. and(1.6)hum Here's what I have uh Here's all my suggestions.

**speaker# Zuo**

- Thank you I think one thing I should borrow from your suggestions is that uh indeed, I have uh the input materials of my writing is so poor, and I nearly hum read the reading materials or magazines for once a week, so I think it's so uh it's not enough for my English writing material[/yeah], hum so I think I should follow your advice and stick to it, and then we will see the uh a result.

**speaker# Shao**

- (0.6)Yep and above all and I also want have another suggestion that is to uh pay much attention on the grammar, uh because the grammar is the structure of our whole passage[yeah], and the grammar the important uh the correct grammar is really uh vital for our English writing[yeah]

**speaker# Zuo**

- [yeah]yeah yeah because when we speak English, we not pay much attention to the formal grammar[yeah], but in the writing English, we should pay much much attention to it. hum Ok, besides uh listening and writing, I think we also just as I mentioned, I read so little materials.

**speaker# Shao**

- Me too.

**speaker# Zuo**

- So so can we exchange some views about reading?

**speaker# Shao**

- Yeah we can. hum Recently, I just pay too much attention on my listening skills, uh to my listening and for some reading skill uh reading materials hum I also like to I really like the Economists, hum that is a very professional and outstanding materials for our English majors. and Anything else?

**speaker# Zuo**

- (0.7)hum

**speaker# Shao**

- Anything else?

**speaker# Zuo**

- I think we should all I think a magazine called New Yorker, or the Wall Street Journal

**speaker# Shao**

- Wall Street yeah

**speaker# Zuo**

- They are also very familiar[yeah] magazines. hum I think the Economists hum the [it]

**speaker# Shao**

- [Sometimes] it's too difficult for us[yeah].

**speaker# Zuo + speaker# Shao**

- **1:** [yeah]but it's very popular[yeah] in Chinese English learners[yeah]. uh Ok let’s talk about the last part hum that is [speaking]
  **2:** [speaking] Yeah

**(no speaker)**

**speaker# Zuo**

- Yeah So do you like to speak English?

**speaker# Shao**

- Yeah I really like speaking English, and I really admire uh those who can speak English very uh fluently, and uh just like the native speaker[yeah], and I prefer and I also prefer the American style spoken English. hum That's very natural and easy to understand[yeah]. Yep What about you?

**speaker# Zuo + speaker# Shao**

- **1:** I think I prefer the London accent[yeah], because so many people uh like the ()speech[yeah], because she speaks very well[yeah][English]
  **2:** [and]elegant

**speaker# Zuo**

- Yeah so hum I think speaking hum we can improve our speaking by reading some uh spoken materials and also follow the speeches of some famous people, such as the uh Evaca(0.4)[uh]

**speaker# Shao**

- [Evaca] yeah really goddess

**speaker# Zuo**

- Yes hum I think the(0.7)the most effective method is to practice more, uh no matter the listening, writing, speaking hum or reading. uh If you practice more and accumulate more materials, and you can improve a lot.

**speaker# Shao**

- Yeah Practice makes perfect!

**speaker# Zuo**

- Yes so hum OK thank you for joining me about the discussion of English learning, so uh nice to see you again, and have a nice week.

**speaker# Shao**

- hum Nice to see you again, and Looking forward to our next meeting!

**speaker# Zuo**

- Ok
