# Supplementary material for: Multimodal Irregular Self-Selection in Chinese Postgraduate English as a Foreign Language Learners’ Conversation: When, How, and Why
Source: Front Psychol. 2022 Mar 25;13:788438. doi: 10.3389/fpsyg.2022.788438 (PMC8990892; doi:10.3389/fpsyg.2022.788438)
Supplement: Supplementary file 3 [file Data_Sheet_1.zip › Transcribed data/Group 15.docx]

***Supplementary Material***

**speaker# Wang**

- So Today's topic is

**speaker# Ke**

- (0.6)hum today we are talking about an interesting topic and part time job[Yes](1.0)Y hum uh What's think about the part of the job? Why do you choose to part of job.

**speaker# Wang + speaker# Ke**

- **1:** uh Actually the latest part time job by two to this in the my preparation in the closer graduates uh entrance examination[hum]uh at that time, my a lot of people have persuaded me to put uh all my heart and mind into the study. uh All my friends and my parents always have the same attyitude. But I I know they were kind to me and really worry about me, uh but the reality is hum I was uh(0.6)under great pressure. hum I prepared exam alone. So I so I hum wanted some way to communicate with others. So I decided to(0.7)hum find a part time job uh as a teacher hum[/teacher] Yeah [yes]
  **2:** [I like]teacher

**speaker# Wang**

- hum As a teacher hum that's because students can uh learn English from me and the tuition phase wasn't high and the same time I can uh find ways to teach and talk and my living expenses will also be made up before(laguahter).So that's my yes So that's my experience. So what about you do hum you think is reasonable for the college students to take part time job uh during their college?

**speaker# Ke**

- (0.7)hum I maintain that it enables us to be more hum independent and the part time job can build up us(0.7)our can build up our self-confidence and it can uh give us the opportunity to get out of the every tower and to uh experience some(0.5) social abilities[yeah](1.0) however, as we all knowned our hum learning time is limited we have(0.6)much work to do every day. Y hum do you have many homework.

**speaker# Wang**

- (0.5)Yes My hum my college but homework in my college is hum(1.2)much(laughter)

**speaker# Ke**

- Do you accustomed to hum Brandon Mina oil?

**speaker# Wang**

- hum No I actually I won't I think hum uh(1.7)uh enough sleep(laughter) can make sure uh the quality of study and jobs. So I I wouldn't stay up late when I know there are many things to on the second day.

**speaker# Ke**

- I agree with you hum though there is last time we can hum the time just like the water in the sponge squeeze it and we can get more time. And our main task now is to learning, so we couldn't blindly to choose a cartoon job(1.0)hum it's necessary for us to take some real life hum real life social courses hum to(0.8)uh to experienced society and to hum get us(1.2)uh(1.3)we can touch with the outside world(2.9)。And it can help us students psychologically for the future work(1.5). Have you hum been to the job fair?

**speaker# Wang**

- (1.0)No actually I haven't.

**speaker# Ke**

- (laughter)hum I have been to the job fair and I found that now hum the HR are willing to choose those students with a lot of hum experience such as volunteer uh or uh guide。hum In this hum now the(1.0)uh part time job experience is as important as the the good Mark in our college.

**speaker# Wang**

- (0.7)In our degree(laughter).

**speaker# Ke**

- And can you(0.5)could you share us with your experience? Part time job experience.

**speaker# Wang**

- My reperience hum I still believe that the part time job, I have a lot of fun in part time job. hum The most impressive thing I have ever uh I made with a little boy a kid a boy.

**speaker# Ke**

- Little boy(laughter)

**speaker# Wang**

- Yeah he hum his English actually uh was not very good actually was bad hum he was always confused with the numbers in English. He was at his 4th grade but as he still uh couldn't point account the numbers from 1 to 10 in English(laughter). so At that time we did a lot of work. We uh read them we uh learned uh their songs and we recited them and dictating at the beginning of each class uh through so much hard work. hum He finally uh could hum recite it down uh fluently. I'm so proud of him. That's the most impressive thing. hum What about you do? Do you have anything want to share?

**speaker# Ke**

- uh I have take many partner jobs and uh my first part of the job is after the college entrance examination[hum].You know After the exam we feeled very relaxed and want to find something to do to experience their life[hum] and maybe hum to make a living[hum]to reduce the burden of our parents(0.7)。And Y guess what I choose for my first job, part time job。

**speaker# Wang**

- hum(0.8)Maybe uh maybe the same job with me?(laughter) A teacher?

**speaker# Ke**

- That's my second part time job. hum My first part time job is to hum to be a waitress in five star hotel.

**speaker# Ke**

- hum five star hoteluh When there is on wedding reception I serve I serving the guests[hum]. hum And usually before the hum the wedding reception, I always watching the splendid wedding(1.2). Have you imagine your wedding(0.5)wedding

**speaker# Wang + speaker# Ke**

- **1:** No I ha[dn't]
  **2:** [in the]future

**speaker# Wang**

- hum but I believe the wedding ceremony will be good and splendid。

**speaker# Ke**

- (laughter)hum uh my job is to hum sorting the guests to paly the dishes[hum] and(1.0)hum once I made a mistake.

**speaker# Wang**

- (0.7)What is it?

**speaker# Ke**

- I I spilt the soup on guests clothes.

**speaker# Wang**

- (0.3)oh that's

**speaker# Ke**

- Oh my goish. Can you understand how I scared when [at that time]

**speaker# Wang**

- [Did] Did the custom uh got mad with you

**speaker# Ke**

- (laughter)uh I apologize to him uh several times and he was very kind[yeah]. He said nothing(1.8).And but I was very embarrassed[hum](1.5). Overall I maintain that uh waitress is a very uh very hard work is very tired[yeah]. After the hum the guests have left. We must to uh clean up the dirty dishes and replace the new chopsticks and bowels[yeah]. and We always to on our face and we have no time to have a rest[yeah]. And after uh doing this waitress a few time I resolved I would never to do the uh service industry again?

**speaker# Wang**

- Because it's too tired.

**speaker# Ke**

- It's too tired and uh more importantly[hum], the salaries is very low.

**speaker# Wang**

- Really?

**speaker# Ke**

- Yes I only have ten RMB in 1 hour.

**speaker# Wang**

- (0.8)Ten RMB in 1 hour[yes]uh That's not high(laughter)

**speaker# Ke**

- hum Y uh what you learn about from your uh part time job experience.

**speaker# Wang**

- hum I what I learnt from part time job hum I think the most important thing uh I learned from part time job is uh when uh our teachers taught students at same time actually we learn from our student hum you know today's kids are very smart, they really cute and smart. hum Actually I I don't consider them cute at very beginning. I thought the kids are noisy. uh They were good at hum getting angry hum and they uh wouldn't fix their mind on their homework and wanted to play at any time. hum but actually uh But finally, uh when you became a teacher uh as though you will find as long as you can find the key to communicate with your students, you can actually make them cooperate with you. and They are really smart. That's my uh hum experience so do you have something to share.

**speaker# Ke**

- hum I choose to be a tutor[hum]hum

**speaker# Wang**

- and secondly as your second job

**speaker# Ke**

- hum and in the future[hum] I will constant continuously to do part time job[hum]hum to be a tutor[hum]. I in that tutor is the best job best part time job(0.8). It not only hum to get a lot of money, but also hum can but also we can learn a lot from the tutoring and is double payment. It not only requires the knowledge, but also requires the patients[hum]. and hum We can learn how to get along with the naughty children and to how to spare their learning interest. hum Maybe can hum through some videos or playing games with them. and hum through the tutoring not only can I transfer my knowledge from me to student but also hum uh transfer the happiness to the students[hum]. And so and because hum I will I want to be a teacher[and]

**speaker# Wang**

- [In]the future

**speaker# Ke**

- hum in future after my graduate study. So I I will constantly continuously to do hum to part time job to do a tutor(1.3). And uh I think teaching is the most brilliant career and ().

**speaker# Wang**

- hum Yes(2.7)hum So thank you for sharing your experience with me uh Catherine and it's nice to talk with you. Thank you. See you next time.
