# Supplementary material for: Multimodal Irregular Self-Selection in Chinese Postgraduate English as a Foreign Language Learners’ Conversation: When, How, and Why
Source: Front Psychol. 2022 Mar 25;13:788438. doi: 10.3389/fpsyg.2022.788438 (PMC8990892; doi:10.3389/fpsyg.2022.788438)
Supplement: Supplementary file 3 [file Data_Sheet_1.zip › Transcribed data/Group 16.docx]

***Supplementary Material***

**speaker# Liu**

- So *:* today we are going to talk about marriage.

**speaker# Song**

- Yeah marriage

**speaker# Liu**

- (0.3)As far as >I know< you have a boyfriend right?

**speaker# Song**

- Yeah

**speaker# Liu**

- So do you guys have like (0.4)a future plan of(0.4) like getting engaged with each other or even get married with each other?

**speaker# Song**

- (0.9)hum No I don’t think(0.5) about our future.

**speaker# Liu + speaker# Song**

- **1:** [why don’t you think so]
  **2:** [uh *(..)* ] I don’t have plan to(0.3) get married[hum].(0.4)I think too far away(1.4)uh to think about(0.5)marriage[hum].I think I am(0.4) only a kid for my parents, so(0.3) I don’t think about marriage[hum]. And I think(0.4) in modern society, when(0.4)uh(0.3) many(0.3) young people[hum] as many(0.4)graduate student or doctoral student[hum] they will choose to(0.4) enrich themselves and to(0.6) hum study(0.5)uh further more *=* *=* So(0.6)hum they don’t choose to marry(0.5)[hum] with others. So they will(0.5)hum spend more time to(0.4)hum(0.6)study[hum] to learn hum and(0.3)hum to live alone[yeah]not(0.4) uh think about

**speaker# Liu**

- Yeah yeah They prefer to have some free time, right?

**speaker# Song**

- Yeah Free time *=*

**speaker# Liu**

- *=* Yes I also noticed(0.4)recently more and more young people(0.6) they are(0.5)they don’t have plans for marriage or even they are kind of afraid of(0.4) being being like getting married with some guy. So what do you think about this phenomenon?

**speaker# Song**

- (0.5)hum

**speaker# Liu**

- So what do you think[Ok] about yes marriages.

**speaker# Song**

- hum I think(0.4) hum this afraid maybe from the media[hum]. With the development of(0.3) media, we uh (0.3)obtain(0.6) much information from the media from Weibo[yeah], uh and Wechat[hum], and many social apps(0.3), (0.5)uh so(0.5)hum there are many information good(0.5)or bad[yeah], hum(0.7)such as the(0.4)uh domestic violence. We uh we know about it(0.4) from the internet(0.3). I think it’s very hum bad(0.6) And hum(0.4) it will cause pressure for(0.3)uhmarried person[hum] or they really to(0.5)hum to get married[yeah]. but from the maybe(0.5)uh maybe[hum] my husband will hurt me[yes]. They are

**speaker# Liu**

- They are afraid of getting hurt from[yeah] their partners[0.5]. Yes I think that’s one of the factors that make people(0.4) being afraid of marriage recently. And I also think(0.6) hum maybe, it’s because(0.6) as me as for me I’m the only child in my *:* my family(0.6), so(0.4) maybe we got like this strong personality[hum], so when we(0.6) facing when we are facing some quarrels some conflicts we don’t know how to compromise with each other[yeah]. We’ll just stick to our(0.7) whatever I think. yes and I think(0.4)just I will just break up with you if you don’t agree with me[yeah], so I’m afraid if I keep doing like this, I maybe(0.6)like got divorced easily, so that also makes me(1.5)a little bit afraid of getting married yes.

**speaker# Song**

- Yeah if you have a child, you don’t have many right[yeah] to hum(0.6)to start new life[yeah yeah]. And I also think(0.6)hum the reason for young people they don’t get married maybe uh because there(0.4) many pressure from the society[society], from the life[hum] such as the economy[hum]. uh We are know uh in this year of 2020[hum]uh the(0.5) COVID-19 outbreak[hum] and they cause many bad things uh for(0.6)for the humans[hum] such as the young people can’t find appropriate jobs[yeah].uh So uh if they don’t have job they(0.3)uh(0.4)the(0.4)they are elder uh they don’t have the support from their parents. They need to earn money[yeah]uh by themselves(0.7)uh but they don’t have job[hum] they will have(0.8) they will uh they don’t have the money[hum] They can’t hum live better[yeah] hum(1.4)so it’s very uh it's very(0.9) difficult[hum] to support family so they a little afraid of[yeah] marriage.

**speaker# Liu**

- And just now you’ve mentioned children, kids right?

**speaker# Song**

- Yeah

**speaker# Liu**

- And also I also think marriage is(0.4)is about a commitment it’s about responsibility[hum], especially when you guys got kids got children. Sometimes I will think it’s(0.7) more like some burden for our young people, because I think(0.4) even me I’m now still a child like[yeah] I don’t know how to deal with another child or more. So I think maybe that's caused(0.6)hum some young people don’t want to get married(0.7). So although(0.4) we got those uh bad things, bad effects of of being afraid of marriage(0.4), some people still like they have the hope for a good marriage, right?

**speaker# Song**

- Yeah they hum Such as me[hum] uh I uh knew the society phenomenon is very bad. And there are many bad things[hum] hum about between hum between couples[hum]. and they will very variant and they will uh very be strong husband. uh so I I still believe(0.5)hum(2.3) there exists(0.6)hum harmonious relationship(0.8)uh and(0.8)uh you will find the true love[hum]. And(0.4)uh you can do many things such as travel[hum], hang out together[yeah] and or support child[yes]. It is very hum hopeful[yeah].

**speaker# Liu**

- (0.5)So uh what do you think(0.3)are some like essential factors(0.3)in a(0.3) marriage?

**speaker# Song**

- (0.8)hum I think the first I hum is very important uh factor is the level of education[yeah]. hum In terms of me[hum], I am a graduate student, maybe I want to get a doctor degree[hum]uh so uh I(1.4)I don’t find a husband he is a junior[hum]. I think if we have the same level of education[hum] we will find many(0.6)hum many(0.4)hum ideas[yes]and topics[hum] to talk about to study and learn together[hum]. Yeah so *:* I think It is very important and hum if we had a same level of education maybe we can find a[hum] uh similar(0.7)job[yes] And it’s very convenient[yeah yeah].

**speaker# Liu**

- hum so As for me I think uh important things influence marriage are from family background[yeah].yes Like opinions of the family members or even like if your parents they’ve got a(0.5) very successful marriage, then the kid will probably hold this positive attitude towards marriage, right? and(0.4)I think that and their parents will influence the this person’s like personality >a lot<(0.8). like uh So if you if you find someone with like a good tempered or very(0.9)like(0.6) easygoing person[hum], you can make a like a good relationship.I think so.[yeah]hum

**speaker# Song**

- hum and hum in terms of the family background[hum], maybe the uh many parents[hum] choose to urge young people to uh easily[hum] and earlier to find a husband[hum]. It's very it's very bad. And it is very noise(0.8)

**speaker# Liu**

- Do you parents do so?

**speaker# Song**

- Yeah.

**speaker# Liu**

- yes?(laughter)

**speaker# Song**

- hum and I also think the factors(0.4)uh to choose a appropriate person maybe uh is also in terms of the economy[yes]. Only you have enough money[hum] and you can support your child and family[hum] and to live better.

**speaker# Liu**

- Yes(0.5)and and more and more people like pay attention to money, to material things recently. But I think(0.4)they should do so(laughter). Yes I think money is like a foundation of a better life. You know but Last but not least I think(0.4)I think the most most important thing in a marriage is is the true love between the two people. Do you think so?

**speaker# Song**

- yeah

**speaker# Liu**

- It’s like if you don't if you guys don’t have the feelings for each other(0.5)then all the like additional conditions we talked about we mentioned is just for nothing. Because[yeah] marriage is(0.4)is based on love in the end I think. So(0.7)(crack) I think that’s all I got to say.

**speaker# Song**

- Oh yeah? Although my parents urge me to[hum] to get married uh(0.5)earlier[hum], and there are many bad things from the media from the society from[hum]hum young person’s pressure[hum], they can’t marry they can’t get married. But we still believe hum(0.5)one day we will find a perfect love[yeah] and to maintain the perfect relationship[yes] with my hum future husband. It’s very perfect and it’s hopeful. hum So(clear throat) although the things effect are bad but I still(1.2) believe[hum] and hum struck to believe

**speaker# Liu + speaker# Song**

- **1:** The perfect marriage[right]
  **2:** [yeah]

**speaker# Liu**

- Yes so Thank you that’s all we want to talk about today. Thank you

**speaker# Song**

- Thank you.
