# Supplementary material for: Multimodal Irregular Self-Selection in Chinese Postgraduate English as a Foreign Language Learners’ Conversation: When, How, and Why
Source: Front Psychol. 2022 Mar 25;13:788438. doi: 10.3389/fpsyg.2022.788438 (PMC8990892; doi:10.3389/fpsyg.2022.788438)
Supplement: Supplementary file 3 [file Data_Sheet_1.zip › Transcribed data/Group 17.docx]

***Supplementary Material***

**speaker# Cai**

- so about lifestyle, which lifestyle do you prefer? Faster or slower?

**speaker# Yang**

- (0.5)Maybe as for me, I’d like to live in low peace life, because you know uh I think(0.6) one's uh lifestyle have match his spirit or character, so for me I am a you know peace people, and not so competitive so uh maybe more slower lifestyle may be hum suited for me. And how about you?

**speaker# Cai**

- I agreed with you, I prefer slower lifestyle too, because I think uh now in modern city，people are busy with stuff and they have no time to enjoy their life, so if I have the chance to choose, I would choose slower lifestyle, it will make me uh more comfortable and easy maybe I can say that, so compare with faster lifestyle, I prefer slower lifestyle.

**speaker# Yang**

- Ok hum so are you satisfied with your life now?

**speaker# Cai**

- Yes, because I choose what I live(1.0) what about you?

**speaker# Yang**

- hum Yeah，I would say yes, because you know we don’t have so much courses now, and we can have enough time for ourselves like[/yes] we can read those uh not those major books, we can read what we like and we can do some exercises, and we can have[/hum hum] enough time to hum go out like traveling or enjoy some yummy dishes and that's I think is good for me.

**speaker# Cai**

- So, what kind of food do you like?

**speaker# Yang**

- Food? You know I am a southerner, so I don’t like those salty or sweet food like[/hum] those northerner like, and I like more spicy or some fresh food.

**speaker# Cai**

- (1.1)Fresh food? Like what?

**speaker# Yang**

- No, I mean the hum the process of making the food is like raw material[/oh] not like pickle or some kind You know.

**speaker# Cai**

- I know

**speaker# Yang**

- And How about you?(laughter)

**speaker# Cai**

- I think, hum just like you said, now we have no we don’t have much lessons, so we have[/yeah] a lot of spare time[yeah]we can do whatever we want, although hum the school forbid us to go out(0.8), but we still have a lot of(1.3)hum opportunity[yeah]right?[yeah](1.3)After all, this is a huge school.

**speaker# Yang**

- So we can just going around in our school.

**speaker# Cai**

- Yes

**speaker# Yang**

- Yeah hum what is your ideal life for you?

**speaker# Cai**

- My ideal life first of all, I think we I must have a job.

**speaker# Yang**

- Yeah [that’s the basic]

**speaker# Cai**

- [I must]I I must earn a lot of money, so that I can do whatever I want, that is very basic I think, and futhermore I think my ideal life that hum despite work, I could have time to travel whatever everywhere maybe hum[yeah] so uh my ideal life that I want(0.9) to have my own house, that is very important[yeah]and

**speaker# Yang**

- and a car?

**speaker# Cai**

- car? No It is not necessary, but house is very[/yeah] necessary, and I want my parents have a good body, and my work will have a(1.7) I will have a good job, and I have time to travel, That is my ideal [life].

**speaker# Yang**

- [hum]Speaking about your parents, would you live with your parents in your future?

**speaker# Cai**

- Maybe not live together at all I think I will live near with them.(0.7)[Ok]maybe the same

**speaker# Yang**

- Same city?

**speaker# Cai**

- Yes[hum]Or more nearer in a same[/ok] maybe a same neighbor[area]yeah

**speaker# Yang**

- region yes[/yes]got you.

**speaker# Cai**

- (0.8)so[/hum] what about your what about your ideal life?

**speaker# Yang**

- (0.8)hum for me I think(1.0)we have almost the same(0.9)uh thought, you know[yes]Ok first job is basic and[/yes]and house that’s right, maybe I want a car, because although I didn’t have the driving license, I will get it, and I want I enjoying driving in a cay you know

**speaker# Cai**

- But don’t you think

**speaker# Yang**

- Dangerous?

**speaker# Cai**

- No

**speaker# Yang**

- Traffic gam?

**speaker# Cai**

- No Have car maybe raising a car is [a little bit[/expensive]EXPR?

**speaker# Yang**

- (0.7)hum yeah that’s a deal but hum

**speaker# Cai**

- So you are still choose to have a car

**speaker# Yang**

- (0.5)yeah，because[/ok] hum sometimes you go with your friends or parents[hum], you can just hum take a bus or take a taxi[/yeah], and if you have a car, you can go more free, you know just just going out and drive the car wherever you want[hum], hum and I also enjoy driving a car alone, because hum you know that’s the time for myself, and no else besides me and I just have focusing on driving[hum] you know and uh let all things alone yeah.

**speaker# Cai**

- Talking about the car, I have some thought that this summer I went to a big city with my friends, but we

**speaker# Yang**

- Big city where(laughter)Which big city

**speaker# Cai**

- Big city just bigger[/big city] than my city yes[ok] that uh we went to there to play a game, and we don’t have a car so when we want to go back home, we choose[uh]

**speaker# Yang**

- [Walking]?

**speaker# Cai**

- We choose to didi[ok] but we waited a very long time, very very very long, so [in that]

**speaker# Yang**

- [Is in] midnight?

**speaker# Cai**

- Yeah yeah so at that time, I think maybe I would have a car(laughter)is better[hum], sometimes I think car is necessary but

**speaker# Yang**

- But if you don’t have a car, which transportation would you like to go? Just[/hum] walking or Bus, bicycle or the subway?

**speaker# Cai**

- Walk[walk]just walk，[because my]

**speaker# Yang**

- [Ok that's]healthy(laughter)

**speaker# Cai**

- Hometown is very small[ok] so talk about the lifestyle, how about your friends, what are they live, what are they lifestyle are?

**speaker# Yang**

- hum you know it's normal for the young people now to[/hum](0.8)just(0.8)doing whatever they like[yes]but but uh let alone their body you know they just stady in a late night and hum eat a lots of junk food maybe those friends around me they like this because and but hum mabe they are less care about their health, but hum I think it’s okay, because[/hum] that's their choice, hum if they can(0.8)uh realized about the healthy issue maybe they will change about their lifes[hum]lifestyle and live in a more health way[hum]

**speaker# Cai**

- Anything else?(0.8)[hum] What kind of the way they study(0.8)[or]

**speaker# Yang**

- [Studying?]

**speaker# Cai**

- Yeah

**speaker# Yang**

- hum Some of my friends are working now and I didn’t have a lot of friends in my university, in my postgraduate, so hum one of my best friends, she is working in in my university, and two of my roommates they hum keep their studying, one in Guangdong and another one is still in xi’an , and the rest I didn’t have their(laughter) I didn’tkeep in touch[ok](0.7)how about your how about your friends.

**speaker# Cai**

- (0.8)My friends hum(3.0)my friends(2.9) just like your friends(laughter), most of them are studying and just one(0.6)is working, and the studying and the studying friends are just studying(laughter)

**speaker# Yang**

- hum does she does he has a goal to achieve like for like you to go to another university? for his postgraduate study

**speaker# Cai**

- Yes

**speaker# Yang**

- Ok so the first time he failed?

**speaker# Cai**

- No no no he(1.2)

**speaker# Yang**

- He just change his mind like first of all I want to find a job but then I realize[/Ah]

**speaker# Cai**

- No both of us first opinion is must keep study[hum]and(0.9)because some of things some of them didn't go to university but(1.0)

**speaker# Yang**

- Oh my god I got it(laughter) So you mean the friends in high school not in the university?

**speaker# Cai**

- No they just[/I]they just working

**speaker# Yang**

- Ok [hum]working and still studying?

**speaker# Cai**

- Yes

**speaker# Yang**

- Ok Got you finally I got you(laughter)[hum]

**speaker# Cai**

- No higher school

**speaker# Yang**

- Ok and(0.9)what will you do to achieve your hum ideal life[hum]? like To get what you want.

**speaker# Cai**

- (0.9)Because we are students now right[/yes] we must study very hard, so so that we can find a good job[hum](laughter)[yeah] a lot of things basic on the jobs right?

**speaker# Yang**

- Yeah

**speaker# Cai**

- And maybe except for study , I want learn some new things.

**speaker# Yang**

- New skills

**speaker# Cai**

- Yes New skills in university, because our our school is very good school right[hum] so we can learn a lot of thing.

**speaker# Yang**

- Yeah Beside[/and] our major

**speaker# Cai**

- Yes[ok]and I think maybe this is the only chance that we can keep studying right?

**speaker# Yang**

- So you like studying?

**speaker# Cai**

- No maybe I don’t like studying, but I

**speaker# Yang**

- You have to

**speaker# Cai**

- Yes we have to, not[hum]not just me and is I think it just like this keep studying and keep improving myself.

**speaker# Yang**

- Keep looking

**speaker# Cai**

- Yes keep calm yes[hum]so what will you do?

**speaker# Yang**

- For me(0.8)I think we are in the same you know we are in the same situation we are in the same satus.

**speaker# Cai**

- Maybe we choose to uh we choose to get master degree in here, that that is what we to.

**speaker# Yang**

- Yean[yes]I got you

**speaker# Cai**

- You get me

**speaker# Yang**

- hum[ok]so(1.0)

**speaker# Cai**

- So no more questions?

**speaker# Yang**

- (1.7)hum Do you have anything to ask about?

**speaker# Cai**

- No Ok nice to talk about talk with you

**speaker# Yang**

- Yep It's exactly for me.
