# Supplementary material for: Multimodal Irregular Self-Selection in Chinese Postgraduate English as a Foreign Language Learners’ Conversation: When, How, and Why
Source: Front Psychol. 2022 Mar 25;13:788438. doi: 10.3389/fpsyg.2022.788438 (PMC8990892; doi:10.3389/fpsyg.2022.788438)
Supplement: Supplementary file 3 [file Data_Sheet_1.zip › Transcribed data/Group 18.docx]

***Supplementary Material***

**speaker# Zhao**

- So let’s start today’s uh discussion[hum]. Today our discussion topic is environment issue. As we know, the environment is currently changing and people need to raise our awareness uh to face the environmental uh problems surrounding us. and With some influence of natural disasters, warming or cooling periods and some extreme weathers, we need to know what happened in our uh environmental on the earth. So what kind of environmental problems you know?

**speaker# Yu**

- uh I know some environmental problems like uh climate change and the rise of sea level uh because[/ok] of the warm weather. The icebergs in some areas may uh melt they may melt[hum] so the level of the sea, uh so the sea level will rise, which[/yes] cause a big problem to our humanity.

**speaker# Zhao**

- (0.7)Yes and as I know uh global warming uh appears in our uh environment. uh The temperature has been uh has been elevated on the surface and on the ocean. So the(0.4)polar ice cap start to melt and it triggers many uh natural disasters, like flood, excessive snow drought or the uh desertification. So uh do you think how we can deal with the global warming, such kind of environmental issues.

**speaker# Yu**

- (0.4)Glo- Global warming is[/hum] an important issue. What[/yeah] we can do I think[hum] is just to reduce the emission of the carbon dioxide[yes], like the green house gases[hum] and use some innovative ways to uh power our electricity like[/yes] wind power, or[/yeah]solar power instead of some fossil uh fossil oil, like this.

**speaker# Zhao**

- Yeah uh You mentioned uh there are some energies or resources are used in our daily life. so uh I suddenly think about one environment issue is the resource depletion. hum In the world, actually uh due to some scientific researches uh human beings just only need more than one earth to satisfy our resources use[Yeah] uh yeah and the so we can find some resources to replace our use, like uh solar resource, wind, radio active or uh hum the water solar resource to help us save the energy.

**speaker# Yu**

- Yeah Of course. And you mentioned water. I think[/yeah] water is an important source of our daily life[hum], but in some uh sea areas, we can see a lot of plastic(0.3) materials in the sea[yes],hum maybe abandoned by our human[hum] and eaten by the sea uh animals[yes], but actually I think these plastic materials will finally return back to our human because we have to eat the fish, uh but the fish has eaten the uh plastic materials[yes] and finally go back to us. I think this is a big problem we have to solve.

**speaker# Zhao**

- Yeah yeah you mentioned some uh ocean uh animals are uh badly effected by the uh plastic or white uh waste[yeah]. So these plastic or white materials truly affects the diversity of the animals. uh Some animals, like uh precious fishes or the polar bears life are terribly affected. You cannot uh have the uh stable prospect in their future lives.

**speaker# Yu**

- Yeah[hum] maybe if they eat the plastic bags in their stomach[yeah], they can’t breathe so they will die. I think this is not good for our diverse bio-diversity.

**speaker# Zhao**

- (0.4)Yes[yeah]So uh if we want to maintain the diversity of the animals, the UN or our public or our people should cooperate with each other and uh follow the uh polices on the environmental protection.

**speaker# Yu**

- Yeah Of course. And I think we have a problem overpopulation[/hum].

**speaker# Zhao**

- Yes

**speaker# Yu**

- Yeah China has a large amount of population, so[/hum] we have to deal this hum environmental pollution or water pollution, noise[/hum] pollution[hum] in our joint efforts.

**speaker# Zhao**

- Yes

**speaker# Yu**

- What do you think about the noise pollution?

**speaker# Zhao**

- (0.5)uh The noise pollution uh is the artificial pollution. It's not the natural uh variety of uh pollution. And the noise can come from the factory or the uh cars or the streets. And I think one important or prominent pollution is the hum water pollution. As you see, it really threatens the lives of animals and human beings.

**speaker# Yu**

- Yeah Of course. We live in[/hum] one earth and environment is our base. [So we have[/yes] to uh find a balanced way to boost our economy and to[/hum] protect our environment.

**speaker# Zhao**

- (0.7)Yes And the uh I think uh soil degradation is also the environmental issue. hum like we hum through out some wastes some[/hum] plastics and some hum uh anti-ecosystem materials into our soil and that will made the soil become tough and hard. it really has negative effect on the crops or fertility rates of our plants.

**speaker# Yu**

- hum And[/hum] I heard some plastic bags may[/hum] not degrade in many years after [being buried[/yes] in the soil[yeah], it’s too terrible for the environment, because if only if we have done these, many crops will not be available for our human, so we won’t get enough food in our daily life(0.7)[yeah] So it’s not just like a circle. If we don’t[/hum] protect the environment, the bad effects will finally come to us. [So we have[/yes] the responsibility and obligation to protect our environment as we can do.

**speaker# Zhao**

- Yes I can't agree with you more. and Do you some other environmental issues that hum is un relatively uh new or a novel for us? Like the new technology(0.3) uh pollution.

**speaker# Yu**

- (0.8)uh Maybe sometimes the[/hum] fossil oil we use could create more carbon dioxide[yes] such as the waste of cars[hum]. uh We have to use some uh electronic cars to uh replace [the old[/yes]way uh in the future. Maybe this is a good way we can solve.

**speaker# Zhao**

- Yes. The carbon dioxide the greenhouse gas emissions can be uh under control in this way. and Some heavy mental or some hum wastes produced by the uh fuel-driven cars can be also uh decreases.

**speaker# Yu**

- I think it’s a great way. and We should explore this [in our[/hum] future to better serve our earth.

**speaker# Zhao**

- Yes So this is our uh today’s topic. And I'm very delighted to have the uh nice discussion with you on the environmental issues. Hope to discuss with you more topics in the future.

**speaker# Yu**

- I'm uh I'm glad to have this uh grateful discussion uh with you today. And [I hope[/hum] we will do whatever we can to protect our environment after this. Thank you.
