# Supplementary material for: Multimodal Irregular Self-Selection in Chinese Postgraduate English as a Foreign Language Learners’ Conversation: When, How, and Why
Source: Front Psychol. 2022 Mar 25;13:788438. doi: 10.3389/fpsyg.2022.788438 (PMC8990892; doi:10.3389/fpsyg.2022.788438)
Supplement: Supplementary file 3 [file Data_Sheet_1.zip › Transcribed data/Group 19.docx]

***Supplementary Material***

**speaker# Han**

- hum Hi what a fine day(laughter)hum(0.6)It’s very suitable for traveling and We can enjoy the sunny day andHow about you? Do you like traveling?

**speaker# Wang**

- Yes of course. I like I like traveling. uh Because firstly travelling can relax myself from the pressure of study and work. Every time when I finished my journey, I will I was full of happiness and energy. uh In addition travelling is educational. uh What I say is the people you met in the journey are are usually very interesting. uh I also uh I can broaden my horizon because I can see many different customs in different places.

**speaker# Han**

- (0.3)Yes I agree with you. and I love travel too. So Where did you go for traveling?

**speaker# Wang**

- hum My longest uh trip was to Guilin. I stayed there over one week. uh It was during the national day uh Guilin is a very beautiful city. You can see rivers and mountains in the center of the city. Besides the life the life moves slowly there so you can find many foreigners come there to enjoy the life. uh also it is common to see some people sitting sitting beside the rivers, chatting with each other. It is a very comfortable lifestyle.

**speaker# Han**

- (0.3)Oh that’s great. I have not been to Guilin. and hum Do do you have a deep impression hum I mean do you enjoy yourself in Guilin?

**speaker# Wang**

- (0.3)Yes but also no because it is hum it is not a very good thing. I planned to visit the Elephant Trunk Hill hum but failed(0.4) due to the flooding and rains. uh The Elephant Trunk Hill is a symbol of Guilin so I really want to visit it. hum So it is so regrettable! Right? uh Next time uh if I have chance, I will go to Guilin again and have a visit.

**speaker# Han**

- (0.4)Oh What a pity.

**speaker# Wang**

- Yes uh Have you been some have you been some places in China and Do you have some interested things to share with me?

**speaker# Han**

- Yes, of course.hum Actually I uh have been to Changsha and Zhangjiajie last summer. It’s really a wonderful experience. hum I tasted many the famous Hunan cuisine and I tried the uh cable car as well as the glass skywalk，it was a great challenge for me and I consider that I became more brave after this journey.

**speaker# Wang**

- Oh what a small world! I have been I have also been Changsha uh maybe several years ago. but hum I I remember there are a lot of nice food to eat. But mostly very spicy so I can only try a little. But it is very delicious even it is very spicy.

**speaker# Han**

- Yes yes Changsha is a hotspot area that enjoys a good reputation for its delicious food such as Wenheyou, stinky tofu and chayanyuese. uh I love chayanyuese so much that I hope one day it can open a store in our city. uh And I l also went to the uh Orange Island that was introduced to the whole world by Chairman Mao’s famous poet QIN YUAN CHUN[hum], yes it impressed me a lot.

**speaker# Wang**

- Oh I I know chayanyuese. It is very popular in recent[yeah] years. But I haven’t tried it. I also uh hope to try it later someday! uh do you have Have you visted some places in Zhangjiajie?

**speaker# Han**

- Hum Yes hum I have been to the Wulingyuan Scenic Spots and Tianmen mountain. hum The traditional Chinese uh poet Li Bai[hum]once wtite a poem Viewing the Tianmen Mountain. I consider that you must have learned it. And uh as for the Wulingyuan it is a must-see place if you travel to Zhangjiajie. And the most fantastic scenery in Wulingyuan must be the hum quartz sand rock peaks uh covered with uh green forests. uh I have seen a lot of different shapes of the peaks such as hum RMB, hum old man[hum], or some specific animals, and(0.4)uh it really impressed me a lot!

**speaker# Wang**

- Sounds fantastic! You really enjoy yourself there. I know Zhangjiajie. It is a city uh enjoying a good reputation[yeah] for its scenery. Next time, if I have if I go there, I might ask you about your travel arrangements and your suggestion(0.4).uh Speaking of the travel uh traveling, going out by yourself or with friends or families,uh which do you prefer?

**speaker# Han**

- (0.3)hum As for me uh I prefer to travel with my friends or families. hum Because, you know,hum we can take care of each other and when uh we go to some new places[hum], uh we often(0.3)like to take some photos(0.3). At this moment the importance of going with others is obvious and uh I also like to share delicious food with other people!

**speaker# Wang**

- Oh I I agree with you. But I prefer to travel by myself. uh It is very free to go anywhere I want. hum If there are too many people, I will be limited for uh be limited for my for some food, plans. uh Even worse, I might need to give up something.

**speaker# Han**

- (0.5)hum Yeah I realize hum traveling alone is a freer[yes] than going with others[hum] and you must be more relaxed[hum]. Different people have different traveling habits[hum], I mean so hum it's not very strange.

**speaker# Wang**

- (unclear)

**speaker# Han**

- hum and uh We all know that the National Day[yes]you know and together with Mid-Autumn Day is coming[hum], but the(0.6) Covid-19 pandemic[hum] isn’t over yet. So we had better stay there. hum And if when all is over[hum] which place do you want to go?

**speaker# Wang**

- (0.7)I want to go Japan. I like this country for its language, food, scenery. Besides I really want to try the Japan Kimono. uh I often see Japan TV series and can find Japanese girls wearing the Japan Kimono in a very comfortable and natural way. We have we have Chinese Han costume[yeah] so I really want to see different clothing in different countries.

**speaker# Han**

- Oh What a coincidence We have the same destination. I’m attracted a lot by the beautiful scenery in Japan, such as sakura and Fujiyama[hum]. I really hope that one day I can stroll along the road covered with sakura[hum]. And as I mentioned before, I’m quite interested in detective, and Japan is the hometown of the famous animation[yes], detective Conan and I really hope that I can have an opportunity to travel there.

**speaker# Wang**

- (0.5)Oh oh Sounds amazing I guess we can go there together right(0.5)? uh I am so happy to find a find one friend with the same goal with me.

**speaker# Han + speaker# Wang**

- **1:** (0.6)Yes I agree with you we can go together to Japan. And hum(0.8)while while traveling are you used to making some uh travel guides(0.3) [before] leaving?
  **2:** [yes]

**speaker# Wang**

- Yes. I usually make travelling plans before my trip. Because I think the plan uh can save me a lot of time so that I can enjoy every place in the destination I want. uh Besides I will find the convenient way to travel among the city.

**speaker# Han**

- (0.5)Oh You are so careful. I’m looking forward to hum traveling with you(0.6) an *:* d uh since we talk about traveling uh Which way do you prefer while traveling?

**speaker# Wang**

- (0.3)I guess uh In China I like to travel by train. uh Here are some reasons. uh Firstly I can when I travel by train, I can see more of China(0.4).uh Secondly, uh actually it is comfortable and convenient if we travel by train because people can go anywhere in China and people can have a rest in train. and Lastly, traveling by a train is a kind of social event[hum]. You can chat with others uh asking why they are going out or where they are going.

**speaker# Han**

- (0.4)Yes hum travelling by train is a really a funny and comfortable way[yes], but I prefer to travel by air[yes] because it is very uh fast and I can save a lot of time.

**speaker# Wang**

- (0.3)Yeah I I agree with you. hum Then do you have some uh funny things when you travel by plane?

**speaker# Han**

- hum Actually I didn’t have much fun on the plane I usually have a rest with my hum earplugs[hum] because of the noise on the plane. But hum once I noticed a strange person who constantly asked the the hum stewardess to get drinks[hum] to him[hum], and the stewardess hum always politely met his requirements[hum]. I thought that the service industry is painful, we must respect each other[yes]. hum How about you?

**speaker# Wang**

- (0.7)hum I think the most interested thing in train is that people can play cards with each others. You know there are a lot of people in the train, so people need to find something to kill the time. Otherwise it will be a torture. So playing cards is a convenient way to *:* to kill time. People will choose it. It's very funny.

**speaker# Han**

- (0.3)Oh that sounds so interesting I hope uh next time I can have a try. But if we want to go to Japan, we must get there[yes] by air[yes]. and If we are lucky enough, we can uh buy the discounted ticket[hum]. Because we are still raised by our parents and uh we had better save some money.

**speaker# Wang**

- You are so clever(0.5). But uh I don’t know uh how to buy these discounted tickets. Where do you Where do you buy them?

**speaker# Han**

- I’m sorry hum actually I usually get it by luck(laughter), but I consider that[hum] we can deal it in a long-term perspective.

**speaker# Wang**

- (0.3)Or *:* we can compare some tour groups to find a cheaper one. I know that some tour groups have their own planes uh travel arrangements or buses in the destination.

**speaker# Han**

- Oh uh I know it. My parents hum always travel in a group[right] and it's[hum] really convenient[hum]. But hum they usually bring some expensive but useless presents for me. it bothers me a lot.

**speaker# Wang**

- I knew it. uh Last time last time when I go when I went to Huangshan on the way to the destination, the guide introduced a lot of uh special local products, such as Huangshan tea and special food. uh But I refused it bacause uh due to its price. uh Then I searched them uh in in Taobao application and buy them with cheaper price. It saved my money.

**speaker# Han**

- Oh you reminded me(laughter).hum Actually the products sell in Taobao hum often comes from the uh source area[yes], maybe next time I can persuade my parents in such words. Thank you a lot.

**speaker# Wang**

- (0.6)Yeah then please remember to ask you parents about the contact information of the tour groups. Because we may need need to contact them to find to find a cheap price if we if we need to go to Japan.

**speaker# Han**

- Yeah yes no problem.

**speaker# Wang**

- Ok then I am looking forward to go Japan with you.

**speaker# Han**

- Me too. That's all.
