# Supplementary material for: Multimodal Irregular Self-Selection in Chinese Postgraduate English as a Foreign Language Learners’ Conversation: When, How, and Why
Source: Front Psychol. 2022 Mar 25;13:788438. doi: 10.3389/fpsyg.2022.788438 (PMC8990892; doi:10.3389/fpsyg.2022.788438)
Supplement: Supplementary file 3 [file Data_Sheet_1.zip › Transcribed data/Group 2.docx]

***Supplementary Material***

**speaker# Yang**

- So what kind of movie do you like(0.3) about[/hum] some words or something else?

**speaker# Jiang**

- Okay I uh want to mention two movies, and[hum]first movie I want to talk about is “Duo Guan”. hum[/yes]This is a very famous movie I saw the day before yesterday. hum[hum]And It was also shown the day before yesterday. It tells a story of Chinese women’s volleyball game[/yes]. hum and The story is very touching, because from 1981 to 2019, hum[/yes]the Chinese volleyball game has won hum 10 championships in international games, hum including the 1981 uh the International Volleyball Champion in Japan, and the 1984 the Los Angeles Olympic games, and the 2016 hum Rio Olympic games. And [we can also[/hum] see some uh spirit, I call uh volleyball spirit behind this movie. And I think this spirit is hum remaining true to our first aspiration and[/yes] never giving up. hum and

**speaker# Yang**

- I think so I think the competition must be very fierce(0.5). Yes. And does(0.9) they will get hurt. Get it injury you know

**speaker# Jiang**

- uh Yes [and]

**speaker# Yang**

- [Due to]the yes

**speaker# Jiang**

- Yes the the girls have to face and overcome many setbacks and challenges[/yeah]in the process of their uh daily hum practicing, they will be as as you have mentioned, they will get hurt. And they all will also be they will also be exhausted after each training. But they overcome and conquer it[yes] So they achieved the championship.

**speaker# Yang**

- (0.6)Yes And I think this kind of spirit will inspire us for a long time[yes]. And it is also the patriotism.

**speaker# Jiang**

- [It deserves[/yes] our learning, especially to us students, whenever[/yes]hum whatever our majors [may be[/yeah]

**speaker# Yang**

- (0.6)I think so

**speaker# Jiang**

- hum So what kind of movie hum do you want to mention?

**speaker# Yang**

- (0.8)And The movie I saw some days before it called the “Eight Hundred”. you know It is a movie about the senior Japanese war. and[/hum] The main plot is about uh the fully injured, healed out against the Japanese waves ,for this at nights, and they defense the “ Si Hang” warehouse in Shanghai in 1937[hum]. And I was touched by the Chinese soldiers, their patriotism, their courage(0.5). They will never give up and they protect us. And they protect their own family. They protect the Shanghai and the and the whole country you know[and I touched]

**speaker# Jiang + speaker# Yang**

- **1:** [Yes and when I think]
  **2:** by their[competition]

**speaker# Jiang**

- [And I think] hum apart from patriotism, hum persistence and bravery is also a very uh a kind of spirit that the movie wanted to show to us students. hum I think hum and to us to us student hum Bravery and patriotism is also very important things[yes]especially in our study.

**speaker# Yang**

- Yes And(2.0) they are so courageous. And they also protect people regardless of their sacrifice and their laps. And I was impressed. And the tears broaden out from my eyes.

**speaker# Jiang**

- Sure. a patriotic hum Chinese man always will to have their tears off after seeing such kind of things.

**speaker# Yang**

- Yes I think this kind of movie should be played more times in our country.

**speaker# Jiang**

- You mean it can inspire to uh love our country?

**speaker# Yang**

- Yes and we shouldn't forget the(0.7)you know the shame yes and the price that we paid in this Sino-Japanese war[yes]. and yeah You just mentioned two movies that you watched. I think What's another one?

**speaker# Jiang**

- You mean another movie？

**speaker# Yang**

- Yes，another movie.

**speaker# Jiang**

- hum another movie I want to mention, maybe hum “Shao Nian De Ni”yeah]. And the main character the main actor and actress in this movie, I think is Zhou Dongyu and Yiyang Qianxi. And the most touching character in this movie, maybe uh Chen Nian acted by[/yeah] Zhou Dongyu. And in this movie, Chen Nian is a very uh good student who is good at all of the subjects in her school. But she is also a unsociable girl, hum so that she was bullied by some of her classmates. But hum I can still remember a few days before the college entrance examination, hum she killed one of her classmates who bullied her hum by accident by accident. And finally, even though she was admitted by her hum beloved university, hum she cannot go there to study[yeah] and register at that school because she was a murderer. So I think this movie tells us hum we should behave according to law, even if we are mistreated. And we can hum fight for our rights uh by legal means. That's my personal understanding of this movie. I think it a very educative movie.

**speaker# Yang**

- Yes, I think so. And the school bullying is become more and more common in the school. you know

**speaker# Jiang**

- Yes

**speaker# Yang**

- Yes and they it often happen in the teenagers[yes]. So I think the student uh the teachers and parents should teach more knowledge about this to prevent this happen.

**speaker# Jiang**

- And I also think hum our country should carry out some relevant laws to about this kind of hum events so that our teenagers and even college student can be protected hum well(0.3) [That's my personal] understanding

**speaker# Yang**

- [yes I think so] and I think the students should stand up to stop this phenomenon. And they should have a sense of injustice(0.7) uh a sense of justice[yes] you know they shouldn't be indifferent(0.7). [hum yes]and so are many people will(0.8)some will be suffering in this.

**speaker# Jiang**

- hum Yes We we should not abuse others. hum And we also need to know how to avoid being abused by others. That’s my hum personal that's my view.

**speaker# Yang**

- So what do you think the performance Yiyang Qianxi in this movie? because I liked him very much.

**speaker# Jiang**

- Yes, he's also a very important character in this movie. hum In this movie, he was called xiao bei. And[/yes] he is uh he is just a boy who dropped out of school in middle school. And at the beginning, hum he think he is not the kind of person with Chen Nian, the character acted[/yes] by the by Zhou Dong Yu[/Zhou Dong Yu]. But finally, I think he became more confident and he become the friend of Chen Nian[yes]. hum And I think hum it's also a very common phenomenon that hum everyone can be friends of each other regardless their hum education background, their races and their hum genders.

**speaker# Yang**

- (0.6)I think yes I think so and I think in the movie, he is so handsome[yes]. And he become an adult from the teenagers[hum]. after he has been through this kind of things.

**speaker# Jiang**

- Yes uhm so(0.5)what kind of movie uh what movie do you want to uh share with me, uh apart from the from “Ba Bai”.

**speaker# Yang**

- Yeah yes It is the bucket list. And it is an American movie. Something about the comedy and the main plots follows two ill men[hum] and they shared the one world. and then They know each other. so They want to do some things that they didn't do before they lives. So they want to do something before they died

**speaker# Jiang**

- (1.2)hum So what kind of hum I mean positive energy hum did the movie want to share with us?

**speaker# Yang**

- (1.5)Yes, I think(0.7) life is too short to waste. We should seize the limited time to do something that we want. uh Don't leave them just as a wish. We can take actions.

**speaker# Jiang**

- hum[yeah]I mean in my view, hum after your introduction of this movie, I think the mean hum morality this movie want to show to us is to cherish our[/yes] present life[yes] because[/I think] we cannot foresee what will going hum in the future, right?

**speaker# Yang**

- Yes, I think the life is full of pities[hum]. and the use is very short you know[yes] And now we are 25 years old. And you are(0.7)you are one year older than me. So I think life is too short to waste[yeah]. And I was also impressed by their spirit to fight against the illness[hum]yes because one man is diagnosed the lung cancer.

**speaker# Jiang**

- Lung cancer?

**speaker# Yang**

- So they have to bear the bear the hurt, and injury in the disease. and They have a long road trip to visit some uh tourism attractions. And they go to the(0.8)Xi Ma La Ya mountain.

**speaker# Jiang**

- Mountain Qomolangma

**speaker# Yang**

- Yes yes[hum]

**speaker# Jiang**

- So he's very optimistic one. [hum and a[/Yes I think so] brave one. So hum so we can see some bravery and optimism from this movie. This is a very important hum thing to us and we can learn from it that deserves our learning.

**speaker# Yang**

- Yes I think so. And the movie also conveyed us(0.9)something about the family(0.7)that[/hum] we should keep a good relationship with our family members and don't leave the pity.

**speaker# Jiang**

- hum Ok so what kind of hum TV series do you want to share with me?

**speaker# Yang**

- Oh Yes(0.9). With regard to TV series, I want to introduce you the breaking bad. It is “Jue Ming Du Shi”[hum]. And it is very popular in America.

**speaker# Jiang**

- So what[/and] is the main plot?

**speaker# Yang**

- (0.6)Yes The main plot is about a high school teacher. And his name is Mr. White[hum]. And he was diagnosed the lung cancer[hum]. And he want to

**speaker# Jiang**

- Also the lung cancer?

**speaker# Yang**

- Yes yes the illness. He want to leave his family the financial support before he dies. So he(0.5)[pro-]

**speaker# Jiang**

- [You mean] leave her family a big fortune?

**speaker# Yang**

- Yes so he products and sells the drugs with his former students, Jesse.

**speaker# Jiang**

- (0.5)He produces uh the thing this kind of thing to

**speaker# Yang**

- The drug yeah

**speaker# Jiang**

- To earn more money for her family？

**speaker# Yang**

- Yes, and his wife is pregnant(0.7).

**speaker# Jiang**

- His his wife is pregnant

**speaker# Yang**

- And and She also becomes more suspicious of his unfamiliar behaviors. So their relationship are going is going far away from each other. Yes. And the plot is very exciting and stimulating.

**speaker# Jiang**

- So according to my personal understanding, hum this movie wants to tell us do not be selfish and do not hum break the law hum regardless what kind of hum situation we are facing, right?

**speaker# Yang**

- (0.5)Yes, I think.

**speaker# Jiang**

- (0.9)hum so uh Do you have other series hum TV series you want to share to me?

**speaker# Yang**

- Yes(0.8). But I want you to introduce me a TV series that you have watched.

**speaker# Jiang**

- hum Let me recall. uh(1.7) uh I the first hum TV serie How about San Shi Er Yi? have you ever seen this TV series?

**speaker# Yang**

- (0.9)Oh I have seen the introduction of it.

**speaker# Jiang**

- hum[/yes]yes the hum the TV series tells us the story of three women who lives in Shanghai, this metropolis. hum And they are fighting for their own families and careers. But the three women have different choices hum in the face of challenges in their life. hum and The most touching character of this movie，I think is Gu Jia, She is a super[/yes] she is a super woman hum and she helped her husband a lot in his career. and finally uh his husband was promoted to a engineer uh

**speaker# Yang**

- Yeah Xu Huanshan

**speaker# Jiang**

- Yes hum in Shanghai. But at that time, hum her family also faces some crisis. But her choice is to hum face it bravery. I think it's very hum it enlighten me a lot.

**speaker# Yang**

- Yes and I know they have a love affire

**speaker# Jiang**

- hum yes

**speaker# Yang**

- Yes Lin Youyou You know

**speaker# Jiang**

- Yes And this movie also tells us hum what is wrong and what is right, what [should be[/yes] hum what should be cherished and what should be abandoned. That's my hum understanding of this movie hum[I]

**speaker# Yang**

- Yes [I think] when we live in Shanghai, there are too much pressure

**speaker# Jiang**

- And they are under too under too much pressure

**speaker# Yang**

- Yes because the house price is too high.

**speaker# Jiang**

- Yes hum they have to pay off the housing mortgage every month. and They have to uh save more to do this. That' my under [that's my personal[/yes] understanding.

**speaker# Yang**

- (1.2)So I want to ask you a question[hum] uh when graduate from the NENU[hum], which city will you choose to live ,a big city or your hometown?

**speaker# Jiang**

- hum I think I will live in a

**speaker# Yang**

- Where

**speaker# Jiang**

- Live in a provincial city

**speaker# Yang**

- provincial city

**speaker# Jiang**

- hum not uh not Beijing, Shanghai, Chongqing or Tianjin. I think a provincial city is hum is the most acceptable hum to me because the housing price can still be accepted. And hum we can also have many we can also have many good opportunities in a provincial city. hum[yes] That's my that's my understanding.

**speaker# Yang**

- Yes I want to live in a big city[hum], because you know my hometown is inner Mongolia[hum] autonomous region[hum]. It is so far away from the prosperous places. [So]

**speaker# Jiang**

- [Yeah]you mean Hu He Haote

**speaker# Yang**

- No Baotou

**speaker# Jiang**

- No Bao hum

**speaker# Yang**

- Yes so I choose the big city because there will be more opportunities to get[Yes]. yes maybe I can(1.7)uh I know there are many challenges. hum I think the challenges means more(2.3)

**speaker# Jiang**

- Means more chances

**speaker# Yang**

- Yes yes chances. yes Maybe I can find a boyfriend in a big city[yes]. And he will(0.8)afford the pressure with me.

**speaker# Jiang**

- hum yes

**speaker# Yang**

- yeah

**speaker# Jiang**

- So uh do you have another TV series hum you want to share with me?

**speaker# Yang**

- Yes another TV series is the person of interest and the Chinese name is “Yi Fan Zhui Zong”[hum]. And the main plot is about the government designed a machine to spy on everybody every day. and you know In this case, that no one has the privacy(1.1)privacy[uh]. so And(0.9)there are many crimes involved in the ordinary people[hum]. But the government ignored them. So the two men want to rescue these people in danger and fight against the crime. And one man is a hacker and another man is the former CIA agent. And they work together to help people.

**speaker# Jiang**

- So I think the TV series wants to tell us do not do not commit a crime.

**speaker# Yang**

- (0.8)Yes.

**speaker# Jiang**

- Yes So what kind hum even if we are skilled criminals, we can be caught in the final.

**speaker# Yang**

- (0.8)Yes, I think so and I think it is ironic that[/hum] people design this kind of machine to spy on us[hum].and It is also a secret system. I think we, ordinary people, are cheated by the government. And it also reviews the the darkness of American society. you know

**speaker# Jiang**

- You mean the American

**speaker# Yang**

- polities yes

**speaker# Jiang**

- polities[yes]authority

**speaker# Yang**

- Yes authority(1.3)yes and the CIA agent is very handsome(laughter)and he will protect people and help people in danger.

**speaker# Jiang**

- hum(0.6) A very a man of positive energy and[/yes]and good fitness good fitness

**speaker# Yang**

- Yes that's true(1.1)so(0.6) what’s your another TV series?

**speaker# Jiang**

- uh(1.3)I think I think is “Xin Shi Jie” It is about hum the 3 men in the 22 days before the peaceful liberation of Beipinng, [and they[/yeah]they made their own choices in the 22 days. And one of the three assist the Communist Party uh to uh[yeah] peacefully liberate Beiping[yes] and revealed a new world.

**speaker# Yang**

- Yes. And they don't want to destroy the attractions

**speaker# Jiang**

- hum yes

**speaker# Yang**

- yeah Ok That's all I want to share with you .

**speaker# Jiang**

- hum that's mine Now we have finished.
