# Supplementary material for: Multimodal Irregular Self-Selection in Chinese Postgraduate English as a Foreign Language Learners’ Conversation: When, How, and Why
Source: Front Psychol. 2022 Mar 25;13:788438. doi: 10.3389/fpsyg.2022.788438 (PMC8990892; doi:10.3389/fpsyg.2022.788438)
Supplement: Supplementary file 3 [file Data_Sheet_1.zip › Transcribed data/Group 20.docx]

***Supplementary Material***

**speaker# Gao**

- uh Several days ago there was a fierce discussion online about a movie star who received plastic surgery(0.3). and Have you ever notice that?

**speaker# Wang**

- (0.4)ah yes I’ve heard that.hum Actually hum several years ago I’ve read a newspaper hum It’s said hum Beijing Film Academy teachers and professors, they re(0.4)recruited students uh in the entrance exam, they hum(0.3)prefer students who uh who are not get plastic surgery. uh Because they think these uh students have natural beauty.

**speaker# Gao**

- (0.9)Wow those who apply for Beijing film academy(0.4)are only 18 years old or even younger(0.5)and(0.4)Such young generations are pursuing plastic surgery It’s astonishing(0.6) but admittedly, it's common for high school graduates to take(0.6)double eyelid operations(0.3). And I think to some extend this is a kind of mini plastic surgery.

**speaker# Wang**

- (0.9)uh yes(0.3)hum Nowadays there are many(0.4)uh more and more people uh to choose to get plastic surgery,uh and also the people hum they have the different opinions about this. Some people may think(0.3)uh they would like to choose to uh get plastic surgery because they are(0.3)because they think uh it can help them uh become(0.3)more beautiful and handsome uh so that they can attractive other(0.4)uh other people’s uh attention on to them. and they(0.3)in the contrast other people may think uh plastic surgery is a cheating,uh because uh once they uh(0.5)they choose to get plastic surgery uh they will lose their own uniqual characteristics.

**speaker# Gao**

- (0.6)Yes but a(0.5)beautiful appearance is now held at lofty place nowadays(0.4). And(0.3)Many people including myself(0.4) may make a quick judgement on others(0.6)who we first met(0.6)and only based on their appearances.

**speaker# Wang**

- (0.8)hum Yes I agree with you. hum Especially(crack)in our society hum once we uh find(0.6)hum once we find a job, or take part in some exams(0.4)hum(crack)(0.4)if we are more beautiful or handsome, uh probably uh the teachers or professors will have a(0.3)uh good impression on us in the first meeting.

**speaker# Gao**

- (0.6)Yes I agree with you. uh Plastic surgery has its own values and advantages. For instance uh we can obtain more confidence and self-esteem by taking plastic surgeries(0.4). And you know some people are(0.3)they are born with(0.4) undesirable congenital defects, such as cleft lip(0.3)they have to take plastic surgery to(0.4)cover their congenital shortages(0.3)and to get rid of those(0.6)unpleasant judgements from others.

**speaker# Wang**

- (0.5)uh Yes(0.3)uh like what you said(0.3)hum I have a question: if you have a you have a chance, would you like to get plastic surgery?

**speaker# Gao**

- (0.5)Well(0.3)even if I have a chance I have enough money I will not I will never try plastic surgery because(0.3)I am a person really afraid of physical pain.

**speaker# Wang**

- (1.1)yes hum Actually in my opinion, hum I think that uh those people who get plastic surgery they also face uh the huge risks during the surgery. uh Especially if the hospitals are not standard(0.3),uh the patients(0.3)uh will face the(0.3)hum(0.3)(crack)uh bad uh bad effects on them. And the surgery uh And the surgery uh sequel uh will also dange uh(0.5)dangerful for their whole life.

**speaker# Gao**

- (0.7)Yes uh actually except for plastic surgery, there are many other ways for us to get more beautiful(0.4).uh For example we can take exercises every day to lose weight. We can also try make up to become more attractive.

**speaker# Wang**

- (0.9)uh yes(0.3)But hum generally speaking for most of us uh I think it’s difficult for us to stick uh doing exercises every day. Because uh we usually uh think doing exercises hum may easily uh get tired, uh maybe some people choose to get plastic surgery uh they may have an idea that uh plastic surgery will help them became beautiful or handsome in a short time. uh And(0.4)hum(0.6)hum Besides uh in our uh society now, uh(0.6)plastic surgery with technical and uh surgical skills(0.3)uh has been improved a lot now. uh So the safe plastic surgery, uh also we can choose it, and(0.4)hum(0.7)(crack) we can uh(0.8)pass the uh we can uh get more beautiful and handsome uh(0.4)through this surgery.

**speaker# Gao**

- (0.6)yes(0.3)uh There are cases where people enhance their physical beauty(0.3)by(0.3)receiving operations of double eyelid(0.3)opening canthus an *:* d liposuction and in just a very short period(0.5) And With this(0.4)uh(0.5)changes with this changes in a very short period(0.3)they can find(0.5)uh their desirable jobs with more satisfactory income.

**speaker# Wang**

- (0.9)uh Yes uh but(0.4)(crack)(0.5)in my opinion uh inner beautiful is more uh is more uh important for us(0.3)uh(0.3)we can see uh on the TV we can see those uh models they are very slim, and they won’t uh won't(0.6)choose to get plastic surgery. uh Because they have their own uh(0.4)temperament, and the the audience still uh(0.4)are attracted by their temperament. So I think we can learn from them uh in our daily life, we can read more books or hum acquire more knowledges uh to improve our own temperament, so in this way we can also make more friends to help us.

**speaker# Gao**

- (0.6)yes uh in summer plastic surgery is a double ( ) thought(0.5)and But(0.5)uh actually uh Whether to take plastic surgery or not is a subjective choice(0.3), and everyone has the right to make the decision(0.5). I just want to remind us that before making the decision we should(0.6)weight the advantages and disadvantages of taking plastic surgeries(0.7) seriously(0.4). So today we have had an enjoyable speech(0.4)and See you next time.

**speaker# Wang**

- Ok see you. bye bye

**speaker# Gao**

- Bye bye
