# Supplementary material for: Multimodal Irregular Self-Selection in Chinese Postgraduate English as a Foreign Language Learners’ Conversation: When, How, and Why
Source: Front Psychol. 2022 Mar 25;13:788438. doi: 10.3389/fpsyg.2022.788438 (PMC8990892; doi:10.3389/fpsyg.2022.788438)
Supplement: Supplementary file 3 [file Data_Sheet_1.zip › Transcribed data/Group 3.docx]

***Supplementary Material***

**speaker# Han**

- So you said that you want to be an interpreter?

**speaker# Yu**

- Yeah

**speaker# Han**

- So hum why do you want to be an interpreter

**speaker# Yu**

- Yes Because I think a qualified interpreter can help people from different countries to understand each other[hum], hum understand each other's words or maybe to learn about the culture, and some customs, some traditions of their each other's countries. So I think it’s a very sacred job, a sacred career[hum]. And hum I think it a qualified interpreter much take much responsibilities[hum]so hum about these I think I my experience is just not enough [to]

**speaker# Han**

- [yeah]you need to continue

**speaker# Yu**

- Yeah so[of course]hum in order to achieve dream I still to work harder and harder[hum]yeah[I think]and I hear about that you want to be [a teacher in the future].

**speaker# Han**

- [Yes a]English teacher Yeah I want to be an English teacher in the future, uh because I think it is a great job. hum Teachers not only uh teach knowledge[hum], but also teach students how to be a man[yeah], how to behave well in society[hum]. hum l also think uh teachers can hum(0.8)how to say they uh like a guide [to students[/yeah]yeah in their in students life[yeah]. and hum In addition, I also like uh staying with kids. uh They are very active and innocent. All these[/yeah] make me love this job[yes]. I hope I can achieve my dream one day.

**speaker# Yu**

- Yeah so you want to be a teacher. So have you some uh did you have some maybe some hum example teacher [in your]

**speaker# Han**

- [uh]you know yes here is one[hum].uh She is my English teacher in senior high school.

**speaker# Yu**

- uh senior high school

**speaker# Han**

- Yes hum She taught me for three years. And during these three years I really learnt a lot from her[hum]. uh She is really a kind person. And she hum treated all students uh very patiently[hum]. uh She helped us solve problems. and no matter uh you have a good grade or a bad grade, she always treated us very equally. And every morning l remember, that every morning she will come to the classroom and[/ah] help us yeah study uh in the morning.

**speaker# Yu**

- uh she is [so wonderful]

**speaker# Han**

- [yes]hum For example reading hum some English uh the

**speaker# Yu**

- Passage?

**speaker# Han**

- Yes passage text yeah hum So she really helped us a lot [in English[/yeah].

**speaker# Yu**

- Yeah You know that in my college, I also major in the normal English English[/hum] Normal[yeah]. so At that time, when I young when I was a children, I also want to be a teacher in the future. But after exposing to the uh maybe the translation and interpretation[hum], I found that my interests maybe in yeah

**speaker# Han**

- in the

**speaker# Yu**

- Translation so[/yeah] I change my dreams.

**speaker# Han**

- Why do you change your dream?

**speaker# Yu**

- Maybe I'm more interested in the translation than[/hum] the a teacher[oh].

**speaker# Han**

- Yes yes translation. hum yes Although I think it's really hard[yeah], there are also many good uh and excellent translators and interpreters[yeah]. Yes

**speaker# Yu**

- so hum Do you have a boyfriend?

**speaker# Han + speaker# Yu**

- **1:** hohoo uh [I I never]I never have uh [have boyfriend before] Yeah
  **2:** [have you ever dream about it][never have?]

**speaker# Yu**

- So have you ever maybe think about thought about dream about your future boyfriend?

**speaker# Han**

- Future boyfriend? hum yeah Maybe, maybe I I want to have a boyfriend in the future.

**speaker# Yu**

- So maybe do you have some maybe standards [for your[/uh] future boyfriend?

**speaker# Han**

- uh hum let me think[hum]. uh yeah I hope he will be hum smart and humorous[yeah]. And he must desire to better himself[yeah]. I think that’s very important. hum And he hum he is supposed to treat me equally[hum] and give respect and support to me[yeah]. hum I think I uh both of us can become better[hum]hum with uh through the mutual uh understanding[yes]. yes So now how about you?

**speaker# Yu**

- hum

**speaker# Han**

- Do Have you ever been in love before?

**speaker# Yu**

- Yes I have one experience when I was in my senior high school.

**speaker# Han + speaker# Yu**

- **1:** oh [senior high school]
  **2:** [I know I'm so little] early [young]

**speaker# Han**

- [so]early young

**speaker# Yu**

- I think at that time I'm a little too young to understand about maybe the love[hum] so it’s a little yeah a little too early[yeah]

**speaker# Han**

- But maybe uh it brings uh a lot of good memories.

**speaker# Yu**

- hum maybe[yeah]uh Just Just that[hum]. Not good and not bad[hum].hum

**speaker# Han**

- Now uh if you have a boyfriend in the future, what[/hum] uh(0.5) are you

**speaker# Yu**

- Yeah yeah yeah I *((Stutter))* always often yeah dream of my boyfriend[yeah] yeah and but I think the most important thing is the maybe the feeling yeah[/hum] Feeling is the most important. but Before I meet him, hum I have many maybe many standards, maybe uh he is[/hum] very tall[hum]he must have two small eyes, I prefer small eyes.

**speaker# Han**

- Small eyes

**speaker# Yu**

- Yeah and I think he must be a person full of[hum] responsibility and he must uh be filial to my parents, my relatives and also[/hum]be filial to his own parents. and We must understand each other[hum] and trust each other. So I think it's very important. But all these standards just my imagination maybe just my own thinking. So[/hum] I think when I meet him when I meet him in the future, all these [standards]

**speaker# Han**

- [standard] maybe

**speaker# Yu**

- Just go away

**speaker# Han**

- It’s not that important at all.

**speaker# Yu**

- Yeah not important

**speaker# Han + speaker# Yu**

- **1:** and you Wow, he is my [Mr. Right]
  **2:** [Mr. Right]yeah yeah

**speaker# Han**

- hum

**speaker# Yu**

- I hope all of us can[can] find a Mr. Right.

**speaker# Han**

- Yeah A good man[yeah]. and I hum I think we need to expand our friends circle. [you know[/yeah] In college, uh I [study in the normal [study in the normal]university[yeah], and again I we come to normal university again. There are really few boys[yeah] you can meet.

**speaker# Yu + speaker# Han**

- **1:** Yeah we don’t have many opportunities to [meet some[/yeah] meet some maybe some boys[yes] [hum just]
  **2:** [It’s so hard]

**speaker# Yu**

- Yes so hard so we need to meet more boys expend our circle's freiend.

**speaker# Han**

- We need o make more friends. and I like making friends.

**speaker# Yu**

- Yes yes[/hum]

**speaker# Han**

- (0.8)hum so about the future uh I think

**speaker# Yu**

- Do you want baby[uh] Have you ever thought of your future baby?

**speaker# Han**

- Baby No I don’t want to have a baby.

**speaker# Yu**

- uh you don't want a baby?

**speaker# Han**

- I don’t want to have a baby really

**speaker# Yu**

- Why I think I think they are very cute and a symbol of [love]

**speaker# Han**

- [Yes]Baby is cute[yeah]. I’m not saying I don’t like kids, you know[/hum] but uh but you know hum after you have a baby[hum], uh it will affect your health and[/yeah] may cause some mental problems[yeah]. I know that many women[hum] had bad health[hum] after they have a baby. Also they are out of shape[yes yes]out of shaping and they have some depression[yeah]. hum And you need to spend most of your time taking care of your baby. hum So you lose you lost most of your own time and space[hum]. You and your husband need to take care of your baby. hum and You will get tired. so[/yeah]Sometimes it may cause argue uh[yes]with each other. I can’t stand this.

**speaker# Yu + speaker# Han**

- **1:** You are right. But I think hum a baby we need to have a baby not need to uh just a little beyond my imagination, I think we should have a baby it's hum a symbol of love so [I know in the modern]
  **2:** [hum a symbol of love]

**speaker# Yu**

- In the In the modern time many women hum don’t want a baby yeah[yes]maybe you have the[same]

**speaker# Han**

- [Just like]me[yeah]of course most of women want to have[yeah]do want to have a baby[yeah].

**speaker# Yu**

- I I just want have a baby Maybe I prefer a girl

**speaker# Han**

- A girl?

**speaker# Yu**

- Yeah yeah I think a girl is very sweet and very cute. so I can[/hum] buy many clothes and some[yeah] uh cute clothes to[hum]

**speaker# Han**

- For her

**speaker# Yu**

- For her yes I will uh make her very beautiful very cute I will make up for her everyday[hum]. And I know that hum the process of pregnancy is very difficult[/hard]yes

**speaker# Han**

- So long time[yeah] 10 months

**speaker# Yu**

- Ten month[yeah] and I will lose my weight out of shape[hum] but I think hum a baby can bring many love and happiness[hum] to family[yeah]yes So I think a baby is very important.

**speaker# Han**

- hum yeah I know that hum Lots of people may totally uh change to another person[yeah]after they have a baby[yeah].uh Like you you know, wow! I’m a dad or I’m a mom now! It’s really magic[yes]. yes and

**speaker# Yu**

- It’s a change of [our roles[/yeah].

**speaker# Han**

- Yeah Magic power[yes]totally change a person.

**speaker# Yu**

- So hum when I can see my children see my babys gradually grow up[yeah] I think it's very magic process[hum]So and when they grow up, they will meet many new things, just like us and

**speaker# Han**

- Experience

**speaker# Yu**

- Yeah They will have their own opinons,own friends and when they grow up, they will have their own boyfriend or girlfriend[hum], so yes it’s a very magic thing[yes]. So I think that[hum]I believe that when you have your Mr. Right when you got [married]

**speaker# Han**

- [Maybe] at that time, maybe I will change[/change] my idea. Maybe I’d like want to have a baby[/yeah]. I don't [know]

**speaker# Yu**

- [I] I don't I believe that yeah

**speaker# Han**

- Really

**speaker# Yu**

- Yes[hum]hum about the future, how about what do you think about our the science and technology maybe

**speaker# Han**

- Yeah Science and technology[hum]. uh It’s really advanced now[yes]. yeah it's already maybe and There are you know 5G[yes]. yeah Although I don’t have a 5G phone, I haven’t use it before,uh I know it's

**speaker# Yu**

- It will must have maybe a rapid connection

**speaker# Han**

- Yes yes rapid

**speaker# Yu**

- We will have less time to to

**speaker# Han**

- To download some files some videos[yes]. It will become very fast

**speaker# Yu**

- Yes It may it will be very convenient and can promote our maybe our life standards. And I know that in the future, that's maybe a 6G, 7G, 8G or even 10G

**speaker# Han**

- Yeah even 10G they will

**speaker# Yu**

- They will all come out[yes]yes

**speaker# Han**

- And(1.0)hum I also think of the(1.0) uh(1.0)the payment. Now we have hum WeChat pay and Alipay[yes], thanks to Jack Ma. Yes a great man, bring much convenience to our life. We can go anywhere just with a phone.

**speaker# Yu**

- Yes we don’t need to carry any cash.

**speaker# Han**

- Yes We just need scan the QR code[yeah]. We can pay for things[yes], anything we want to buy hum

**speaker# Yu**

- It’s vey convenient.

**speaker# Han**

- Yes[hum] hum(0.6) In the future, I think maybe uh there will be no cash.

**speaker# Yu**

- No cash. I also believe that[yes]. and Have your ever heard of maybe the driverless car?

**speaker# Han**

- Oh the driverless car[yes]. I have heard of this before[yeah]. You know uh I have read a news[/hum]. hum It said that hum the Tesla Company[hum]uh is making effort to test the driverless car[hum].and hum People are curious about this[/yes]. and They look forward to yeah the invention of a driverless car[yes]. but uh Then hum during their test a man was hit by the driverless car.

**speaker# Yu**

- Accident?

**speaker# Han**

- Accident Yes[/yeah] really. uh So people suddenly become yeah there uh there are a lot of fears about this.

**speaker# Yu**

- So the driveless car Maybe there are many uncertain factors [or some[/yes]unsecure[yeah] problems.

**speaker# Han**

- It’s not uh(0.9) not that safe[yes] safety problem.

**speaker# Yu**

- Yeah So maybe in the future all this maybe the unsecure elemets can be solved. So at that time, we can just sit in the car and we don’t need to drive it. and just siting them just sit yes [and it]

**speaker# Han**

- [It]comfortable

**speaker# Yu**

- Yes yes[yeah]

**speaker# Han**

- Yeah There are cars on the road, and(0.5)maybe there will be car in the sky.

**speaker# Han**

- Yes I remember there a there was a passage uh in the textbook[hum] textbook in my high school[yes]. uh It said that hum it is a dream[yeah] uh cars are flying in the sky.

**speaker# Yu**

- Yeah just like the birds[flying in the sky]

**speaker# Han**

- [Just like the birds] yeah and It saves a lot of space for us[yes]. Because you know nowadays the traffic congestions[yes] in the morning, the rush hours, yeah[yes] there are so many cars on the road.

**speaker# Yu**

- It can save space also save us time[yes] due to the traffic congestion.

**speaker# Han**

- Yes so we can we can hum make uh use the

**speaker# Yu**

- Outspace [sky space]

**speaker# Han**

- [Sky space]yeah the sky space so It will be a uh good thing, I think yeah

**speaker# Yu**

- Yes maybe at that time the pedestrian walk on the roads and cars just fly in the sky[yes]. yes uh beyond our imagination.

**speaker# Han**

- Yes I can't believe it[yes]oh yeah

**speaker# Yu**

- And you know that uh due to the outbreak of Covid-19[hum] many schools many colleges just adopt the remote learning.

**speaker# Han**

- Oh Yeah learning online.

**speaker# Yu**

- Yes Many people uh many students just use their phones[hum], to sit in their homes[yes] maybe They don’t need to go to school[yeah],yes it will reduce the risk of infecting Covid-19[hum] and it also hum maybe give students more time to learn in their homes[yes]. It’s a convenient way.

**speaker# Han**

- Yes[yeah]Really a convenient way[yes]We don’t need to uh go around all these places[yeah]. We just stay at home or anywhere[yeah], and then we can start to study.

**speaker# Yu**

- Yes yes it can maybe reduce the commute time[hum] we can study at any places[yes]. But I think maybe hum there are also some difficulties and problems.

**speaker# Han**

- Yeah I also think so.

**speaker# Yu**

- Yes As for me[hum] I prefer[hum]just sitting in the classroom to obtain the [knowledge].

**speaker# Han**

- [A real] classroom

**speaker# Yu**

- Yes yes We can hum communicate with our classmates[hum]and face to face communication.

**speaker# Han**

- Face to face.

**speaker# Yu**

- hum We can see our teacher just in front of us.

**speaker# Han**

- Yes A real person.

**speaker# Yu**

- Yeah so I think I prefer to sit in the classroom.

**speaker# Han**

- I agree with you[yes]. I hum I prefer yeah have a face to face communication with our teacher and classmates[yes]. and uh Because the remote learning cannot help us solve some of our problems[yes] immediately[yes]. yes Sometimes maybe the connection is not so

**speaker# Yu**

- Stable

**speaker# Han**

- So yes stable and It will waste some hum time in class[/yes].

**speaker# Yu**

- Yes and we can not hum just uh solve these problems timely[yes] yes

**speaker# Han**

- hum just uh Just in time, we cannot

**speaker# Yu + speaker# Han**

- **1:** hum yes And maybe in the future, you know that our country China a less developing country[yeah], so maybe in the future will grow up to be a [developed country].
  **2:** [developed country]

**speaker# Yu**

- And maybe at that time all the poor people can just escape poverty[yes].yeah All of us will have a better life maybe[hum]yes

**speaker# Han**

- Our life can be hum live standard[yeah]yeah can be improved[yes].and People h don’t need to worry about the food, and the clothes[clothes]. yesh Because I know that there are still some[yes] places in our country[yes], and there he people suffering[yes] from poverty[yes].

**speaker# Yu**

- So in the future maybe all these basic needs maybe uh satisfied[yes]yes.

**speaker# Han**

- I I'm confident[yes] in that

**speaker# Yu**

- Yes me too

**speaker# Han**

- Yeah I'm proud of uh yes our country[yes], there are so many great great people[yeah]. yeah(1.4) and Ok so maybe I think that’s all about today’s topic.

**speaker# Yu**

- Ok

**speaker# Han**

- Ok
