# Supplementary material for: Multimodal Irregular Self-Selection in Chinese Postgraduate English as a Foreign Language Learners’ Conversation: When, How, and Why
Source: Front Psychol. 2022 Mar 25;13:788438. doi: 10.3389/fpsyg.2022.788438 (PMC8990892; doi:10.3389/fpsyg.2022.788438)
Supplement: Supplementary file 3 [file Data_Sheet_1.zip › Transcribed data/Group 4.docx]

***Supplementary Material***

**speaker# Yang**

- hum OK but first, I want you to guess how many members are there in my family？

**speaker# Geng**

- Ok Since the one child policy and we have the similar age[yes] I guess maybe three of you.

**speaker# Yang**

- Sort of right. And hum they are my father, my mother[ok] and myself, and there are also three pretty lovely dogs in my family[oh], I often interact with them if I just at home. And in our spare time, we often go to the movies together and if we have a long vacation together, we may choose go to another city to seek for some delicious food. Because all of us like eating food that taste very well. So how about your family, Villanelle?

**speaker# Geng**

- OK so as a little bit different from yours. and My parents they worked at different cities, while I study at another city. so at a time that we can go home together, we never spend so much time outside You know we spent a lot of time doing something simple things like, watching TVs, or just walking around to see the schools that I had been to when I was a little girl. And also we may visit some friends of my parents. So that’s a part that we only do something real simple. And hum I really enjoy the time when we get together with each other. And also my grandparents live near our home so the time that we go home they will really happy about that. So in the future if I have the chance to move in the another city, maybe I will invite all of them to visit my house as well. and that’s a part of that we talk about the conditions in China so do you have any ideas that the family who live abroad. do you have any experience of that?

**speaker# Yang**

- Actually I haven’t been to abroad before I have seen many American TV shows that talk about family[ok] like desperate housewife. In that show the protagonist named(1.1)Jack and Susan. They are couples before but as time goes by, they because of some subtle matters, their relationship has changed. So they find that they didn’t love each other anymore, and the day they found this point they chose to get divorced immediately and they[ok] chose to uh talk to their children about this matter and they just want to end this relationship with peace just not not do quarrels or something like that[ok], and I think in China may be some couples will choose to compromise because of their children, they want their children have a complete family[yeah] until they go to university[hum]yeah

**speaker# Geng**

- (0.7)So I think that’s really brave for these couples, and for me that I haven’t been to abroad as well. And I want to talk about family conditions in total another city uh in the country called the Downton Abbey[hum], I recently watched it. It talks about that such a huge family, that happened between the 1904 and until the 1940s, So they have experienced the World War II, and they experienced so much disasters like they lost their family members. They have to face more changes to innovate the way that they regulate in the whole Abbey. And during this process some people may think that why did I have the opportunities to work at this house why you have the higher social status than me. and so this fighter is just going on in this show, hum I want to say I really admire the courage they have because it's such a huge house and so much affairs. And also I see as a part of the higher social status they still facing so much difficulties. and They have to manage that and also offer the more jobs and so so much kind of things like build hospitals. so maybe this story is really far from now, but I still think it has the qualities in England. So there’s the part of my talking, so do you have other stories that happened in America as well?

**speaker# Yang**

- hum I think another TV show that impress me most maybe the Big Bang Theory[hum]. Have you seen that before?

**speaker# Geng + speaker# Yang**

- **1:** Of course [it's really good]
  **2:** [yes It really very] interesting right

**speaker# Yang**

- And the Sheldon and Lenard, it can be said that they are not families at all, they are just friends or colleagues at beginning[hum], and after they have suffered up-and-downs, their(0.4) relationship has gradually become sort of can be said like families[yeah], yes I like the uh patterns they(0.9)interact with each other. And about how about you？

**speaker# Geng**

- So actually I really like the show and it talks about a different definition of family. They don’t have the related blood but they have close friendship as well. And another part I like the show is the friendship between Sheldon and penny, Actually Penny is kind of weird in this group. because She is not genius and also she is not always have good jobs or doing something really good. Then While Sheldon is hard to get along with, but he is kind, he is always honest with his friends, while Penny has some maternal qualities. She want to take care of all of her friends, so in that relationship I think maybe they are not the relatives or something else but they care about each other. And that’s the way that's the family I want to seek. So(0.6) we talking about the healthy relationship. So what qualities do you think we should have we maintain a healthy uh relationship?

**speaker# Yang**

- I think I may put trust at the first place[ok], because we all live in the community. If we lost the trust for each other, maybe that will uh cause some bad things and like our young generations young couples. hum I think many female one will choose to check their husband or boyfriend's phone to see something that didn't even exist, like they will say have you cheated on me or just betray me? and I think that(0.4)this matter this behavior[hum] will cause quarrel or fight and finally they will going to the divorce part.

**speaker# Geng**

- Yeah I couldn’t agree with you more, hum actually sometimes some female may do something a little bit we call stupid but that kind of thing just caused things even worse.

**speaker# Yang**

- Yes

**speaker# Geng**

- And the quality I want to mention is communication[hum]. well hum when we think about a married couple I mean seven years later and especially they have their childrens. They will put more things into their affairs, their jobs, hum their children’s homework or whatever. but they so they have less time to spend on themselves to take care of themselves. Especially take care of the relationship between the two couples. So I want to mention an example of my parents, hum they worked at different cities but they keep connection with each other. even some little just a little affairs about what I have eaten today? hum What did I drink what people did I met today[hum] just like these little things but they share with each other. and I think that’s the trick they can maintain a healthy uh communication and also maintain a healthy relationship. so If we do not have these kind of things, may be even the closest uh couples may fell apart[yes]at last. so that’s a part of it. hum I mean at our age we may think about the marriage in the future, so do you have any uh expectations about your future family?

**speaker# Yang**

- (0.9)hum As I mentioned before, I’m now 23 years old, and maybe 28 or 29 I will choose to get married[oh]. But I’m single now right. And[/hum] as for the children maybe no or just one, because I think if you have the baby you must take on the responsibilities to take care of them. But I don’t think I’m so mature, so I don’t(0.7)want the baby now. Maybe later I will change my mind. And as to the husband, just the person that really loves me[hum]. That’s OK. And I want to ask for more advise from you, is that how to balance the relationship between the work and your family.

**speaker# Geng**

- (0.9)Well I’m single too but uh if I have the chance to seek someone that I want to spend my rest of my life with. this guy must hum the We have the right we have the similar hum hobbies and common some topics to talk about. well if you ask me about the how to balance about the family and work, hum I would say I have a chance to get pregnant, maybe I will put my family first. Because the children is kind the future generations of both of our couple, and we should take care of them because if I gave birth to it, I will take on more responsibilities for them[hum]. and uh for me work, I didn’t say uh give up. I will Maybe two years or three years later, I will just uh come through and come back to my job(0.6). Also I have my dreams as well and at time they both go to the uh schools, and they have their dreams they have their ideas and I need to respect their thoughts as well, So at that time maybe hum we will think about some kind of ideas or schedules with my husband. and then We make the whole things together. hum and also I want to say about that. So today’s conversation is really good. I[/yes] I didn’t think we can have that such a fruitful conversation. so What about next we at the same time we talk about it again.

**speaker# Yang**

- Well we can talk about the topic.
