# Supplementary material for: Multimodal Irregular Self-Selection in Chinese Postgraduate English as a Foreign Language Learners’ Conversation: When, How, and Why
Source: Front Psychol. 2022 Mar 25;13:788438. doi: 10.3389/fpsyg.2022.788438 (PMC8990892; doi:10.3389/fpsyg.2022.788438)
Supplement: Supplementary file 3 [file Data_Sheet_1.zip › Transcribed data/Group 5.docx]

***Supplementary Material***

**speaker# Zheng**

- hum Today we talk about how to learn English Well hum I uh firstly I hum talk about my uh experience. Uh I I really I recite English words uh on on an app called 墨墨背单词. I think I think recite words on the app uh is more is is easier uh than uh than than reciting words uh on the book. hum Because Because uh these apps hum have uh have the uh have has have(1.1) autonomous，autonomous reciting certain circles. uh So we can recite words more effect uh more effectively(1.8) hum and sometimes I also watch movies, uh(1.2) such such as such as shaw shank as reduction. How about you?

**speaker# Wang**

- hum uh As for my learning experience, I also recite words on the app[hum]the same as yours and choose this app because it can it can uh how to say yes, the the memory lines[oh] yeah they can give uh depend on the memory lines. They can show me which words should should recite again[hum] So that is a very uh useful device for me to memorize the words. And also I combine the uh word the word app. And uh memory memorize word on the notebook[hum] because I like uh writing something on a notebook. It It gives me a feeling that I have control these words[hum]. So this is how to memorize the word. And as for as for other hum other areas, other aspects, uh such as the listening and talking, I prefer the way that reading books or listening to music. And also will we watch some movies. So I think the best way of learning a language is to create your interests. hum Here I want to share a kind of experience of mine. So I recently I have learned English and French and also the Korean. So(laughter)and here uh the compare the comparison between French and Korean. I can see that interest is very important for me, because I really don't care much about the French movie. So I cannot get the idea[hum], the the the the style of that kind of movie. But talking about Korean movie is really really good thing.

**speaker# Zheng**

- Yes I I got it.

**speaker# Wang**

- Yes. So hum after watching so many TV series of Korean, I I have learned a lot of oral oral expressions of Korea. But[/hum] as French, I have to memorize again and get grammar. So it is more hum more difficult and more complex for me. So I think the interest is the best master.

**speaker# Zheng**

- uh You just said that hum sometimes we uh we can't get the hum ideas about uh French movies you have said you have[/yeah] you have saw. I uh have the same thought as as you because uh sometimes I uh when I hum watch movies, English movies, I also uh field feel that the cultures or thought are different from ours[yes]. uh So uh You uh and some others have introduced many uh American and English movies. hum but but I can't I can't uh watch them for a long time. hum and after uh after watching hum after watching for uh some days or for some weeks, I will I will hum give up it because I think I can't get their [/Int unclear] get their things. Yes. And you just decide that hum and I also observed that you like writing notes[yes] both inside and outside the classroom. uh So uh I think it is also a good idea to learn English well[yes]. hum And(0.9) uh can you uh can you tell me some some of your uh some movies or music uh You You like?

**speaker# Wang**

- (0.7)Ok Talking about English movies，that is uh my style my things. so hum(0.9) just as I said, I don't really fond of the French movies. But I really ready uh into the American or European the English movies. So Um how to say, well talking about movies, I would like to recommend the the Vampire diaries. 吸血鬼日记 I really really are fond of that kind of things. That godish things the witchess,the vampires uh unique horns, and werewolves something like that. So I chose this movie because it is kind of hum it has some combination uh between our daily life. So it talks about some other uh how to say uh some high school students daily lives. So a high school girl called Elena[hum], she fell uh felt love with a vampire called Stephen. So uh their life is in their high school. So their hum speech is much more uh is very how to say not so far, uh not so far from our daily life. So I think you can learn many expressions, many words in this movie. So I think this movies is [very good].

**speaker# Zheng**

- [hum hum I I]I just finished a movie like a movie you just said called 暮光之城

**speaker# Wang**

- Yes Yes

**speaker# Zheng**

- hum You Know But But I uh I also afraid some some things uh some situations in the movies which uh which are which are[very]

**speaker# Wang**

- [He]drinks humans' blood

**speaker# Zheng**

- uh very honorable[yeah]and the words I forget.

**speaker# Wang**

- Ok(laugther)

**speaker# Zheng**

- hum(0.8) and uh I think uh the the communicating with hum teachers, hum both both our supervisors and our uh our daily daily daily teachers uh inside the classroom it is very useful for uh for develop our English ability. hum(1.0)but but but I I think I don't like I don't like to uh talk with them uh both inside and outside the classroom. hum I think I I uh I afraid them a little a lot. And I don't have many common topics with them(0.9). uh yes

**speaker# Wang**

- I share the same thoughts with you.

**speaker# Zheng**

- And And I I will I can make it with them. I uh just topics always about some uh academic or or very(0.9)hum(0.5)or very authentic uh topics, uh not about my daily lives, my daily studies uh(laughter)

**speaker# Wang**

- Yes so hum(1.2) what other ways can learn English well according to your own experience?

**speaker# Zheng**

- (0.7)hum(0.6)I uh(0.9) uh now I just uh recite words about uh if for some uh for some tests uh for example uh(1.3)

**speaker# Wang**

- IELTS(1.0) the TOEFL Ah that's TEM8

**speaker# Zheng**

- uh TEM8 uh So I will study for it. uh(1.3) hum like uh(1.1) do the uh do the test, uh to the uh previous test, or hum listen to BBC or some other or some other videos. hum But I uh I just study it for uh some achievements, but not for interests, or for developing my ability.

**(no speaker)**

**speaker# Wang**

- And After almost three years or two year of learning English in this kind of interesting way, my father thought oh maybe it's time for my kid to hum test how she learned, whether she did it well. So She uh So he sent me to another to another another how to say uh instruction? How to say okay something liken English school[hum] English classess, another uh organization to teach us. So(0.7)I'm there(0.9). The teacher(0.4)showed me a kind of paper examination uh exam to exam how I learn English. I still remember the full score is 120. And can you guess how many score did I get?

**speaker# Zheng**

- Oh how

**speaker# Wang**

- Yeah. I really I I only got six[ah] uh six point of 120. So my parents really thought, oh what have she learned? hum(0.7) and then they thought maybe interest uh is not the only thing. She must have to learn how to speak and how to write English in a better way, something like uh more authority, authoritative way. So(0.7)then I have learned English uh I have learned the grammar in a more authentic way. So the teacher will taught us, the subjective, a word, the objective, this kind of sentence structure, and how to give other people the questions and the past tense and something like that. After one month(0.5) the 120 scores I I can I could get 110. So[/Wow] then I thought that is my interest in English(1.0). I[/yeah] could get higher score. And the higher score is my interest(laughter). That That is kind of a prize for me. So uh from the from that on, I really fond of English.

**speaker# Zheng**

- Yeah yes The high score is also a very very important motivation uh[/yes] for our students in our country.

**speaker# Wang**

- uh [yes]

**speaker# Zheng**

- [Yes] but(1.6) but I uh but I think hum just like the paper you did in the English class to exam your English ability uh the these papers are uh I used to exam our hum English proficiency in uh in uh in the following areas, just like grammar words and other some fixed forms, fixed forms. But uh but I think uh uh you you can uh you can uh meet the the situation like uh you you meet a foreigner. But the but(1.0) uh what uh the what they said is not very strictly accordance with the grammar we have learned.

**speaker# Zheng**

- So So uh So the uh the paper, I think the paper is not the hum(0.8)uh not the only way to exam our proficiency. I think uh the fluent the fluent or the fluent talk. The flute talk is very important because foreigners is not very uh uh not uh not very strictly clear about hum whether your your grammar is whether your grammar is very uh very

**speaker# Wang**

- Correct

**speaker# Zheng**

- Very correct. Yes. uh If you can uh talk with them fluently and I think hum(0.8)I think it is it is ok. And they are they also like to talk with you(0.8).

**speaker# Wang**

- Yes

**speaker# Zheng**

- Yes(laughter). and[/So]hum and I think uh learning English very well in our country is not a very easy thing because we don't uh don't(0.8)immersed immersed in a in a contact.So uh I think the contact is also a very important factor to determine determine our true English ability, not just[yes] not just like uh grammar and some others. hum So hum the immersion study, immersion study is hum is very important. For example, some uh schools uh will invite some uh foreign teachers to uh to uh teach us inside the classroom. So we can talk with the true foreigner, the true foreigner uh both inside and outside the classroom. And they can teach us hum uh some expressions uh in their country, not in our country, in our classroom. And uh Besides Besides uh communicate, besides communicating with them inside the classroom, we can also hum find some community uh opportunity uh opportunities uh to talk talk with them outside the classroom. hum(0.5) and uh to study more, to study more uh uh(1.3)true English expressions.

**speaker# Wang**

- Yes

**speaker# Zheng**

- yes(laughter)

**speaker# Wang**

- So Both all we have talked about learning English uh by reading the articles, reading books, or[/yes] watching movies, listening to music, and also talk talking to a foreigner[hum] or uh some kind of foreign context in our daily life[hum]. So I think hum we have talked so many ways of learning English. So[/yes] maybe that's all for today today's talking. Okay

**speaker# Zheng**

- Ok
