# Supplementary material for: Multimodal Irregular Self-Selection in Chinese Postgraduate English as a Foreign Language Learners’ Conversation: When, How, and Why
Source: Front Psychol. 2022 Mar 25;13:788438. doi: 10.3389/fpsyg.2022.788438 (PMC8990892; doi:10.3389/fpsyg.2022.788438)
Supplement: Supplementary file 3 [file Data_Sheet_1.zip › Transcribed data/Group 6.docx]

***Supplementary Material***

**speaker# Wang**

- So May. How’s it going?

**speaker# Liu**

- uh Fine. In these days I uh I’m taking some online courses.

**speaker# Wang**

- (0.4)hum well it sounds nice. So what kind of online courses do you take during these days?

**speaker# Liu**

- (0.3)uh I choose some online courses about computer using. You know uh maybe my major, I will uh sometimes I need to use it. So what about you recently?

**speaker# Wang**

- hum Recently uh most of the time I’m focusing on uh pragmatics. You know that is my major[hum]. So I have to take some online courses about it. Just the same as you.

**speaker# Liu**

- (0.4)Oh so what are your online courses about? uh Yesterday, I just took part in an online online meeting uh which is lectured uh by a very famous uh professor. And through the app uh Tencent meeting and I think it’s very convenient. uh The content is about corpus linguistic and also my teacher often introduces us hum such kind of online meeting in our Wechat group. I think uh it’s very good.

**speaker# Wang**

- hum Yeah. It sounds really so convenient. uh My courses are mainly about pragmatics, just as I just mentioned above. And my tutor also send uh recommend some uh conferences, meetings, uh literatures or some online courses for me. And he expected us to uh read them and learn them as quickly as possible. hum It’s really very useful because we can just sit on the table or just lie on the bed, hum use our tele- mobile phone, computer or iPad to uh see some uh various kinds of views and ideas, and some lectures of the famous scholars uh from all over the world. hum and it can save a lot plenty of money, and also there is no hum strict requirement on the environment. So it plays such an important role in our daily life, right?

**speaker# Liu**

- hum Right I agree with you. And with the development of modern society, uh the online courses have become a very popular trend in our nation and even in this world. And it makes our life more easier and gives us more opportunities to choose what we want to learn. During the class, we can communicate and respond to our hum teacher through the chat frame and also hum we can ask questions.

**speaker# Wang**

- Really? It can ask questions

**speaker# Liu**

- hum Yes We can ask questions. uh For example uh some days ago, I hum had an online meeting which is lectured by a very famous professor and I feel very confused with one question. So I(0.7)choose to uh ask him about this question through the chat frame and he answered my question uh and solved the problem very patiently. I think it’s very convenient.

**speaker# Wang**

- Wow it sounds nice. I didn’t know how to ask questions before. And thank you for telling me such a news. hum It seems that it’s really a very good way to take online courses uh for learning. And for me the uh when taking online courses, I’m I feel relatively more relaxed and more comfortable, and my self-confidence is increasing because behind the screen uh I think no one will pay so much attention on uh on me and on my face and on my behaviour and so on. So I think uh when I answered the questions of the professor uh in the class. It’s a very good choice.

**speaker# Liu**

- hum Yes that’s very uh I agree with you. and I think hum(0.9)uh(1.3)hum I think(1.4)hum(1.4)

**speaker# Wang**

- Do you think there are some function of the

**speaker# Liu**

- Oh oh uh I remembered. uh I You know there is a screen recording function in our computer(0.8)and uh when I encounter some uh difficulties, I will choose to record the online courses and to listen to it repeatedly after class. And hum besides this kind of online courses, there are also other kinds of online courses uh which takes the form of a video. and I think it’s more convenient because we can watch it at any time and for many times.

**speaker# Wang**

- (0.5)hum Yeah it’s really a good point. and For the first year of postgraduate, our foundation is really so weak and many terminologies hum seem so difficult and so strange to us. hum When listen to the course for the first time, it’s hum it's very difficult or even impossible for us to uh(0.8)uh understand them well for just the first time. So the screen recording recorded really plays an important role and it’s a nice function. And I remember there are some online free online courses in our database of our school for example, the New Oriental Courses which is recorded and broadcast it's a very good choice for learning.

**speaker# Liu**

- hum But I heard some people say uh that the online courses is not always a good learning way for all the people, for all the students uh because uh it needs students themselves to be more concentrated on the class, on the learning content. And also uh during the class, the teacher cannot directly observe what the students are doing. So hum Therefore if the students are less self-constrained, it will uh this learning way will not beneficial for their further learning.

**speaker# Wang**

- (0.5)Yeah It’s really actually we have stayed at home for such a long period and we didn’t communicate with each other uh and with our friends face to face. hum I think my communication ability is decreasing uh but the communication ability plays such an important role in our daily life, especially for us, because in the future we’are gonna be(0.5)become a teacher, a primary school teacher, a middle school teacher, or even in the college. hum We’ll always communicate with our students, and even their parents. So it’s very essential for us to gain the communication ability. hum So for this use I think the traditional class hum do much better than the online courses uh because it can help us to practice the this kind of ability.

**speaker# Liu**

- hum Yes uh Traditional courses sometimes really do better and I also heard some people say that uh the online courses is not suitable for all the students because some of the students may uh don’t have a telephone or computer. And others may lack a Wi-Fi. So hum before the implement of an online course it a number of questions need to be solved. So hum I think it’s really a regrettable defect of online courses.

**speaker# Wang**

- hum Yeah and do you remember at the last semester of our university life uh at that period, all the courses become the online courses. And also hum all the tasks at the end of the semester become the course paper(0.4). It is very hard to hum(0.4)uh have the tasks just as usual. If we just uh take the exams uh without any pleasure without any measure, there must be some cheatings on the examination. And it’s very unfair for uh the students who treat the examinations very seriously and prepare well and wish to have a good score.

**speaker# Liu**

- hum Yes uh but it’s really impressive that at the beginning of this year, an unknown virus uh suddenly killed many people and uh the whole nation came into the state of defending this virus, uh named Covid-19. At that time, we were uh senior students and we have many online courses for the because we must and we should uh learn at home for the safety of ourselves and also for other people, so hum I think it's hum sometimes it may be very uh Thanks to the rapid development of modern society, we can uh learn in this way and uh also contribute to uh this battle.

**speaker# Wang**

- (0.5)hum Yeah its really a very tough time. uh I still remember that uh we have stayed at home for such a long period, and we cannot go out, and we just uh stayed at home and cannot uh come back to our campus, or to uh say goodbye to our friends, to our teachers or even have a graduation photo. At that time, we just take some online courses. hum And do you remember at the last uh class of our uh college, at that time we just have a video call, and we just see each other through the online course apps with our teachers. I think it’s a very precious(0.6)hum time and precious memory during that period. hum Though we can just see each other through the cold network cable, uh I think it's uh our friendship and our hum life and memory becomes very warm during this hard period.

**speaker# Liu**

- hum Yes We cannot deny the fact that online courses hum have some defects. But anything has its counterpart, so I think online courses have a very big value for us to utilize.

**speaker# Wang**

- (0.6)Yeah I agree with you. I think although it still has a lot of disadvantages hum but it’s just the beginning of the development of the online courses. So I I believe that it will be better and better. And there must be some balance between the traditional class and also the online courses.

**speaker# Liu**

- hum Ok so that’s all.

**speaker# Wang**

- That’s all
