# Supplementary material for: Multimodal Irregular Self-Selection in Chinese Postgraduate English as a Foreign Language Learners’ Conversation: When, How, and Why
Source: Front Psychol. 2022 Mar 25;13:788438. doi: 10.3389/fpsyg.2022.788438 (PMC8990892; doi:10.3389/fpsyg.2022.788438)
Supplement: Supplementary file 3 [file Data_Sheet_1.zip › Transcribed data/Group 7.docx]

***Supplementary Material***

**speaker# Liu**

- hum I just read a news about California wildfire.

**speaker# Wang**

- Well I have heard that news as well. That was very horrible.

**speaker# Liu + speaker# Wang**

- **1:** (1.0)Yeah I think it's horrific. I can see the horrific scene we can see that so many uh(0.4)so many particles or some Uh(0.5)or some gas, some materials is filled in the sky and this kind of horrible sky we can, we can hum we(0.4)we can’t see some things clearly as before [because of the fire].
  **2:** [Yeah because]

**speaker# Wang + speaker# Liu**

- **1:** When uh when we uh see those things or their videos we have seen from the internet. uh The fire is like a hell fire I think. hum hell [hellfire].
  **2:** [hellfire]

**speaker# Wang**

- Hellfire Because It's like the picture under the hell.

**speaker# Liu**

- Ah

**speaker# Wang**

- The hell

**speaker# Liu**

- I know I know

**speaker# Wang**

- Yeah It’s very hum scared and uh the the every thing around the fire is dark. The dark and red uh mixture the very horrible picture. And some even some night citizens have commented uh that videos that it's like uh effects in the movie we have never seen before.

**speaker# Liu**

- (0.5)Yeah I think this is a kind of(0.7)maybe it is a kind of disaster filled people and some other *:* species like animals may[yeah] and area and we can see the hum they can’t play a normal life as before because the uh wild fires in there uh hum sadly, uh I think sadly not only the California, but also Australian, also[yeah] experienced wildfire [also the]

**speaker# Wang**

- [Just the] last year.

**speaker# Liu**

- Yeah just the last year hum

**speaker# Wang**

- I think the two continents’ wildfire hum are attributed to many factors. hum I think not only the we have known that the natural system have their own chaotic system, uh but I think recently, the human beings have made some activities which uh which destroy the balance of the balance of the ecosystem, so the lightning are happening. so thewildfire happens.

**speaker# Liu**

- (0.7)Yeah I think the hum why the wildfire happens so frequently and the frequency of wildfires can be attributed more to, attributed to more about the factors related to our human beings, not other not only focuses on the external factor like the environment.

**speaker# Wang**

- Yeah(1.2)So I think, the burning of those forest or woods have made some gases or dioxide. hum Those gases will uh increase the potential of global warming. uh Because Global warming is another important uh issues we have[hum]we have heard recently

**speaker# Liu + speaker# Wang**

- **1:** (0.6)[yeah]
  **2:** [yeah] because it is related to the environment.

**speaker# Liu**

- (1.5)Yeah and(0.4)hum, global warming is also global warming is also a hot topic[yeah] when it comes to the environmental protection. We always talk about it, and hum the global warming and I think it with, hum with increasing global warming, the hum it effects animals and other species living in the two polar size[yeah] especially, and because hum bacause with the increasing sea levels and animals living in both two sides, I think they, hum they experienced a hard life. and There is an imminent danger, imminent danger is approaching because of the increasing sea levels. They cannot find food, they can't find seafood[yeah] and they can’t find habits to rise to live, so hum this is a dangerous, this is a sad news to those animal[yeah]species

**speaker# Wang**

- (0.3)uh but I think not only species, uh the uh some citizens of some countries near the sides uh two polar sides uh also face some uh very negative effect of the global warming. Such as the northwest Europe or the Netherland, those city those uh people in the two cities have faced uh threaten of uh of being submerged because two cities’ sea levels are really high. so if the more ice are melting, more ice is melting the(0.3)sea level will higher the cities will be submerged as well. It’s very hum

**speaker# Liu + speaker# Wang**

- **1:** Yeah I know. [Because][/yeah]the uh, due to the global warming, the sea level is increasing and also it may pose uh effect on the animals, but also on some countries and especially some countries living in the, living near the Europe and they may be flooded[yeah], they will be elected by the water because the increasing level, and that they may be in the sea, and they may be indulged[yeah]. And also some hum I think this kind of situation can also experiences in some countries with relative lower sea level[and they also]
  **2:** [The Dutch]

**speaker# Liu**

- Yeah they also can like uh Dutch and the other island countries[yeah]they can also experienced this kind of I think disaster.

**speaker# Wang**

- (0.4)Yeah[/yeah] I see. hum So when we talk about global warming or the some coastal cities, I think we should pay more attention to the marine animals, sho uh those animals who live uh in the sea, because uh we have heard some news that, recently we have heard some news that some marine animals also facing some threats to uh have no place to uh, provide their living.

**speaker# Liu + speaker# Wang**

- **1:** (0.9)Yeah when talks about pollution(0.5)in the sea and also uh also this can be attributed to the hum I think white rubbish and I have seen a picture and a turtle is chocked is spurned with uh with some[ropes]
  **2:** [a plastic]

**speaker# Wang + speaker# Liu**

- **1:** [plastics]
  **2:** [plastics]plastic and some ropes. They, they, the turtle is surrounded[yeah]they are chocked by the ropes. And I think this kind of plastic ropes are

**speaker# Wang + speaker# Liu**

- **1:** [thrown]
  **2:** [thrown]by our [human beings].

**speaker# Wang**

- Yeah I think so.

**speaker# Liu**

- We use the more and we do not uh place them in the right place and we just put it into the sea[yeah]this kind of action, I think posing a great threat to the life the animals living in the sea.

**speaker# Wang**

- I think this picture uh tell us that human beings are gradually destroy the balanced life of those uh natural species, but I think maybe one day human beings can find a right way to live with uh some uh animals or forest some species with a the right, with a right pattern. hum Although you have mentioned that the turtle or some uh fish[hum]in the sea have died because of the uh thrown-away everyday life uh waste or rubbish. hum Fortunately, recently I have heard another news that in Hainan, there is the people in that city have made an artificial reef for the undersea uh(0.5) undersea species, hum they made this artificial reef with a(0.6) hum with a sunken ship. uh The ship have sinking uh before(0.5)several years, so people make this ship uh like a reef for fish to live and for them to(0.8) uh for them to avoid excessive fishing(0.3). I think it's a very good project.

**speaker# Liu + speaker# Wang**

- **1:** (0.6)Yeah yeah this hum this kind of project is so beneficial and I first uh listen about this kind of project. I think this is a [disbative]
  **2:** [creative]

**speaker# Liu**

- Yeah creative this is a silver lining maybe[/yeah] a hopeful, hum it's a kind of feasible way to solve the the the white pollution in the sea[yeah].hum I think this can be implemented in some hum in some areas and this can this can to extent provide our life. This is a kind of uh kind of new solution

**speaker# Wang**

- Yeah I think maybe uh in Hainan, people in that city have be improved their awarenesses of protecting environment, so they uh they make this project and uh find a new place for their marine species to live.

**speaker# Liu**

- (0.8)Yeah hum Hainan is a Hainan is a province surrounded by the sea[/yeah] so they first came up this kind of project. And I think after uh with in this day and age more and more people gradually uh aware of the importance of protecting our environment. So hum after Hainan I think, there will be more provinces come up hum specific measures[/yeah] to protect our environment, to solve this kind of problem. And this kind of specific measures and can be hum can be uh, can be put forward according to their[yeah], according to their own situation. And they can uh use this specific measure to solve their own their own pollution, and after that we can have a better life, a cleaner environment.

**speaker# Wang**

- Hum Well I think you have mentioned that uh province can put forward other creative project to protect their environment[hum]. hum I have heard like Beijing and Shanghai hum they have put forward the project like uh

**speaker# Liu + speaker# Wang**

- **1:** Ah [rubbish classification]
  **2:** [rubbish classification]

**speaker# Wang**

- Yeah [yeah] I think it's a new idea for people uh to uh, to remind remember or to have to improve their awareness of uh throwing. When they throwing the rubbish, they have the uh concept to uh classify the rubbish and avoid the hum avoid the pollution uh in our uh living living environment.

**speaker# Liu**

- (0.7)Yeah I have heard about this rubbish classification in just two years more[yeah]. And I think hum Beijing and shanghai they are the mega city big city in our country and they hum I think it’s like kind of pilot testing. And our country[/yeah]first implement the policy in these two city. And after that we can see the effectiveness of protecting our[yeah]environment and how people are willing to uh to engage in this kind of[yeah]campaign. And after that we can after seeing so many productive effective outcomes, then we can implement these kind of manner to more cities more provinces and so that we can hum we can have a better solution to deal with the rubbish. And this will this is also a big step for us to uh solve our the environmental[yeah]problem.

**speaker# Wang**

- I think hum this this kind of classification or project is is made uh by our own individual. So I think individual action is important. hum although many people think individual action is small and the advantages it bring uh it brought is so little, but uh individual action can also make make some hum make some uh advantages to protect protect our uh living environment, hum such as we can uh saving food and to saving water, saving uh paper we have used. Because hum this fundamental element is coming from our own natural world. Yeah I think so hum the environment environmental protection, protection is is not only dependent on the government intervention our implementation, but also depends on our own individuals in the country.

**speaker# Liu**

- (0.9)Yeah you hum what do you talk about you talk about the importance of our individual to protect our environment to improve the awareness of protecting environment hum yeah I think this is right because[/yeah] our society consists of individuals. And we[/yeah] individuals. uh if everyone in the society contribute a lot to our society then the society will also make a big and we also make a huge steps from protecting our environment. And I think but hum besides the level of individual and the level of society[yeah], and the level of government can also make some interventions or some administration, they can make some interventions about protecting environment. For example They can hum

**speaker# Wang**

- make some electric devices

**speaker# Liu**

- They can they can encourage those companies who uh who produce more electric vehicles. And so that there are less(0.4) there are less there are less and less hum waste exhausted.

**speaker# Wang**

- (0.3)Yeah I think hum compared with the gasoline-powered car uh with the with the new energy-powered car. This two kind of car uh has represent the two idea of human of people’s concept uh in the environmental protection. Because uh we can substitute the fossil uh fossil oils, uh with the uh with the solar energy or the water and other new uh new energy, I think it’s it’s can be implemented by the government policy or government encouragement. Because uh the company is higher than the individual. We individual cannot uh reach the company(laughter) uh decision.

**speaker# Liu**

- Ah yeah [/yeah] and you mention several sustainable energy like the uh like the solar energy like the hydro energy[yeah]. And this kind of energy is a kind they are also a sustainable energy[yeah]. They are new energy and the development and production. uh the production and use of this energy is hum also mirrors our efforts, our human's efforts to tackle this kind of problems. So we should also put more efforts and more hum maybe money to invest to do more research about how to improve the efficiency of using this kind of energy. And this kind of policy should I think it should be implemented by the higher level like the government and also[/yeah] the country like the even the kind of country -country cooperation[yeah]. It needs more efforts from uh several sites.

**speaker# Wang**

- (0.4)And I think uh just we have mentioned so much hum matters to uh to protect our environment, to find some creative energy and to use, as I I hope in the future we will hear the less and less news about the California wildfire. And we still we have long way to go in the uh environmental environmental protection. hum but we can and we have the willingness [hum]to solve this problem and make our own mother-the earth better and cleaner.

**speaker# Liu**

- Yeah I think hum like the environmental protection and the solution of reduce the wildfire to lessen the impact of global warming is a hot topic is recently. It's talk is talk about more than before. And when people are awareness of the seriousness of this kind of thing, we can uh with the development of the technology and we can develop, develop develop more energy, more new patterns to[yeah]hum to solve the problem, to find a balance between the environment and our human beings[yeah] so that they can keep the balance. uh keeping the ecological balance is important and because the uh because the natural resources is also uh it's also so hum a kind of source it's a kind of source it's kind of good sources for us and for especially for our generation[yeah]. So we should keep our environment clean and put more efforts to hum to invest more effort to keep a cleaner uh more sustainable environment in the future.

**speaker# Wang**

- hum well yes I agree with you[hum] what you have mentioned. hum And I think as a postgraduates, hum we individuals can do a lot of things to protect our around the environment hum by uh by uh, by by eating all of our foods uh tomorrow, uh by uh you using using public transit tomorrow instead of uh, instead of hum using a car too, go to another place.

**speaker# Liu**

- (0.7)Yeah hum the environment is uh the environment is uh a huge problem. But it also It is embedded in our daily life.uh We are post-graduates. We are students in the university and we still can do a lot of things to protect our environment. We can uh, we can save our paper and we can print out to a paper with two page and so on rather than just one paper one page in in this way we can also save our paper. And hum we can save water, we can save some resources, we can hum use system, we can use more public transportation. And uh to uh to uh to tell other people the importance of protecting our environment. We can also do a lot of some maybe small things. But all all of us make these kind of small things. It’s also a huge step for cultivating a more sustainable environment.

**speaker# Wang**

- Yeah
