# Supplementary material for: Multimodal Irregular Self-Selection in Chinese Postgraduate English as a Foreign Language Learners’ Conversation: When, How, and Why
Source: Front Psychol. 2022 Mar 25;13:788438. doi: 10.3389/fpsyg.2022.788438 (PMC8990892; doi:10.3389/fpsyg.2022.788438)
Supplement: Supplementary file 3 [file Data_Sheet_1.zip › Transcribed data/Group 8.docx]

***Supplementary Material***

**speaker# Wang**

- hum we have been the Northeast Normal University hum for about one month. hum how do you think about it?

**speaker# Teng**

- (0.5)Well when I arrived at this university I fell in love with this (0.4)Because I during this period I found the professors there are knowledgeable but modest. They are admirable, of course.

**speaker# Wang**

- hum Yes? I think that teaching is a very holy profession and the Teachers can influence hum influence one’s whole life.

**speaker# Teng**

- (0.5)Definitely. Tang uhm Tang Dynasty poet Han Yu once wrote The teacher proselytizes instructs dispels doubt (0.3)and which has always been the guild line for teachers. Today we may have more understanding of this profession. Why don’t we address this topic further

**speaker# Wang**

- (0.3)hum Yes hum can can I ask you hum you some teachers who influence you most in your life？

**speaker# Teng**

- (0.5)Well as a postgraduate I do encounter a lot of professors who inspired me at different stages of my life. For example there is a kind and intelligent teacher called Mr. Ji in my university who has a *:* profound influence on me.

**speaker# Wang**

- (0.4)hum Yes more details?

**speaker# Teng**

- (0.7)Well during the summer vacation of my junior year I was once depressed. (0.6)You know as we appro- approach graduation we will face many choices. (0.4)hum Then I just don't know what life is all about feeling confused about the future. I can’t even eat or sleep well.

**speaker# Wang**

- (0.4)oh, I am sorry to hear that.

**speaker# Teng**

- (0.6)But Mr. Ji communicated with me(0.3)and he enlightened me with words full of comfort. (0.3)We used to belong to the same party branch but then he was transferred to another department. I don’t know why I uh believe him very much so I told him all the doubts and grievances in my heart.

**speaker# Wang**

- (1.4)hum yes hum I think he is really a good teacher.

**speaker# Teng**

- (0.6)Yes [hum] And he uh he enlightened me with words full of hum wisdom and comfort. (0.7)and he just hum and uh perhaps in addition to his words what impressed me uh most was also his respect for us students.

**speaker# Wang**

- (0.4)hum For example?

**speaker# Teng**

- (0.6)One detail is that he poured me a glass of water. You know it's the first time I've seen a teacher who pour a uh pour water for us students. (0.4)uh It's no exaggeration to say that he was just like a timely rain giving me a hand when I was near the point of breakdown. An *:* d fortunately thanks to him I didn't continue to be depressed and I am full of hope for our life again.

**speaker# Wang**

- (0.3)hum Wow you meet such a good teacher.

**speaker# Teng**

- (0.3)Exactly (0.4)and you must have your favorite teacher.

**speaker# Wang**

- (0.6)hum Yes hum I have I want to talk about several teachers in [/a] my life.

**speaker# Teng**

- (0.6)a several teachers

**speaker# Wang**

- hum yes hum First I want to talk my parents. hum You know my parents are both teachers. hum In my childhood when they patted me gently on my head and praised me for my performance(0.3)hum I promised that I was going to be a teacher like them(0.4)hum inspiring students with passion and enthusiasm.

**speaker# Teng**

- (0.7)Well no wonder you have a special preference for teacher. Please go ahead.

**speaker# Wang**

- (0.4)hum Okay hum In my remember hum my parents are both good teachers. hum For example hum my mom is a very patient teacher with a strong sense of responsibility(0.3) hum (crack) I still remember hum once she promised to accompany me on my eighth birthday(0.3)but she failed to do so.hum On that day hum she one of her students was injured and She took care of her day and night until her parents came back(0.4)hum Through this event,hum I think that teaching is a very holy profession.

**speaker# Teng**

- (0.6)Wow Your mom is such a responsible teacher. And the other teacher?

**speaker# Wang**

- hum yes I’d like to another teacher is hum Miss Wang. uhm She is a lovely lady hum Her long hair and warm smile are her symbols. (0.3)hum When she teaches us linguistics in our university. hum I still remember that she always encouraged me when I meet difficulties. (0.4)hum Because of her lively explanation of linguistics(0.3)hum I gradually got some interests about this subject(0.4)hum That is also why I choose linguistics hum as my major.

**speaker# Teng**

- (0.5)Oh thanks to him(0.6)thanks to her we have an opportunity to meet here. hum But I wonder What qualities do you think a good teacher should possess?

**speaker# Wang**

- (0.5)hum I think the most important personality is the upright. (0.3)hum Because the teacher should hum set examples to students. hum What’s more the teacher should be knowledgeable hum especially for the subject that she teaches(0.4)hum The teacher should know how to communicate with students and know their hum demands. hum What about you?

**speaker# Teng**

- (0.6)Well from my point of view hum I think, the a good teacher should first should be first of all as I mentioned earlier, kind and intelligent(0.5) He he can always be there when students need it most and he can seize the best and most suitable opportunity to inspire students. (0.3)And secondly I am with you on this point(0.4)Uh that is a good teacher should also be knowledgeable that is to say he should have uh solid knowledge as well as excellent teaching ability so that he can answer us students' questions better and hum Last but not least he should respect and appreciate students and set an good example for us which will influences us in a subtle but significant way.

**speaker# Wang**

- (0.4) hum well you summed it very well!

**speaker# Teng**

- (0.3)Really? I’m fla flattered. Then uh good teachers uh are not all alike although they have many similarities. He or she may be kind(0.3)gentle or amiable whatever. Then what do you think of different types of teachers?

**speaker# Wang**

- hum yes hum As we all know uh the different uh teachers have different types and different personalities(0.4) hum Some teachers are humorous and kind [hum]. hum In this way, the students always want to communicated with them . While other teachers are strict and(0.6) hum serious. It seems that they always keep a long distance with students(0.4) hum I I think that the first kind of teacher is better. hum Because In this way students always want to make friends with them and communicate with them. hum As a result the teacher can on hum can know the students better and the students can learn more from the teachers.

**speaker# Teng**

- (0.8)Ok for my part uh I think a strict teacher is not always unpopular. Instead they may give students test first and lessons afterwards(0.4)hum The axiom “Talented students are trained by strict teacher” really makes sense(0.3) and while the gentle teachers tend to spoil the students. For immature students they tend to show no respect for their teachers because they think they are too familiar with their teachers to hum treat them with difference(0.3). Everything should be in moderation. So I think a teacher should temper force with mercy too.

**speaker# Wang**

- (0.4)uh Yes it is a matter of “degree” it’s also a problem that we should pay attention to.

**speaker# Teng**

- (0.3)Yes you got the point [hum]. Actually teachers are not the only ones at the stage, all those who have educated and guided us can be our teachers. For example our parents. Parents uh are the first and most influential teacher for students. An *:* d our friends, models, future leaders(0.3)uh and even people younger than us. because uh everyone has something worth learning.

**speaker# Wang**

- (0.3)Yes we can not only learn from others; we can also teach them that's also my dream.

**speaker# Teng**

- Huh. I *-((Stutter))* I also expect to be a teacher in the future not only teach students knowledge but also uh bring happiness and warmth to them. And the most importantly pass on the love my teacher gave us(0.4). And when it comes to this, do you have any teaching experiences so far?

**speaker# Wang**

- hum yes hum I I think that the experience is that. uh In summer holiday uh I helped the girl in my neighborhood. hum She is a primary school students hum I told her hum many problems that she met in her homework [hum]. hum I feel a hum I told her English many times, I feel a strong sense of achievement when I saw she made a great progress. hum I think it is a very value experience for me.

**speaker# Teng**

- Me, too. The precious experience with kids made me aware of the responsibility on the shoulder of us future teachers.

**speaker# Wang**

- uh yes hum Since we have the same dream. hum Let’s uh to *:* achieve our dreams.
