# Supplementary material for: Multimodal Irregular Self-Selection in Chinese Postgraduate English as a Foreign Language Learners’ Conversation: When, How, and Why
Source: Front Psychol. 2022 Mar 25;13:788438. doi: 10.3389/fpsyg.2022.788438 (PMC8990892; doi:10.3389/fpsyg.2022.788438)
Supplement: Supplementary file 3 [file Data_Sheet_1.zip › Transcribed data/Group 9.docx]

***Supplementary Material***

**speaker# Wang**

- So uh Let's just get into our topic. uh Say something about the internet. hum Do you have something uh to share with me?

**speaker# Li**

- (1.0)Yeah uh when it comes to internet. I think I have a lot of to say because I think internet really changes our life in in mny aspects. And I just remembered when I was young. and at the time the internet was very popular and so When need to buy something and I had to walk to the store and for shopping. hum you know uh Sometimes my choices are very limited. And I think I really can't buy uh buy the things I like. hum but Now and I and the internet was very convenient and I can sit here and to loving some shopping appliances. And then I can hum choose my the things I need. And it's so convenient, right?

**speaker# Wang**

- Yeah I totally agree with you. And I have the same feeling you know that with internet we can gain the Information or by the products are just uh from the from on online. And without the limitation about the space and time, uh it is quite benefit us a lot. uh and I have a deep feeling especially uh for our college students you know that uh in a third year, we should uh complete our thesis[yeah], and we should uh search a lot of studies or materials. So we can serve the internet and find the hum statistics we need. And we don't have to just check the books or go to the library. So so I think yeah I can I think it's a good aspect of the internet. But I have to admit it also allow some problems. hum Maybe sometimes it will uh do harm to our eyes, such as my eye glasses is. They are so big. you know I think thanks to the mobile phones because the internet there are so many things on the internet, so it attracts me a lot. So I will be observed on the mobile phones. uh Just getting a lot of hum attractvie things.

**speaker# Li**

- Yeah And I think it indeed has bad impact on me, for example. And hum when there's no cell phone and I have no access to internet. We have later time and go out for some outdoor exercises and hang up with my friends, with my family. Yeah it's really great. But you know now when we was free yeah I I just[hum]sit here and to play my phone yeah hum and I think I have put too much focus on the intent. It distract my attention for more importance thing such as that they remain relations with my friends and my parents. Yeah.

**speaker# Wang**

- (1.0)Yeah especially at some reunion period, such as festival Spring Festival. We just sat around the table, but especially our young people just play the phones. I think it's quite a pity.

**speaker# Li**

- Yeah and I think the reason that the internet has hum so much attractions attract us a lot is because there are so many, so much Information yeah[yeah] so for example, uh if you're uh if you're following some stars[hum] you know the international liberty and will uh search the internet and for more details about the stars[yeah]Yeah, and I think but sometimes I think hum most of things are we need to(1.1) you know they're not very true[yeah] it's not true.

**speaker# Wang**

- Yeah after all it is a virtual world you know the celebrity, the stars they are also uh they are brilliant same that, but we don't know the real of them the real thing of them. So we have to tell the evil or good things are by our own uh critical principles are all thinking. We cannot just believe all things on the internet, especially on uh I I haven't been involved in some cheating cases, but my friend they have been treated by, uh maybe we call it telecommunications cheat or fraud the fraud[yeah]. hum I think it's quite horrible you know that you just your money uh will be stealed on invisible method, you know[yeah] technology criminalss So uh cracy.

**speaker# Li**

- Yeah and as we know uh nowadays, there are more and more uh reports about techcommunication fraud yeah and I think and many victims have lost their money and their property and so on. So[yeah]yeah and I think that's the maybe what's bad in uh some bad impact. That the internet exists on our life. yeah but We can't uh deny that there are really some good things the internet brings to us.

**speaker# Wang**

- Yeah

**speaker# Li**

- Yeah

**speaker# Wang**

- (0.5)Such as uh () after mention it again[yeah] Baidu, Alibaba and Tencent[yeah] they are big shapes.

**speaker# Li**

- Yeah they are electronic gigants[yeah]I think I think most of my apps on my phone[hum]are belong to them. They're big the giants.[yeah]

**speaker# Wang**

- [The giants] yeah yeah and uh especially China has done a great job in this field, you know the mobile pay on

**speaker# Li**

- Payment[yeah]Ali pay that's[/yeah] so convenient and you know this attracts a lot of foreigners and they are say that yeah Ali pai is so convinent, but in their country they need to pay cash you know[hum] and it's not very convinient right.

**speaker# Wang**

- Yeah uh I remember our foreign teacher, uh she once said uh when she came to China, she can just use her phone to scan the QR Code[code]. Yeah It's quite amazing he thought.Yeah It is(1.1)hum.

**speaker# Li**

- (1.4)Yeah but so when uh when I saw the QR Code at the very first beginning, I was so interested in it because it was like very complicated yeah complicated patterns. and I think and I just wonder uh if there's someone could could imitate take it or draw image of it, but yeah can let's go back hum I think.

**speaker# Wang**

- Move on we can

**speaker# Li**

- Yeah do you know big data yeah it's I think it's

**speaker# Wang**

- (0.4)Yeah hum combined uh concern our major translation and interpreting hum I heard the corpus 数据库

**speaker# Li**

- Yeah

**speaker# Wang**

- Yeah it's a collection of collect collection of the hum terminology or some uh professional expressions are in different fields, maybe chemistry or medicine or law. and you know What we need then we can just search it and pick them up and use it in our articles. And it really saved a lot of time and energy. you know Our brain, our human people human beings capacity is limited, but the internet combines with the application and technology just help us achieve this.

**speaker# Li**

- Yeah. And I think it is the internet that connected all things, all people in the world together and you know when serve the internet and it will make collect your Information, but maybe some private Information and then will it can be uh collected and stored and yeah to build a big base for data collection. And I think[/yeah]that's and uh I think the computer and may analyze this data and to draw some very useful conclusions. but you know If we human beings uh do that on our own, and they may cost about more time and energy to do it. So[/yeah]the internet really helps.

**speaker# Wang**

- Helps a lot yeah[/yeah]. During this process on although we cannot see it or touch the internet is it is uh[Ok just][/everywhere] yeah as a meadium throught the technology and what else do you whant to share about the disadvantages or advantages you know It just like a double sord. Although it is a cliche, but it is.

**speaker# Li**

- (0.5)Yeah And to be honest and so uh when I was younger maybe 15 to 16 years old. And I just think uh if I after retiring and I'll living in a more silent village[hum]with very beautiful natural sceneries, hum but you knoe the problem is that the connection the internet connection maybe not very good[yeah].So and but now and I like to I prefer uh to live in the city, because I think I have I can get closer to internet maybe. And I I think those those attract me a lot.

**speaker# Wang**

- Yeah you get internet then you can touch the whole world. It combines you on others, and you're associated with each other closely.

**speaker# Li**

- Yeah and I think it's really and the internet covers a wide range of aspects in our life for our study and work our entertainment and so on[Yeah]. Yeah.

**speaker# Wang**

- No one is an isolated island. the internet especially it plays uh pivotal role of function in this world.

**speaker# Li**

- Yean but hum I really think sometimes uh I was uh I think the internet made me feel(0.9)you know uh I'm so worried about my uh personally you know personal[Infor]

**speaker# Wang**

- Privacy?

**speaker# Li + speaker# Wang**

- **1:** Yeah yes information[yeah][They]
  **2:** [They] will be spied

**speaker# Li**

- And yeah and you know uh When you open an appliance and it will ask whether you give it the right to visit your photos, your Information, your text message. I think I I like. So uh Why let them do this I think my personal Information will be you know

**speaker# Wang**

- (0.5)Yeah but it is already a fact that we are just(0.8) we are all clear you know[yeah] actually we are clear. Everyone can see us(0.7). If we are a valuable person, I I think. Yeah we cannot hum escape from this this hum bad or I don't know how to describe it just yeah.

**speaker# Li**

- Yeah[fact] you know there are some uh you know the face scanning

**speaker# Wang + speaker# Li**

- **1:** Face scanning [yeah].
  **2:** [yeah]Yeah That means[hum] you don't to and[identification]. yeah the camera will recognize your faces. Some features your face and[/yeah] for you. So probably and I I think it's maybe dangerous. you know Sometimes and maybe some of your food print will like this[yeah] and that's it may sometimes lock your door

**speaker# Wang**

- I think we have already move to a lot of high technology you know[yeah]. They are quite advance yeah so maybe we can stop here. Iternet is really a wide hum wide topic you know If we just talk about, we can not finish maybe for 2 hours[yeah]. Ok[yeah]Very happy to talk with you.
