# Supplementary material for: Multimodal Irregular Self-Selection in Chinese Postgraduate English as a Foreign Language Learners’ Conversation: When, How, and Why
Source: Front Psychol. 2022 Mar 25;13:788438. doi: 10.3389/fpsyg.2022.788438 (PMC8990892; doi:10.3389/fpsyg.2022.788438)
Supplement: Supplementary file 4 [file Data_Sheet_2.docx]

***Supplementary Material***

**Li & wang 9** Displaying knowledge 4/Agreement 1/Aiding 2/Cooperative completion 1

1. 5:34 TT Displaying knowledge

or where are you come from, that’s

Or, what’s your name

2. 6:53 (Failed) TT

So what do you want to be[ ] in the future

[You are] you are very social

3. 6:56 TT Agreement

You are very social and you are good at

Yes

Interpreting

4. 7: 46 TT Displaying knowledge

In the winter and summer, and I think

Yes, I think you can enjoy your self.

5-6. 9:39 TT & TT Aiding & Cooperative completion

May be my future hum

will be very [brilliant]

[brilliant]

7. 10:45 TH Displaying knowledge

I can hold parties many times hum

There must be a garden in your house

8. 13:07 TT Aiding

So may be cat will give me uh

A sense of safety

9. 15:40 TC Displaying knowledge

also I think I'm not qualified(1.7)[hum]

[but]after ten years will be(laughter). You'll be a qualified interpreter

**Yang & Jiang 18** Displaying knowledge 7、Cooperative completion 1、Agreement 1、Clarification 3、Aiding 2、Information request 1

1. 1:40 TT Displaying knowledge

And I think this spirit is hum remaining true to our first aspiration and[/yes] never giving up. hum and

I think so I think the competition must be very fierce(0.5).

2. 1:50 TC Displaying knowledge

uh Yes [and]

[Due to] the yes

3-4. 3:43 Failed & TT Displaying knowledge

A: They protect the Shanghai and the and the whole country you know [and I touched]

B: [Yes and when I think]

A：by their[competition]

B: [And I think] hum apart from patriotism, hum persistence and bravery is also a very uh a kind of spirit

5. 7:40 TC Displaying knowledge

And I also think hum our country should carry out some relevant laws to about this kind hum event so that our teenagers and even college student can be protected hum well(0.3) [That's my personal] understanding

[yes I think so] and I think the students should stand up to stop this phenomenon.

6. 8:52 TT Cooperative completion

the character acted[/yes] by the by [Zhou Dong Yu]

[/Zhou Dong Yu]

7. 10:55 Failed

I think the mean hum morality this movie want to show to us is to cherish our[/yes] present life[yes] [because]

[I think]

8. 12:05 TC Agreement

So he's very optimistic one. [hum and a] brave one.

[/Yes I think so]

9. 12:54 Failed

So [what]t is the main plot?

[and]

10. 13:12 TT Clarification

And he want to

Also the lung cancer?

11. 13:25 TT Clarification

So he(0.5)[pro-]

[You mean] leave her family a big fortune?

12. 13:42 TT Displaying knowledge

He produces uh the thing this kind of thing to

The drug yeah

13. 15:57 TH Displaying knowledge

and finally uh his husband was promoted to a engineer uh

Yeah Xu Huanshan

Yes hum in Shanghai

14. 16:41 TH Displaying knowledge

That's my hum understanding of this movie hum [Yes] [I]

[I think] when we live in Shanghai, there are too much pressure

15. 17:27 TT Information request

hum I think I will live in a

Where

16. 18:17 TH Clarification

It is so far away from the prosperous places [So]

[Yeah]you mean Hu He Haote

17. 18:42 TT Aiding

hum I think the challenges means more(2.3)

Means more chances

18. 20:40 TT Aiding

You mean the American

polities yes

**Yu & Han 38** Displaying knowledge 19、Agreement 6、Information request 3、Aiding 4、Cooperative completion 5

1. 0:52 TC Displaying knowledge

I think I my experience is just not enough [to]

[yeah]you need to continue

2. 0:55 TT Agreement

Yeah so

of course

hum in order to achieve dream I still to work harder and harder

3. 1:05 TT Agreement

I hear about that you want to be [a teacher in the future].

[Yes a]English teacher Yeah I want to be an English teacher in the future

4. 2:00 TC Displaying knowledge

So have you some uh did you have some maybe some hum example teacher [in your]

[uh]you know yes here is one

5. 2:51 TT Displaying knowledge

uh she is [so wonderful]

[yes]hum For example reading some English uh the

6. 2:52 TT Information request

For example reading some English uh the

Passage?

7-8. 3:28 TT & TT Aiding & Displaying knowledge

I found that my interests maybe in yeah

in the

Translation so[/yeah] I change my dreams.

9. 4:00 TH Information request

uh [I I never]I never have uh have boyfriend before Yeah

[have you ever dream about it] [never have?]

10-11. 5:17 TC & TT Displaying knowledge & Agreement

oh [senior high school]

[I know I'm so little] early [young]

[so]early young

12. 5:53 TT Displaying knowledge

Now uh if you have a boyfriend in the future, what[/hum] uh(0.5) are you

Yeah yeah yeah I ((Stutter)) always often yeah dream of my boyfriend

13-14 6:56 TT & TT Cooperative completion& Displaying knowledge

when I meet him in the future, all these [standards]

[standard] maybe

Just go away

15. 7:01 TT Cooperative completion

and you Wow, he is my [Mr. Right]

[Mr. Right]yeah yeah

16. 7:08 Failed

I hope all of us can

can

find a Mr. Right.

17. 7:24 TT Cooperative completion

uh I [study in the normal] university

[study in the normal]

18. 7:44 TC Displaying knowledge

Yeah we don’t have many opportunities to [meet some[/yeah] meet some maybe some boys

[hum just]

[It’s so hard]

19. 8:01 TT Information request

(0.8)hum so about the future uh I think

Do you want baby[uh] Have you ever thought of your future baby?

20. 8:18 TT Agreement

I think I think they are very cute and a symbol of [love]

[Yes]Baby is cute

21. 9:37 TT Agreement

I think we should have a baby it's hum a symbol of love so [I know in the modern]

[hum a symbol of love]

22. 9:45 TT Displaying knowledge

In the modern time many women hum don’t want a baby yeah[yes]maybe you have the[same]

[Just like]me

23. 10:07 TT Displaying knowledge

so I can[/hum] buy many clothes and some[yeah] uh cute clothes to

For her

24. 10:22 TT Displaying knowledge

I know that hum the process of pregnancy is very [difficult]

[hard]

25. 11:04 TT Displaying knowledge

It’s really magic

yes.

yes and

It’s a change of our roles

26. 11:26 TT Displaying knowledge

when they grow up, they will meet many new things, just like us and

Experience

27-28. 11:46 TT &TT Displaying knowledge & Cooperative completion

when you got [married]

[Maybe] at that time, maybe I will change[/change] my idea.

29. 11:54 TT Displaying knowledge

I don't [know]

[I] I don't I believe that yeah

30. 12:30 TT Displaying knowledge

I haven’t use it before, uh I know it's

It will must have maybe a rapid connection

31. 12:37 TT Aiding

We will have less time to to

To download some files some videos

32. 13:46 TH Displaying knowledge

We can pay for things[yes], anything we want to buy hum

It’s vey convenient.

33. 15:32 TC Displaying knowledge

just siting them just sit yes [and it]

[It]comfortable

34. 16:07 TC Agreement

just like the birds [flying in the sky]

[Just like the birds] yeah

35. 16:30 TT Aiding

so we can we can hum make uh use the

Outspace

36. 18:10 TT Displaying knowledge

I prefer[hum]just sitting in the classroom to obtain the [knowledge].

[A real] classroom

37 18:53 TT Aiding

Sometimes maybe the connection is not so so

Stable

38. 19:23 TT Cooperative completion

so maybe in the future will grow up to be a [developed country].

[developed country]

**Yang & Geng 1** Displaying knowledge 1

1. 5:15 TC Displaying knowledge

Have you seen that before?

Of course [it's really good]

[yes It really very] interesting right

**Wang & Zheng 6**  Displaying knowledge 3、Aiding 2

1. 5:33 TT Displaying knowledge

I think I can't get their

[unclear]

2. 8:00 TT Displaying knowledge

So I think this movies is [very good]

[hum hum I I]I just finished a movie

3. 8:22 TT Displaying knowledge

I also afraid some some things uh some situations in the movies which uh which are which are[very]

[He]drinks humans' blood

4. 10:35 TT Aiding

I just uh recite words about uh if for some uh for some tests uh for example uh(1.3)

IELTS(1.0) the TOEFL Ah that's TEM8

5. 18:45 TT Aiding

whether your grammar is very uh very

Correct

6. 19:07 Failed

[And] hum and I think uh learning English very well in our country is not a very easy thing

[So]

**Wang & Liu 2**  Aiding 1、Displaying knowledge 1

4:55 TT & TT ~~Displaying knowledge/~~Aiding & Displaying knowledge

I think hum(0.9)uh(1.3)hum I think(1.4)hum(1.4)

Do you think there are some function of the

Oh oh uh I remembered.

**Wang & Liu 12** Displaying knowledge 8/Cooperative completion 3/Agreement 1

1. 0:40 TC Displaying knowledge

we can’t see some things clearly as before [because of the fire].

[Yeah because] When uh when we uh see those things or their videos we have seen from the internet.

2. 0:54 TC Cooperative completion

uh The fire is like a hell fire I think hum hell [hellfire].

[hellfire]

3. 2:02 TC Displaying knowledge

I think sadly not only the California, but also Australian, also[yeah] experienced wildfire [also the]

[Just the] last year.

4. 2:05 TH Displaying knowledge

Yeah just the last year hum

I think the two continents’ wildfire hum are attributed to many factors.

5. 5:47 TT Displaying knowledge

It’s very hum

Yeah I know.

6. 6:30 TC Displaying knowledge

I think this kind of situation can also experiences in some countries with relative lower sea level [and they also]

[The Dutch]

7. 7:35 TT Displaying knowledge

I have seen a picture and a turtle is chocked is spurned with uh with some[ropes]

[a plastic]

8-9. 7:50 TT & TT Cooperative completion & Agreement

I think this kind of plastic ropes are

[thrown]

[thrown]by our [human beings].

[/yeah] I think so

10. 10:00 TT Displaying knowledge

I think this is a [disbative]

[creative]

11. 12:15 TT Cooperative completion

hum I have heard like Beijing and Shanghai hum they have put forward the project like uh

Ah [rubbish classification]

[rubbish classification]

12. 16:10 TT Displaying knowledge

For example They can hum

make some electric devices

**Teng & Wang 1**

1. 3:27 Failed

hum Yes hum I have I want to talk about several teachers [in] my life.

[/a]

(0.6)a several teachers

**Wang & Li 7** Displaying knowledge 5、Aiding 2

1. 7:03 TT Displaying knowledge

you know the mobile pay on

Payment[yeah]Ali pay

2. 7:43 TT Displaying knowledge

It is(1.1)hum(1.4).

Yeah but so when uh when I saw the QR Code at the very first beginning, I was so interested in it

3 8:17 TT Displaying knowledge

do you know big data yeah it's I think it's

Yeah hum combined uh concern our major translation and interpreting hum I heard the corpus 数据库

4. 10:24 TT Aiding

During this process on although we cannot see it or touch the internet is it is uh [Ok just]

[everywhere]

5. 12:28 TT Aiding

I'm so worried about my uh personally you know personal [Infor]

Privacy?

6. 12:32 TC Displaying knowledge

Yeah yes information [They]

[They] will be spied

7. 13:46 TT Displaying knowledge

you don't to and

[identification]

**Luo & Fu 5** Displaying knowledge 2、Agreement 1、Clarification 1、Information request 1

1. 1:55 TC Displaying knowledge

Yes[and]

[and]yes hum and hum people will also send their wishes from internet

2. 5:36 TC Agreement

Change is uh the wife of Houyi [uh]

[They are] a couple

3. 10:09 TT Clarification

hum Because uh I like the uh food(0.4)I think

you mean uh you mean the glutinous rice

4. 11:10 TH Information request

It's about niulang and zhinv uh

Can you tell me who is Niu Lang and who is Zhi Nv

5. 12:50 TC Displaying knowledge

hum yes and hum I today I'm very happy to talk with you[we]

[Me] too. I think I have learned a lot from you.

**Xu & Wang 9** Displaying knowledge 5、Aiding 3、Clarification 1

1. 0:33 TT Displaying knowledge

you know time will(1.6)[hum]time will

Yeah time really flies

2. 0:53 TT Aiding

I want to know why hum why you want to uh(0.9)go on uh

Yeah go on my acdemic study? here?

3. 2:30 TC Displaying knowledge

You never? [you never]

[yes]yes in the last year suddenly I naturally prepare for my postgraduate examination.

4. 5:09 TT Aiding

so if I want to be uh maybe a higher a higher

Higher skilled

5. 5:16 TT Aiding

I uh I have to(0.6)

Work hard

6. 5:56 TT Displaying knowledge

it is stable it is

Yes the same as my parents

7. 6:15 TH Displaying knowledge

they prefer a stable job[yeah]. hum [I]

In the career is more suitable for girls that's right?

8. 8:13 TT Clarification

And in fact hum

So hum you mean What’s in your mind?

9. 14:06 TC Displaying knowledge

I mean we are MTI(0.8)[but]

[There]are a few of English majors hum are hum can have their ability to pursue their position both translators and interpreters

**Liu & Su 8** Displaying knowledge 3、Clarification 1、Agreement 2

1-2. 0:53 TC & **Failed** Agreement

I know you play The Honor of Kings I don't know [how to say it]

[Yeah](1.3)why[/so] are you interesting I think it's a good game

3. 1:28 TC Displaying knowledge

we can form a good friendship with our hum game players [game player]

[Yeah]Because after like uh so many years of you know schooling and education experience

4. 1:55 **Failed**

Yes(0.9)so[/uh] I think we don't we don't hum play these games often.

5. 2:12 TC Clarification

hum it has some bad effects to our life(1.5) [do you think so]

[I think](0.6)You you mean the bad influence of video games that our parents told us I yes?

6. 5:35 TC Displaying knowledge

so I think these restrictions are necessary.(1.3)[It's why they are useless]

[Just because] Just because they are very young, so they don’t know how to control themselves

7. 7:38 TT Displaying knowledge

Maybe they just find their own thing. you know video[games]

[yes]So the interesting is very(1.0)is very important

8 7:47 TT Agreement

You mean the interest in [video games]

[interest yes]

**Ma & Xie 3** Agreement 1、Displaying knowledge 1、Aiding 1

1. 2:31 TT Agreement

he usually she usually do some small punishment to us uh then

Yes I agree with you yes

2. 8:23 TC Displaying knowledge

just a three three aspects of counties [I think].

[hum I think] the possible[yeah]their personality is far from what we have talked.

3. 8:57 TT Aiding

there are reports about the corporal(0.4)

punishment

**Shao & Zuo 5** Displaying knowledge 4、Cooperative completion 1

1. 2:53 TC Displaying knowledge

you can also listen some informal materials such as the Allen Show[yeah] or Friends[this] [yeah]they are popular.

2. 8:24 TT Displaying knowledge

I think the Economists hum the [it]

[Sometimes] it's too difficult for us

3. 8:39 TT Cooperative completion

let’s talk about the last part hum that is [speaking]

[speaking] Yeah

4. 9:24 TC Displaying knowledge

she speaks very well [English]

[and]elegant

5. 9:45 TC Displaying knowledge

such as the uh Evaca(0.4)[uh]

[Evaca] yeah really goddess

**Ke & Wang 6** Displaying knowledge 4、Information request 2

1-2. 1:35 TC & TC Information request & Displaying knowledge

So I decided to(0.7)hum find a part time job uh as a teacher [hum]

[teacher?]

Yeah [yes]

[I like] teacher

3. 8:56 TT Displaying knowledge

Have you imagine your wedding(0.5)wedding

No I ha[dn't]

[in the] future

4. 9:38 TT Information request

Can you understand how I scared when [at that time]

[Did] Did the custom uh got mad with you

5. 13:00 TH Displaying knowledge

I choose to be a tutor[hum]hum

and secondly as your second job

6. 14:37 TCDisplaying knowledge

I will I want to be a teacher[and]

[In]the future

**Liu & Song 4** Displaying knowledge 2、Agreement 2

1. 0:22 TC Displaying knowledge

I don’t think(0.5) about our future. [uh]

[why don’t you think so]

I don’t have plan to(0.3) get married

2. 2:44 TT Agreement

maybe[hum] my husband will hurt me[yes]. They are

They are afraid of getting hurt from[yeah] their partners[0.5].

3-4. 11:41 TT & TC Displaying knowledge & Agreement

So(clear throat) although the things effect are bad but I still(1.2) believe[hum] and hum struck to believe

The perfect marriage[right]

[yeah]

**Cai & Yang 18**  Displaying knowledge 7、Information request 9、Clarification 1、Aiding 1

1. 3:49 TC Displaying knowledge

My ideal life first of all, I think we I must have a job.

Yeah [that’s the basic]

[I must]I I must earn a lot of money

2. 4:35 TT Information request

That is very important [yeah] and

and a car?

3. 5:02 TT Information request

That is my ideal [life].

[hum] Speaking about your parents, would you live with your parents in your future?

4. 5:16 TT Information request

maybe the same

Same city?

5. 5:25 TT Displaying knowledge

maybe a same [neighbor]

[area]

6. 6:04 TT Information request

But don’t you think

Dangerous?

7. 6:12 TT Displaying knowledge

Have car maybe raising a car is [a little bit] EXPR?

[expensive]

8. 6:18 TT Displaying knowledge

That’s a deal but hum

So you are still choose to have a car

9. 7:17 TT Information request

I went to a big city with my friends, but we

Big city where(laughter)Which big city

10. 7:35 TT Information request

we choose[uh]

[Walking]?

11. 7:45 TT Information request

we waited a very long time, very very very long, so [in that]

[Is in] midnight?

12. 7:59 TT Information request

sometimes I think car is necessary but

But if you don’t have a car, which transportation would you like to go?

13. 8:12 TC Displaying knowledge

Walk[walk]just walk，[because my]

[Ok that's]healthy(laughter)

14. 9:29 TC Clarification

What kind of the way they study(0.8) [or]

[Studying?]

15. 11:10 TT ~~Displaying knowledge~~ Aiding

No no no he(1.2)

He just change his mind

16. 11:37 TT Displaying knowledge

Some of them didn't go to university but(1.0)

Oh my god I got it

17. 13:17 TT Displaying knowledge

maybe I don’t like studying, but I

You have to

18. 14:15 TT Information request

So (1.0)

So no more questions?

**Zhao & Yu None**

**Han & Wang None**

**Wang & Gao None**
